# Supplementary material for: Economics of healthcare access in low-income and middle-income countries: a protocol for a scoping review of the economic impacts of seeking healthcare on slum-dwellers compared with other city residents
Source: BMJ Open. 2021 Jul 9;11(7):e045441. doi: 10.1136/bmjopen-2020-045441 (PMC8273471; doi:10.1136/bmjopen-2020-045441)
Supplement: Supplementary data [file bmjopen-2020-045441supp001.pdf]

Supplementary material

The economics of health-care for slum-dwellers

Literature search

The aim of the search strategy was to identify studies examining the costs of healthcare for slum dwellers or city residents in low- and middle-income countries (LMIC). A search strategy was developed by an information specialist using Ovid MEDLINE. Search terms were gathered together for the following four key concepts: slum dwellers, urban areas, healthcare costs and LMIC. Both textword and subject heading searches for each concept were included in the strategy. Retrieval was limited to publications within the last 10 years. No langauage restrictions were applied. The final search strategy for MEDLINE was agreed by the review team and then translated as appropriate for the other databases and resources.

Databases containing literature from the fields of health, economics and social science were searched on 22nd June 2020: MEDLINE (Ovid), Embase (Ovid), EconLit (Ovid), Science Citation Index (Web of Science), Social Science Citation Index (Web of Science) and Global Index Medicus. In addition, the following resources containing grey literature such as theses and dissertations, working papers and reports were searched: Proquest Dissertations and Theses (A&I), Econpapers, OpenGrey, the World Bank website and the Organisation for Economic Co-operation and Development (OECD) website.

EndNote was used for reference management and duplicate removal. The search strategies for all databases and resources can be found in the appendix.

Appendix

The search strategies for MEDLINE and EMBASE incorporated a low- and middle-income countries (LMIC) search filter developed by the Effective Practice and Organisation of Care (EPOC) Cochrane group.[1] Where possible, this filter was translated for use in the other databases searched.

MEDLINE ALL
via Ovid http://ovidsp.ovid.com/
1946 to June 19, 2020
Search date: 22nd June 2020
Records retrieved: 2368
POPULATION
1 Urban Population/ (58856)
2 Cities/ (19180)
3 Urban Health/ (17793)
4 Urban Health Services/ (3613)
5 Hospitals, Urban/ (7341)
6 urban$.ti,ab. (156001)
7 (city or cities or megacity or megacities or metropolitan).ti,ab. (169349)
8 (metropolis or megalopolis or municipal$ or conurbation$ or suburb$ or town or towns or township$ or borough$ or barrio or barrios).ti,ab. (81323)
9 ((dense$ or density or high$ or large$ or heavy or heavily) adj3 (populat$ or populous) adj3 (area$ or settlement$ or district$ or neighbourhood$ or communit$)).ti,ab. (3451)
10 or/1-9 (390327)
11 (afghanistan or albania or algeria or american samoa or angola or "antigua and barbuda" or antigua or barbuda or argentina or armenia or armenian or aruba or azerbaijan or bahrain or bangladesh or barbados or republic of belarus or belarus or byelarus or belorussia or byelorussian or belize or british honduras or benin or dahomey or bhutan or bolivia or "bosnia and herzegovina" or bosnia or herzegovina or botswana or bechuanaland or brazil or brasil or bulgaria or burkina faso or burkina fasso or upper volta or burundi or urundi or cabo verde or cape verde or cambodia or kampuchea or khmer republic or cameroon or cameron or cameroun or central african republic or ubangi shari or chad or chile or china or colombia or comoros or comoro islands or iles comores or mayotte or democratic republic of the congo or democratic republic congo or congo or zaire or costa rica or "cote d’ivoire" or "cote d’ ivoire" or cote divoire or cote d ivoire or ivory coast or croatia or cuba or cyprus or czech republic or czechoslovakia or djibouti or french somaliland or dominica or dominican republic or ecuador or egypt or united arab republic or el salvador or equatorial guinea or spanish guinea or eritrea or estonia or eswatini or swaziland or ethiopia or fiji or gabon or gabonese republic or gambia or "georgia (republic)" or georgian or ghana or gold coast or gibraltar or greece or grenada or guam or guatemala or guinea or guinea bissau or guyana or british guiana or haiti or hispaniola or honduras or hungary or india or indonesia or timor or iran or iraq or isle of man or jamaica or jordan or kazakhstan or kazakh or kenya or "democratic people’s republic of korea" or republic of korea or north korea or south korea or korea or kosovo or kyrgyzstan or kirghizia or kirgizstan or kyrgyz republic or kirghiz or laos or lao pdr or "lao people's democratic republic" or latvia or lebanon or lebanese republic or lesotho or basutoland or liberia or libya or libyan arab jamahiriya or lithuania or macau or macao or "macedonia (republic)" or macedonia or madagascar or malagasy republic or malawi or nyasaland or malaysia or malay federation or malaya federation or maldives or indian ocean islands or indian ocean or mali or malta or micronesia or federated states of micronesia or kiribati or marshall islands or nauru or northern mariana islands or palau or tuvalu or mauritania or mauritius or mexico or moldova or moldovian or mongolia or montenegro or morocco or ifni or mozambique or portuguese east africa or myanmar or burma or namibia or nepal or netherlands antilles or nicaragua or niger or nigeria or oman or muscat or pakistan or panama or

papua new guinea or new guinea or paraguay or peru or philippines or philipines or phillipines or phillippines or poland or "polish people's republic" or portugal or portuguese republic or puerto rico or romania or russia or russian federation or ussr or soviet union or union of soviet socialist republics or rwanda or ruanda or samoa or pacific islands or polynesia or samoan islands or navigator island or navigator islands or "sao tome and principe" or saudi arabia or senegal or serbia or seychelles or sierra leone or slovakia or slovak republic or slovenia or melanesia or solomon island or solomon islands or norfolk island or norfolk islands or somalia or south africa or south sudan or sri lanka or ceylon or "saint kitts and nevis" or "st. kitts and nevis" or saint lucia or "st. lucia" or "saint vincent and the grenadines" or saint vincent or "st. vincent" or grenadines or sudan or suriname or surinam or dutch guiana or netherlands guiana or syria or syrian arab republic or tajikistan or tadjikistan or tadzhikistan or tadzhik or tanzania or tanganyika or thailand or siam or timor leste or east timor or togo or togolese republic or tonga or "trinidad and tobago" or trinidad or tobago or tunisia or turkey or "turkey (republic)" or turkmenistan or turkmen or uganda or ukraine or uruguay or uzbekistan or uzbek or vanuatu or new hebrides or venezuela or vietnam or viet nam or middle east or west bank or gaza or palestine or yemen or yugoslavia or zambia or zimbabwe or northern rhodesia or global south or africa south of the sahara or sub-saharan africa or subsaharan africa or africa, central or central africa or africa, northern or north africa or northern africa or magreb or maghrib or sahara or africa, southern or southern africa or africa, eastern or east africa or eastern africa or africa, western or west africa or western africa or west indies or indian ocean islands or caribbean or central america or latin america or "south and central america" or south america or asia, central or central asia or asia, northern or north asia or northern asia or asia, southeastern or southeastern asia or south eastern asia or southeast asia or south east asia or asia, western or western asia or europe, eastern or east europe or eastern europe or developing country or developing countries or developing nation? or developing population? or developing world or less developed countr* or less developed nation? or less developed population? or less developed world or lesser developed countr* or lesser developed nation? or lesser developed population? or lesser developed world or under developed countr* or under developed nation? or under developed population? or under developed world or underdeveloped countr* or underdeveloped nation? or underdeveloped population? or underdeveloped world or middle income countr* or middle income nation? or middle income population? or low income countr* or low income nation? or low income population? or lower income countr* or lower income nation? or lower income population? or underserved countr* or underserved nation? or underserved population? or underserved world or under served countr* or under served nation? or under served population? or under served world or deprived countr* or deprived nation? or deprived population? or deprived world or poor countr* or poor nation? or poor population? or poor world or poorer countr* or poorer nation? or poorer population? or poorer world or developing economy* or developing economies or less developed econom* or lesser developed econom* or under developed econom* or underdeveloped econom* or middle income econom* or low income econom* or lower income econom* or low gdp or low gnp or low gross domestic or low gross national or lower gdp or lower gnp or lower gross domestic or lower gross national or lmic or lmics or third world or lami countr* or transitional countr* or emerging economy or emerging economies or emerging nation?).ti,ab,sh,kf. (1907989)

12 (afghan or afghans or afghani or albanian? algerian? or american samoan? or angolan? or antiguan? or barbudan? or argentine? or argentinian? or argentinean? or armenian? or aruban? or azerbaijani? or bahraini? or bangladeshi? or bangalees or bajan? or belarusian? or byelorussian? or belizean? or beninese? or bhutanese or bolivian? or bosnian? or botswana or batswana or brazilian? or brasilian? or bulgarian? or burkinabe or burkinese or burundian? or cape verdean? or cabo verdean? or cambodian? or khmer or cameroonian? or central african? or chadian? or chilean? or chinese or colombian? or comorian? or congolese or costa rican? or ivorian? or croatian? or cuban? or cypriot? or czech? or djiboutian? or dominican? or ecuadorian? or egyptian? or salvadoran? or equatorial guinean? or equatoguinean? or eritrean? or estonian? or swazi? or swati? or ethiopian? or fijian or gabonese or gabonaise or gambian? or georgian? or ghanaian? or gibraltarian? or greek?

or grenadian? or guamanian? or guatemalan? or guinean? or bissau guinean? or guyanese or haitian? or honduran? or hungarian? or indian? or indonesian? or iranian? or iraqian? or iraqi? or manx or jamaican? or jordanian? or kazakhstani? or kenyan? or kirabati or kirabatian? or north korean? or korean? or kosovar? or kosovan? or kyrgyz* or lao or laotian? or latvian? or lebanese or lesothan? or lesothonian? or mosotho or basotho or liberian? or libyan? or lithuanian? or macanese or macedonian? or malagasy or madagascan? or malawian? or malaysian? or maldivian? or malian? or maltese or marshallese? or mauritanian? or mauritian? or mexican? or micronesian? or moldovan? or mongolian? or mongol or montenegrin? or moroccan? or mozambican? or burmese or myanma or namibian? or nauruan? or nepali or nepalese or netherlands antillean? or nicaraguan? or nigerien? or nigerian? or northern mariana islander? or mariana? or omani? or pakistani? or palauan? or panamanian? or papua new guinean? or paraguayan? or peruvian? or philippine? or philipine? or phillipine? or phillippine? or filipino? or filipina? or polish or pole or poles or portuguese or puerto rican? or romanian? or russian? or soviet people or soviet population or rwandan? or rwandese or ruandan? or ruandese or samoan? or sao tomean? or santomean? or saudi arabian? or saudi? or senegalese or serbian? or montenegrin? or seychellois or seychelloise? or sierra leonean? or slovak? or slovene? or solomon islander? or somali? or south african? or south sudanese or sri lankan? or ceylonese or kittitian? or nevisian? or saint lucian? or vincentian? or sudanese or surinamese? or syrian? or tajik? or tajikistani? or tanzanian? or tanganyikan? or thai or timorese? or togolese or tongan? or trinidadian? or tobagonian? or tunisian? or turk? or turkish or turkmen? or tuvaluan? or ugandan? or ukrainian? or uruguayan? or uzbek? or vanuatu* or venezuelan? or vietnamese or yemeni? or yemenite? or yemenese or yugoslav? or yugoslavian? or zambian? or zimbabwean?).ti,ab,sh,kf. (834927)
13 11 or 12 (2269396)
14 10 and 13 (169934)

COSTS
15 poverty areas/ (6047)
16 (slum or slums or ghetto or ghettos or shanty$ or shanties or shack$ or favela$).ti,ab. (5200)
17 (informal$ adj3 settlement$).ti,ab. (558)
18 ((poverty or impoverish$) adj3 (area$ or settlement$)).ti,ab. (881)
19 ((precarious$ or irregular$) adj3 settlement$).ti,ab. (8)
20 (squatter$ adj3 (area$ or settlement$)).ti,ab. (227)
21 or/15-20 (11156)
22 "cost of illness"/ (27054)
23 exp Health Care Costs/ (64997)
24 Health Expenditures/ (20173)
25 exp Financing, Personal/ (6053)
26 Catastrophic Illness/ec [Economics] (550)
27 (cost$ adj2 (illness$ or sickness$ or disease$)).ti,ab. (5084)
28 (cost$ adj3 (care or health or healthcare or medical$)).ti,ab. (76646)
29 (expenditure$ adj3 (care or health or healthcare or medical$)).ti,ab. (13957)
30 ((direct or indirect) adj2 (cost$ or expenditure$)).ti,ab. (15577)
31 out of pocket.ti,ab. (5698)
32 (OOP adj3 (cost$ or expense$ or expenditure$ or financ$ or pay$ or paid or spend$)).ti,ab. (505)
33 OOPE.ti,ab. (74)
34 ((personal or individual$ or patient$ or family$ or families or household$) adj3 (budget$ or cost$ or expense$ or expenditure$ or financ$ or pay$ or paid or spend$) adj3 (care or health or healthcare or medical$)).ti,ab. (9512)
35 (catastroph$ adj10 (cost$ or expense$ or expenditure$ or financ$ or pay$ or paid or spend$)).ti,ab. (1323)

36 ((medical$ or health) adj3 impoverish$).ti,ab. (232)
37 (burden$ adj3 (cost$ or expenditure$ or economic$ or financ$) adj3 (care or health or healthcare or medical$)).ti,ab. (4485)
38 ((willing$ or unwilling$) adj3 pay$ adj3 (care or health or healthcare or medical$)).ti,ab. (323)
39 ((afford$ or unafford$) adj5 (cost$ or pay$)).ti,ab. (2273)
40 ((able or ability or capacit$ or unable or inability) adj2 pay$).ti,ab. (1460)
41 or/22-40 (185296)
42 14 and 41 (3361)
43 21 and 41 (267)
44 42 or 43 (3541)

TYPE OF STUDY
45 letter.pt. (1084673)
46 editorial.pt. (532516)
47 historical article.pt. (358723)
48 or/45-47 (1956361)
49 44 not 48 (3506)
50 exp animals/ not humans/ (4709143)
51 49 not 50 (3497)
52 limit 51 to yr="2010 -Current" (2368)

Key:
/ = subject heading (MeSH heading)
exp = exploded subject heading (MeSH heading)
/ec = economics subheading
$ = truncation
* = truncation
“ “ = exact phrase search
? = stands for zero or one characters within a word or at the end of a word
ti,ab = terms in either title or abstract fields
kf = terms in author keywords field
sh = terms in the subject heading field
pt = terms in publication type field
adj3 = terms within three words of each other (any order)
adj = terms next to each other in the same order

Embase
via Ovid http://ovidsp.ovid.com/
1974 to 2020 June 19
Search date: 22nd June 2020
Records retrieved: 2958

1 urban area/ (60321)
2 urban population/ (44806)
3 urban health/ (1037)
4 urban hospital/ (303)
5 city/ (38692)
6 suburban area/ (2548)
7 suburban population/ (670)
8 (city or cities or megacity or megacities or metropolitan).ti,ab. (219736)

9 (metropolis or megalopolis or municipal$ or conurbation$ or suburb$ or town or towns or township$ or borough$ or barrio or barrios).ti,ab. (100040)
10 ((dense$ or density or high$ or large$ or heavy or heavily) adj3 (populat$ or populous) adj3 (area$ or settlement$ or district$ or neighbourhood$ or communit$)).ti,ab. (4243)
11 urban$.ti,ab. (200356)
12 or/1-11 (486662)
13 (afghanistan or albania or algeria or american samoa or angola or "antigua and barbuda" or antigua or barbuda or argentina or armenia or armenian or aruba or azerbaijan or bahrain or bangladesh or barbados or republic of belarus or belarus or byelarus or belorussia or byelorussian or belize or british honduras or benin or dahomey or bhutan or bolivia or "bosnia and herzegovina" or bosnia or herzegovina or botswana or bechuanaland or brazil or brasil or bulgaria or burkina faso or burkina fasso or upper volta or burundi or urundi or cabo verde or cape verde or cambodia or kampuchea or khmer republic or cameroon or cameron or cameroun or central african republic or ubangi shari or chad or chile or china or colombia or comoros or comoro islands or iles comores or mayotte or democratic republic of the congo or democratic republic congo or congo or zaire or costa rica or "cote d’ivoire" or "cote d’ ivoire" or cote divoire or cote d ivoire or ivory coast or croatia or cuba or cyprus or czech republic or czechoslovakia or djibouti or french somaliland or dominica or dominican republic or ecuador or egypt or united arab republic or el salvador or equatorial guinea or spanish guinea or eritrea or estonia or eswatini or swaziland or ethiopia or fiji or gabon or gabonese republic or gambia or "georgia (republic)" or georgian or ghana or gold coast or gibraltar or greece or grenada or guam or guatemala or guinea or guinea bissau or guyana or british guiana or haiti or hispaniola or honduras or hungary or india or indonesia or timor or iran or iraq or isle of man or jamaica or jordan or kazakhstan or kazakh or kenya or "democratic people’s republic of korea" or republic of korea or north korea or south korea or korea or kosovo or kyrgyzstan or kirghizia or kirgizstan or kyrgyz republic or kirghiz or laos or lao pdr or "lao people's democratic republic" or latvia or lebanon or lebanese republic or lesotho or basutoland or liberia or libya or libyan arab jamahiriya or lithuania or macau or macao or "macedonia (republic)" or macedonia or madagascar or malagasy republic or malawi or nyasaland or malaysia or malay federation or malaya federation or maldives or indian ocean islands or indian ocean or mali or malta or micronesia or federated states of micronesia or kiribati or marshall islands or nauru or northern mariana islands or palau or tuvalu or mauritania or mauritius or mexico or moldova or moldovian or mongolia or montenegro or "montenegro (republic)" or morocco or ifni or mozambique or portuguese east africa or myanmar or burma or namibia or nepal or netherlands antilles or nicaragua or niger or nigeria or oman or muscat or pakistan or panama or papua new guinea or new guinea or paraguay or peru or philippines or philipines or phillipines or phillippines or poland or "polish people's republic" or portugal or portuguese republic or puerto rico or romania or russia or russian federation or ussr or soviet union or union of soviet socialist republics or rwanda or ruanda or samoa or pacific islands or polynesia or samoan islands or navigator island or navigator islands or "sao tome and principe" or saudi arabia or senegal or serbia or seychelles or sierra leone or slovakia or slovak republic or slovenia or melanesia or solomon island or solomon islands or norfolk island or norfolk islands or somalia or south africa or south sudan or sri lanka or ceylon or "saint kitts and nevis" or "st. kitts and nevis" or saint lucia or "st. lucia" or "saint vincent and the grenadines" or saint vincent or "st. vincent" or grenadines or sudan or suriname or surinam or dutch guiana or netherlands guiana or syria or syrian arab republic or tajikistan or tadjikistan or tadzhikistan or tadzhik or tanzania or tanganyika or thailand or siam or timor leste or east timor or togo or togolese republic or tonga or "trinidad and tobago" or trinidad or tobago or tunisia or turkey or "turkey (republic)" or turkmenistan or turkmen or uganda or ukraine or uruguay or uzbekistan or uzbek or vanuatu or new hebrides or venezuela or vietnam or viet nam or middle east or west bank or gaza or palestine or yemen or yugoslavia or zambia or zimbabwe or northern rhodesia or global south or africa south of the sahara or "sub saharan africa" or subsaharan africa or africa, central or central africa or africa, northern or north africa or northern africa or magreb or maghrib or sahara or africa, southern or southern africa or africa, eastern or east africa or

eastern africa or africa, western or west africa or western africa or west indies or indian ocean islands or caribbean region or caribbean islands or caribbean or central america or latin america or "south and central america" or south america or asia, central or central asia or asia, northern or north asia or northern asia or asia, southeastern or southeastern asia or south eastern asia or southeast asia or south east asia or asia, western or western asia or europe, eastern or east europe or eastern europe or developing country or developing countries or developing nation? or developing population? or developing world or less developed countr* or less developed nation? or less developed population? or less developed world or lesser developed countr* or lesser developed nation? or lesser developed population? or lesser developed world or under developed countr* or under developed nation? or under developed population? or under developed world or underdeveloped countr* or underdeveloped nation? or underdeveloped population? or underdeveloped world or middle income countr* or middle income nation? or middle income population? or low income countr* or low income nation? or low income population? or lower income countr* or lower income nation? or lower income population? or underserved countr* or underserved nation? or underserved population? or underserved world or under served countr* or under served nation? or under served population? or under served world or deprived countr* or deprived nation? or deprived population? or deprived world or poor countr* or poor nation? or poor population? or poor world or poorer countr* or poorer nation? or poorer population? or poorer world or developing econom* or less developed econom* or lesser developed econom* or under developed econom* or underdeveloped econom* or middle income econom* or low income econom* or lower income econom* or low gdp or low gnp or low gross domestic or low gross national or lower gdp or lower gnp or lower gross domestic or lower gross national or lmic or lmics or third world or lami countr* or transitional countr* or emerging economies or emerging economy or emerging nation?).ti,ab,sh,kw. (2145013)

14 (afghan or afghans or afghani or albanian? algerian? or american samoan? or angolan? or antiguan? or barbudan? or argentine? or argentinian? or argentinean? or armenian? or aruban? or azerbaijani? or bahraini? or bangladeshi? or bangalees or bajan? or belarusian? or byelorussian? or belizean? or beninese? or bhutanese or bolivian? or bosnian? or botswana or batswana or brazilian? or brasilian? or bulgarian? or burkinabe or burkinese or burundian? or cape verdean? or cabo verdean? or cambodian? or khmer or cameroonian? or central african? or chadian? or chilean? or chinese or colombian? or comorian? or congolese or costa rican? or ivorian? or croatian? or cuban? or cypriot? or czech? or djiboutian? or dominican? or ecuadorian? or egyptian? or salvadoran? or equatorial guinean? or equatoguinean? or eritrean? or estonian? or swazi? or swati? or ethiopian? or fijian or gabonese or gabonaise or gambian? or georgian? or ghanaian? or gibraltarian? or greek? or grenadian? or guamanian? or guatemalan? or guinean? or bissau guinean? or guyanese or haitian? or honduran? or hungarian? or indian? or indonesian? or iranian? or iraqian? or iraqi? or manx or jamaican? or jordanian? or kazakhstani? or kenyan? or kirabati or kirabatian? or north korean? or korean? or kosovar? or kosovan? or kyrgyz* or lao or laotian? or latvian? or lebanese or lesothan? or lesothonian? or mosotho or basotho or liberian? or libyan? or lithuanian? or macanese or macedonian? or malagasy or madagascan? or malawian? or malaysian? or maldivian? or malian? or maltese or marshallese? or mauritanian? or mauritian? or mexican? or micronesian? or moldovan? or mongolian? or mongol or montenegrin? or moroccan? or mozambican? or burmese or myanma or namibian? or nauruan? or nepali or nepalese or netherlands antillean? or nicaraguan? or nigerien? or nigerian? or northern mariana islander? or mariana? or omani? or pakistani? or palauan? or panamanian? or papua new guinean? or paraguayan? or peruvian? or philippine? or philipine? or phillipine? or phillippine? or filipino? or filipina? or polish or pole or poles or portuguese or puerto rican? or romanian? or russian? or soviet people or soviet population or rwandan? or rwandese or ruandan? or ruandese or samoan? or sao tomean? or santomean? or saudi arabian? or saudi? or senegalese or serbian? or montenegrin? or seychellois or seychelloise? or sierra leonean? or slovak? or slovene? or solomon islander? or somali? or south african? or south sudanese or sri lankan? or ceylonese or kittitian? or nevisian? or saint lucian? or vincentian? or

sudanese or surinamese? or syrian? or tajik? or tajikistani? or tanzanian? or tanganyikan? or thai or timorese? or togolese or tongan? or trinidadian? or tobagonian? or tunisian? or turk? or turkish or turkmen? or tuvaluan? or ugandan? or ukrainian? or uruguayan? or uzbek? or vanuatu* or venezuelan? or vietnamese or yemeni? or yemenite? or yemenese or yugoslav? or yugoslavian? or zambian? or zimbabwean?).ti,ab,sh,kw. (1119374)
15 13 or 14 (2730608)
16 12 and 15 (203906)
17 (slum or slums or ghetto or ghettos or shanty$ or shanties or shack$ or favela$).mp. (5658)
18 (informal$ adj3 settlement$).mp. (628)
19 ((poverty or impoverish$) adj3 (area$ or settlement$)).mp. (1075)
20 ((precarious$ or irregular$) adj3 settlement$).mp. (12)
21 (squatter$ adj3 (area$ or settlement$)).mp. (207)
22 or/17-21 (7364)
23 "cost of illness"/ (19139)
24 exp "health care cost"/ (289949)
25 health economics/ (32736)
26 willingness to pay/ (695)
27 catastrophic health expenditure/ (32)
28 out of pocket/ (3)
29 (cost$ adj2 (illness$ or sickness$ or disease$)).ti,ab. (7680)
30 (cost$ adj3 (care or health or healthcare or medical$)).ti,ab. (114334)
31 (expenditure$ adj3 (care or health or healthcare or medical$)).ti,ab. (18625)
32 ((direct or indirect) adj2 (cost$ or expenditure$)).ti,ab. (26279)
33 out of pocket.ti,ab. (8054)
34 (OOP adj3 (cost$ or expense$ or expenditure$ or financ$ or pay$ or paid or spend$)).ti,ab. (797)
35 OOPE.ti,ab. (95)
36 ((personal or individual$ or patient$ or family$ or families or household$) adj3 (budget$ or cost$ or expense$ or expenditure$ or financ$ or pay$ or paid or spend$) adj3 (care or health or healthcare or medical$)).ti,ab. (14828)
37 (catastroph$ adj10 (cost$ or expense$ or expenditure$ or financ$ or pay$ or paid or spend$)).ti,ab. (1521)
38 ((medical$ or health) adj3 impoverish$).ti,ab. (241)
39 (burden$ adj3 (cost$ or expenditure$ or economic$ or financ$) adj3 (care or health or healthcare or medical$)).ti,ab. (6941)
40 ((willing$ or unwilling$) adj3 pay$ adj3 (care or health or healthcare or medical$)).ti,ab. (458)
41 ((afford$ or unafford$) adj5 (cost$ or pay$)).ti,ab. (3175)
42 ((able or ability or capacit$ or unable or inability) adj2 pay$).ti,ab. (1801)
43 or/23-42 (414755)
44 16 and 43 (5138)
45 22 and 43 (202)
46 44 or 45 (5237)
47 letter.pt. (1118273)
48 editorial.pt. (655452)
49 47 or 48 (1773725)
50 46 not 49 (5179)
51 limit 50 to conference abstracts (970)
52 50 not 51 (4209)
53 limit 52 to yr="2010 -Current" (2958)

Key:

/ = subject heading (EMTREE term)
exp = exploded subject heading (EMTREE term)
$ = truncation
* = truncation
" " = exact phrase search
? = stands for zero or one characters within a word or at the end of a word
ti,ab = terms in either title or abstract fields
mp = multi-purpose fields search – includes title, abstract, author keywords, or subject headings field
kw = terms in author keywords field
sh = terms in the subject heading field
pt = terms in publication type field
adj3 = terms within three words of each other (any order)
adj = terms next to each other in the same order

EconLit
via Ovid http://ovidsp.ovid.com/
1886 to June 11 2020
Search date: 22nd June 2020
Records retrieved: 197

1 (city or cities or megacity or megacities or metropolitan).ti,ab. (35238)
2 (metropolis or megalopolis or municipal$ or conurbation$ or suburb$ or town or towns or township$ or borough$ or barrio or barrios).ti,ab. (15322)
3 ((dense$ or density or high$ or large$ or heavy or heavily) adj3 (populat$ or populous) adj3 (area$ or settlement$ or district$ or neighbourhood$ or communit$)).ti,ab. (356)
4 urban$.ti,ab. (36202)
5 or/1-4 (69848)
6 (afghanistan or albania or algeria or american samoa or angola or "antigua and barbuda" or antigua or barbuda or argentina or armenia or armenian or aruba or azerbaijan or bahrain or bangladesh or barbados or republic of belarus or belarus or byelarus or belorussia or byelorussian or belize or british honduras or benin or dahomey or bhutan or bolivia or "bosnia and herzegovina" or bosnia or herzegovina or botswana or bechuanaland or brazil or brasil or bulgaria or burkina faso or burkina fasso or upper volta or burundi or urundi or cabo verde or cape verde or cambodia or kampuchea or khmer republic or cameroon or cameron or cameroun or central african republic or ubangi shari or chad or chile or china or colombia or comoros or comoro islands or iles comores or mayotte or democratic republic of the congo or democratic republic congo or congo or zaire or costa rica or "cote d'ivoire" or "cote d' ivoire" or cote divoire or cote d ivoire or ivory coast or croatia or cuba or cyprus or czech republic or czechoslovakia or djibouti or french somaliland or dominica or dominican republic or ecuador or egypt or united arab republic or el salvador or equatorial guinea or spanish guinea or eritrea or estonia or eswatini or swaziland or ethiopia or fiji or gabon or gabonese republic or gambia or "georgia (republic)" or georgian or ghana or gold coast or gibraltar or greece or grenada or guam or guatemala or guinea or guinea bissau or guyana or british guiana or haiti or hispaniola or honduras or hungary or india or indonesia or timor or iran or iraq or isle of man or jamaica or jordan or kazakhstan or kazakh or kenya or "democratic people's republic of korea" or republic of korea or north korea or south korea or korea or kosovo or kyrgyzstan or kirghizia or kirgizstan or kyrgyz republic or kirghiz or laos or lao pdr or "lao people's democratic republic" or latvia or lebanon or lebanese republic or lesotho or basutoland or liberia or libya or libyan arab jamahiriya or lithuania or macau or macao or "macedonia (republic)" or macedonia or madagascar or malagasy republic or malawi or nyasaland or malaysia or malay federation or malaya federation or maldives or indian ocean islands or indian ocean or mali or malta or micronesia or federated

states of micronesia or kiribati or marshall islands or nauru or northern mariana islands or palau or tuvalu or mauritania or mauritius or mexico or moldova or moldovian or mongolia or montenegro or morocco or ifni or mozambique or portuguese east africa or myanmar or burma or namibia or nepal or netherlands antilles or nicaragua or niger or nigeria or oman or muscat or pakistan or panama or papua new guinea or new guinea or paraguay or peru or philippines or philipines or phillipines or phillippines or poland or "polish people's republic" or portugal or portuguese republic or puerto rico or romania or russia or russian federation or ussr or soviet union or union of soviet socialist republics or rwanda or ruanda or samoa or pacific islands or polynesia or samoan islands or navigator island or navigator islands or "sao tome and principe" or saudi arabia or senegal or serbia or seychelles or sierra leone or slovakia or slovak republic or slovenia or melanesia or solomon island or solomon islands or norfolk island or norfolk islands or somalia or south africa or south sudan or sri lanka or ceylon or "saint kitts and nevis" or "st. kitts and nevis" or saint lucia or "st. lucia" or "saint vincent and the grenadines" or saint vincent or "st. vincent" or grenadines or sudan or suriname or surinam or dutch guiana or netherlands guiana or syria or syrian arab republic or tajikistan or tadjikistan or tadzhikistan or tadzhik or tanzania or tanganyika or thailand or siam or timor leste or east timor or togo or togolese republic or tonga or "trinidad and tobago" or trinidad or tobago or tunisia or turkey or "turkey (republic)" or turkmenistan or turkmen or uganda or ukraine or uruguay or uzbekistan or uzbek or vanuatu or new hebrides or venezuela or vietnam or viet nam or middle east or west bank or gaza or palestine or yemen or yugoslavia or zambia or zimbabwe or northern rhodesia or global south or africa south of the sahara or sub-saharan africa or subsaharan africa or africa, central or central africa or africa, northern or north africa or northern africa or magreb or maghrib or sahara or africa, southern or southern africa or africa, eastern or east africa or eastern africa or africa, western or west africa or western africa or west indies or indian ocean islands or caribbean or central america or latin america or "south and central america" or south america or asia, central or central asia or asia, northern or north asia or northern asia or asia, southeastern or southeastern asia or south eastern asia or southeast asia or south east asia or asia, western or western asia or europe, eastern or east europe or eastern europe or developing country or developing countries or developing nation? or developing population? or developing world or less developed countr* or less developed nation? or less developed population? or less developed world or lesser developed countr* or lesser developed nation? or lesser developed population? or lesser developed world or under developed countr* or under developed nation? or under developed population? or under developed world or underdeveloped countr* or underdeveloped nation? or underdeveloped population? or underdeveloped world or middle income countr* or middle income nation? or middle income population? or low income countr* or low income nation? or low income population? or lower income countr* or lower income nation? or lower income population? or underserved countr* or underserved nation? or underserved population? or underserved world or under served countr* or under served nation? or under served population? or under served world or deprived countr* or deprived nation? or deprived population? or deprived world or poor countr* or poor nation? or poor population? or poor world or poorer countr* or poorer nation? or poorer population? or poorer world or developing econom* or less developed econom* or lesser developed econom* or under developed econom* or underdeveloped econom* or middle income econom* or low income econom* or lower income econom* or low gdp or low gnp or low gross domestic or low gross national or lower gdp or lower gnp or lower gross domestic or lower gross national or lmic or lmics or third world or lami countr* or transitional countr* or emerging economies or emerging economy or emerging nation?).ti,ab,sh,kw. (270638)

7 (afghan or afghans or afghani or albanian? algerian? or american samoan? or angolan? or antiguan? or barbudan? or argentine? or argentinian? or argentinean? or armenian? or aruban? or azerbaijani? or bahraini? or bangladeshi? or bangalees or bajan? or belarusian? or byelorussian? or belizean? or beninese? or bhutanese or bolivian? or bosnian? or botswana or batswana or brazilian? or brasilian? or bulgarian? or burkinabe or burkinese or burundian? or cape verdean? or cabo verdean? or cambodian? or khmer or cameroonian? or central african? or chadian? or chilean? or

chinese or colombian? or comorian? or congolese or costa rican? or ivorian? or croatian? or cuban? or cypriot? or czech? or djiboutian? or dominican? or ecuadorian? or egyptian? or salvadoran? or equatorial guinean? or equatoguinean? or eritrean? or estonian? or swazi? or swati? or ethiopian? or fijian or gabonese or gabonaise or gambian? or georgian? or ghanaian? or gibraltarian? or greek? or grenadian? or guamanian? or guatemalan? or guinean? or bissau guinean? or guyanese or haitian? or honduran? or hungarian? or indian? or indonesian? or iranian? or iraqian? or iraqi? or manx or jamaican? or jordanian? or kazakhstani? or kenyan? or kirabati or kirabatian? or north korean? or korean? or kosovar? or kosovan? or kyrgyz* or lao or laotian? or latvian? or lebanese or lesothan? or lesothonian? or mosotho or basotho or liberian? or libyan? or lithuanian? or macanese or macedonian? or malagasy or madagascan? or malawian? or malaysian? or maldivian? or malian? or maltese or marshallese? or mauritanian? or mauritian? or mexican? or micronesian? or moldovan? or mongolian? or mongol or montenegrin? or moroccan? or mozambican? or burmese or myanma or namibian? or nauruan? or nepali or nepalese or netherlands antillean? or nicaraguan? or nigerien? or nigerian? or northern mariana islander? or mariana? or omani? or pakistani? or palauan? or panamanian? or papua new guinean? or paraguayan? or peruvian? or philippine? or philipine? or phillipine? or phillippine? or filipino? or filipina? or polish or pole or poles or portuguese or puerto rican? or romanian? or russian? or soviet people or soviet population or rwandan? or rwandese or ruandan? or ruandese or samoan? or sao tomean? or santomean? or saudi arabian? or saudi? or senegalese or serbian? or montenegrin? or seychellois or seychelloise? or sierra leonean? or slovak? or slovene? or solomon islander? or somali? or south african? or south sudanese or sri lankan? or ceylonese or kittitian? or nevisian? or saint lucian? or vincentian? or sudanese or surinamese? or syrian? or tajik? or tajikistani? or tanzanian? or tanganyikan? or thai or timorese? or togolese or tongan? or trinidadian? or tobagonian? or tunisian? or turk? or turkish or turkmen? or tuvaluan? or ugandan? or ukrainian? or uruguayan? or uzbek? or vanuatu* or venezuelan? or vietnamese or yemeni? or yemenite? or yemenese or yugoslav? or yugoslavian? or zambian? or zimbabwean?).ti,ab,sh,kw. (120512)

8 6 or 7 (320416)

9 5 and 8 (23130)

10 (slum or slums or ghetto or ghettos or shanty$ or shanties or shack$ or favela$).ti,ab. (1156)

11 (informal$ adj3 settlement$).ti,ab. (206)

12 ((poverty or impoverish$) adj3 (area$ or settlement$)).ti,ab. (379)

13 ((precarious$ or irregular$) adj3 settlement$).ti,ab. (11)

14 (squatter$ adj3 (area$ or settlement$)).ti,ab. (70)

15 or/10-14 (1743)

16 (cost$ adj2 (illness$ or sickness$ or disease$)).ti,ab. (415)

17 (cost$ adj3 (care or health or healthcare or medical$)).ti,ab. (3305)

18 (expenditure$ adj3 (care or health or healthcare or medical$)).ti,ab. (2943)

19 ((direct or indirect) adj2 (cost$ or expenditure$)).ti,ab. (1415)

20 out of pocket.ti,ab. (868)

21 (OOP adj3 (cost$ or expense$ or expenditure$ or financ$ or pay$ or paid or spend$)).ti,ab. (71)

22 OOPE.ti,ab. (4)

23 ((personal or individual$ or patient$ or family$ or families or household$) adj3 (budget$ or cost$ or expense$ or expenditure$ or financ$ or pay$ or paid or spend$) adj3 (care or health or healthcare or medical$)).ti,ab. (599)

24 (catastroph$ adj10 (cost$ or expense$ or expenditure$ or financ$ or pay$ or paid or spend$)).ti,ab. (449)

25 ((medical$ or health) adj3 impoverish$).ti,ab. (23)

26 (burden$ adj3 (cost$ or expenditure$ or economic$ or financ$) adj3 (care or health or healthcare or medical$)).ti,ab. (106)

27 ((willing$ or unwilling$) adj3 pay$ adj3 (care or health or healthcare or medical$)).ti,ab. (127)

28 ((afford$ or unafford$) adj5 (cost$ or pay$)).ti,ab. (315)

29 ((able or ability or capacit$ or unable or inability) adj2 pay$).ti,ab. (717)
30 or/16-29 (9428)
31 9 and 30 (292)
32 15 and 30 (18)
33 31 or 32 (302)
34 limit 33 to yr="2010 -Current" (197)

Key:
$ = truncation
* = truncation
“ “ = exact phrase search
? = stands for zero or one characters within a word or at the end of a word
ti,ab = terms in either title or abstract fields
kw = terms in keywords field
sh = terms in the subject heading field
adj3 = terms within three words of each other (any order)
adj = terms next to each other in the same order

Science Citation Index
via Web of Science, Clarivate Analytics https://clarivate.com/
1900 – 19th June 2020
Searched on: 22nd June 2020
Records retrieved: 1538

# 35 1,538 #32 not #33
Indexes=SCI-EXPANDED Timespan=2010-2020
# 34 1,922 #32 not #33
# 33 1,028,173 CF=conference
# 32 1,929 #31 OR #30
# 31 99 #29 AND #14
# 30 1,888 #29 AND #8
# 29 102,229 #28 OR #27 OR #26 OR #25 OR #24 OR #23 OR #22 OR #21 OR #20 OR #19 OR #18 OR #17 OR #16 OR #15
# 28 1,622 TS=((able or ability or capacit* or unable or inability) NEAR/2 pay*)
# 27 3,128 TS=((afford* or unafford*) NEAR/5 (cost* or pay*))
# 26 379 TS=((willing* or unwilling*) NEAR/3 pay* NEAR/3 (care or health or healthcare or medical*))
# 25 4,603 TS=(burden* NEAR/3 (cost* or expenditure* or economic* or financ*) NEAR/3 (care or health or healthcare or medical*))
# 24 207 TS=((medical* or health) NEAR/3 impoverish*)
# 23 1,220 TS=(catastroph* NEAR/10 (cost* or expense* or expenditure* or financ* or pay* or paid or spend*))
# 22 11,648 TS=((personal or individual* or patient* or family* or families or household*) NEAR/3 (budget* or cost* or expense* or expenditure* or financ* or pay* or paid or spend*) NEAR/3 (care or health or healthcare or medical*))
# 21 46 TS=OOPE
# 20 368 TS=(OOP NEAR/3 (cost* or expense* or expenditure* or financ* or pay* or paid or spend*))
# 19 4,311 TS="out of pocket"
# 18 16,894 TS=((direct or indirect) NEAR/2 (cost* or expenditure*))
# 17 11,186 TS=(expenditure* NEAR/3 (care or health or healthcare or medical*))

16 68,145 TS=(cost* NEAR/3 (care or health or healthcare or medical*))
15 8,010 TS=(cost* NEAR/2 (illness* or sickness* or disease*))
14 7,749 #13 OR #12 OR #11 OR #10 OR #9
13 99 TS=(squatter* NEAR/3 (area* or settlement*))
12 37 TS=((precarious* or irregular*) NEAR/3 settlement*)
11 1,094 TS=((poverty or impoverish*) NEAR/3 (area* or settlement*))
10 791 TS=(informal NEAR/3 settlement*)
9 6,013 TS=(slum or slums or ghetto or ghettos or shanty* or shanties or shack* or favela*)
8 186,228 #7 AND #4
7 2,792,724 #6 OR #5
6 589,412 TS=(afghan or afghans or afghani or albanian? algerian? or american samoan? or angolan? or antiguan? or barbudan? or argentine? or argentinian? or argentinean? or armenian? or aruban? or azerbaijani? or bahraini? or bangladeshi? or bangalees or bajan? or belarusian? or byelorussian? or belizean? or beninese? or bhutanese or bolivian? or bosnian? or botswana or batswana or brazilian? or brasilian? or bulgarian? or burkinabe or burkinese or burundian? or cape verdean? or cabo verdean? or cambodian? or khmer or cameroonian? or central african? or chadian? or chilean? or chinese or colombian? or comorian? or congolese or costa rican? or ivorian? or croatian? or cuban? or cypriot? or czech? or djiboutian? or dominican? or ecuadorian? or egyptian? or salvadoran? or equatorial guinean? or equatoguinean? or eritrean? or estonian? or swazi? or swati? or ethiopian? or fijian or gabonese or gabonaise or gambian? or georgian? or ghanaian? or gibraltarian? or greek? or grenadian? or guamanian? or guatemalan? or guinean? or bissau guinean? or guyanese or haitian? or honduran? or hungarian? or indian? or indonesian? or iranian? or iraqian? or iraqi? or manx or jamaican? or jordanian? or kazakhstani? or kenyan? or kirabati or kirabatian? or north korean? or korean? or kosovar? or kosovan? or kyrgyz* or lao or laotian? or latvian? or lebanese or lesothan? or lesothonian? or mosotho or basotho or liberian? or libyan? or lithuanian? or macanese or macedonian? or malagasy or madagascan? or malawian? or malaysian? or maldivian? or malian? or maltese or marshallese? or mauritanian? or mauritian? or mexican? or micronesian? or moldovan? or mongolian? or mongol or montenegrin? or moroccan? or mozambican? or burmese or myanma or namibian? or nauruan? or nepali or nepalese or netherlands antillean? or nicaraguan? or nigerien? or nigerian? or northern mariana islander? or mariana? or omani? or pakistani? or palauan? or panamanian? or papua new guinean? or paraguayan? or peruvian? or philippine? or philipine? or phillipine? or phillippine? or filipino? or filipina? or polish or pole or poles or portuguese or puerto rican? or romanian? or russian? or soviet people or soviet population or rwandan? or rwandese or ruandan? or ruandese or samoan? or sao tomean? or santomean? or saudi arabian? or saudi? or senegalese or serbian? or montenegrin? or seychellois or seychelloise? or sierra leonean? or slovak? or slovene? or solomon islander? or somali? or south african? or south sudanese or sri lankan? or ceylonese or kittitian? or nevisian? or saint lucian? or vincentian? or sudanese or surinamese? or syrian? or tajik? or tajikistani? or tanzanian? or tanganyikan? or thai or timorese? or togolese or tongan? or trinidadian? or tobagonian? or tunisian? or turk? or turkish or turkmen? or tuvaluan? or ugandan? or ukrainian? or uruguayan? or uzbek? or vanuatu* or venezuelan? or vietnamese or yemeni? or yemenite? or yemenese or yugoslav? or yugoslavian? or zambian? or zimbabwean?)
5 2,363,830 TS=(afghanistan or albania or algeria or "american samoa" or angola or "antigua and barbuda" or antigua or barbuda or argentina or armenia or armenian or aruba or azerbaijan or bahrain or bangladesh or barbados or "republic of belarus" or belarus or byelarus or belorussia or byelorussian or belize or "british honduras" or benin or dahomey or bhutan or bolivia or "bosnia and herzegovina" or bosnia or herzegovina or botswana or

bechuanaland or brazil or brasil or bulgaria or "burkina faso" or "burkina fasso" or "upper volta" or burundi or urundi or "cabo verde" or "cape verde" or cambodia or kampuchea or "khmer republic" or cameroon or cameron or cameroun or "central african republic" or "ubangi shari" or chad or chile or china or colombia or comoros or "comoro islands" or "iles comores" or mayotte or "democratic republic of the congo" or "democratic republic congo" or congo or zaire or "costa rica" or "cote d'ivoire" or "cote d' ivoire" or "cote divoire" or "cote d ivoire" or "ivory coast" or croatia or cuba or cyprus or "czech republic" or czechoslovakia or djibouti or "french somaliland" or dominica or "dominican republic" or ecuador or egypt or "united arab republic" or "el salvador" or "equatorial guinea" or "spanish guinea" or eritrea or estonia or eswatini or swaziland or ethiopia or fiji or gabon or "gabonese republic" or gambia or "georgia (republic) " or georgian or ghana or "gold coast" or gibraltar or greece or grenada or guam or guatemala or guinea or "guinea bissau" or guyana or "british guiana" or haiti or hispaniola or honduras or hungary or india or indonesia or timor or iran or iraq or "isle of man" or jamaica or jordan or kazakhstan or kazakh or kenya or "democratic people's republic of korea" or "republic of korea" or "north korea" or "south korea" or korea or kosovo or kyrgyzstan or kirghizia or kirgizstan or "kyrgyz republic" or kirghiz or laos or "lao pdr" or "lao people's democratic republic" or latvia or lebanon or "lebanese republic" or lesotho or basutoland or liberia or libya or "libyan arab jamahiriya" or lithuania or macau or macao or "macedonia (republic) " or macedonia or madagascar or "malagasy republic" or malawi or nyasaland or malaysia or "malay federation" or "malaya federation" or maldives or "indian ocean islands" or "indian ocean" or mali or malta or micronesia or "federated states of micronesia" or kiribati or "marshall islands" or nauru or "northern mariana islands" or palau or tuvalu or mauritania or mauritius or mexico or moldova or moldovian or mongolia or montenegro or morocco or ifni or mozambique or "portuguese east africa" or myanmar or burma or namibia or nepal or "netherlands antilles" or nicaragua or niger or nigeria or oman or muscat or pakistan or panama or "papua new guinea" or "new guinea" or paraguay or peru or philippines or philipines or phillipines or phillippines or poland or "polish people's republic" or portugal or "portuguese republic" or "puerto rico" or romania or russia or "russian federation" or ussr or "soviet union" or "union of soviet socialist republics" or rwanda or ruanda or samoa or "pacific islands" or polynesia or "samoan islands" or "navigator island" or "navigator islands" or "sao tome and principe" or "saudi arabia" or senegal or serbia or seychelles or "sierra leon"e or slovakia or "slovak republic" or slovenia or melanesia or "solomon island" or "solomon islands" or "norfolk island" or "norfolk islands" or somalia or "south africa" or "south sudan" or "sri lanka" or ceylon or "saint kitts and nevis" or "st. kitts and nevis" or "saint lucia" or "st. lucia" or "saint vincent and the grenadines" or "saint vincent" or "st. vincent" or grenadines or sudan or suriname or surinam or "dutch guiana" or "netherlands guiana" or syria or "syrian arab republic" or tajikistan or tadjikistan or tadzhikistan or tadzhik or tanzania or tanganyika or thailand or siam or "timor leste" or "east timor" or togo or "togolese republic" or tonga or "trinidad and tobago" or trinidad or tobago or tunisia or turkey or "turkey (republic) " or turkmenistan or turkmen or uganda or ukraine or uruguay or uzbekistan or uzbek or vanuatu or "new hebrides" or venezuela or vietnam or "viet nam" or "middle east" or "west bank" or gaza or palestine or yemen or yugoslavia or zambia or zimbabwe or "northern rhodesia" or "global south" or "africa south of the sahara" or "sub-saharan africa" or "subsaharan africa" or "africa, central" or "central africa" or "africa, northern" or "north africa" or "northern africa" or magreb or maghrib or sahara or "africa, southern" or "southern africa" or "africa, eastern" or "east africa" or "eastern africa" or "africa, western" or "west africa" or "western africa" or "west indies" or "indian ocean islands" or caribbean or "central america" or "latin america" or "south and central america" or "south america" or "asia, central" or "central asia" or "asia, northern" or "north asia" or "northern asia" or "asia, southeastern" or "southeastern asia" or "south eastern asia" or "southeast asia" or "south east asia" or "asia,

western" or "western asia" or "europe, eastern" or "east europe" or "eastern europe" or "developing country" or "developing countries" or "developing nation" or "developing nations" or "developing population" or "developing populations" or "developing world" or "less developed country" or "less developed countries" or "less developed nation" or "less developed nations" or "less developed population" or "less developed populations" or "less developed world" or "lesser developed country" or "lesser developed countries" or "lesser developed nation" or "lesser developed nations" or "lesser developed population" or "lesser developed populations" or "lesser developed world" or "under developed country" or "under developed countries" or "under developed nation" or "under developed nations" or "under developed population" or "under developed populations" or "under developed world" or "underdeveloped country" or "underdeveloped countries" or "underdeveloped nation" or "underdeveloped nations" or "underdeveloped population" or "underdeveloped populations" or "underdeveloped world" or "middle income country" or "middle income countries" or "middle income nation" or "middle income nations" or "middle income population" or "middle income populations" or "low income country" or "low income countries" or "low income nation" or "low income nations" or "low income population" or "low income populations" or "lower income country" or "lower income countries" or "lower income nation" or "lower income nations" or "lower income population" or "lower income populations" or "underserved country" or "underserved countries" or "underserved nation" or "underserved nations" or "underserved population" or "underserved populations" or "underserved world" or "under served country" or "under served countries" or "under served nation" or "under served nations" or "under served population" or "under served populations" or "under served world" or "deprived country" or "deprived countries" or "deprived nation" or "deprived nations" or "deprived population" or "deprived populations" or "deprived world" or "poor country" or "poor countries" or "poor nation" or "poor nations" or "poor population" or "poor populations" or "poor world" or "poorer country" or "poorer countries" or "poorer nation" or "poorer nations" or "poorer population" or "poorer populations" or "poorer world" or "developing economy" or "developing economies" or "less developed economy" or "less developed economies" or "lesser developed economy" or "lesser developed economies" or "under developed economy" or "under developed economies" or "underdeveloped economy" or "underdeveloped economies" or "middle income economy" or "middle income economies" or "low income economy" or "low income economies" or "lower income economy" or "lower income economies" or "low gdp" or "low gnp" or "low gross domestic" or "low gross national" or "lower gdp" or "lower gnp" or "lower gross domestic" or "lower gross national" or lmic or lmics or "third world" or "lami country" or "lami countries" or "transitional country" or "transitional countries" or "emerging economies" or "emerging economy" or "emerging nation" or "emerging nations")

4 517,582 #3 OR #2 OR #1

3 7,570 TS=((dense* or density or high* or large* or heavy or heavily) NEAR/3 (populat* or populous) NEAR/3 (area* or settlement* or district* or neighbourhood* or communit*))

2 121,532 TS=(metropolis or megalopolis or municipal* or conurbation* or suburb* or town or towns or township* or borough* or barrio or barrios)

1 422,185 TS=(urban* or city or cities or megacity or megacities or metropolitan)

Key:

* = truncation

" " = exact phrase search

? = stands for zero or one character

TS = topic search - terms in title, abstract, keyword fields

NEAR/3 = terms within three words of each other (any order)

Social Science Citation Index
via Web of Science, Clarivate Analytics https://clarivate.com/
1956 – 19th June 2020
Searched on: 22nd June 2020
Records retrieved: 1226

# 35 1,226 #32 not #33
Indexes=SSCI Timespan=2010-2020
# 34 1,545 #32 not #33
# 33 58,510 CF=conference
# 32 1,557 #31 OR #30
# 31 87 #29 AND #14
# 30 1,527 #29 AND #8
# 29 50,953 #28 OR #27 OR #26 OR #25 OR #24 OR #23 OR #22 OR #21 OR #20 OR #19 OR #18 OR #17 OR #16 OR #15
# 28 1,469 TS=((able or ability or capacit* or unable or inability) NEAR/2 pay*)
# 27 1,102 TS=((afford* or unafford*) NEAR/5 (cost* or pay*))
# 26 364 TS=((willing* or unwilling*) NEAR/3 pay* NEAR/3 (care or health or healthcare or medical*))
# 25 1,709 TS=(burden* NEAR/3 (cost* or expenditure* or economic* or financ*) NEAR/3 (care or health or healthcare or medical*))
# 24 235 TS=((medical* or health) NEAR/3 impoverish*)
# 23 1,100 TS=(catastroph* NEAR/10 (cost* or expense* or expenditure* or financ* or pay* or paid or spend*))
# 22 6,230 TS=((personal or individual* or patient* or family* or families or household*) NEAR/3 (budget* or cost* or expense* or expenditure* or financ* or pay* or paid or spend*) NEAR/3 (care or health or healthcare or medical*))
# 21 41 TS=OOPE
# 20 358 TS=(OOP NEAR/3 (cost* or expense* or expenditure* or financ* or pay* or paid or spend*))
# 19 3,675 TS="out of pocket"
# 18 6,153 TS=((direct or indirect) NEAR/2 (cost* or expenditure*))
# 17 9,050 TS=(expenditure* NEAR/3 (care or health or healthcare or medical*))
# 16 32,024 TS=(cost* NEAR/3 (care or health or healthcare or medical*))
# 15 3,308 TS=(cost* NEAR/2 (illness* or sickness* or disease*))
# 14 8,916 #13 OR #12 OR #11 OR #10 OR #9
# 13 357 TS=(squatter* NEAR/3 (area* or settlement*))
# 12 44 TS=((precarious* or irregular*) NEAR/3 settlement*)
# 11 1,297 TS=((poverty or impoverish*) NEAR/3 (area* or settlement*))
# 10 1,304 TS=(informal NEAR/3 settlement*)
# 9 6,468 TS=(slum or slums or ghetto or ghettos or shanty* or shanties or shack* or favela*)
# 8 103,299 #7 AND #4
# 7 984,954 #6 OR #5
# 6 197,784 TS=(afghan or afghans or afghani or albanian? algerian? or american samoan? or angolan? or antiguan? or barbudan? or argentine? or argentinian? or argentinean? or armenian? or aruban? or azerbaijani? or bahraini? or bangladeshi? or bangalees or bajan? or belarusian? or byelorussian? or belizean? or beninese? or bhutanese or bolivian? or bosnian? or botswana or batswana or brazilian? or brasilian? or bulgarian? or burkinabe or burkinese or burundian? or cape verdean? or cabo verdean? or cambodian? or

khmer or cameroonian? or central african? or chadian? or chilean? or chinese or colombian? or comorian? or congolese or costa rican? or ivorian? or croatian? or cuban? or cypriot? or czech? or djiboutian? or dominican? or ecuadorian? or egyptian? or salvadoran? or equatorial guinean? or equatoguinean? or eritrean? or estonian? or swazi? or swati? or ethiopian? or fijian or gabonese or gabonaise or gambian? or georgian? or ghanaian? or gibraltarian? or greek? or grenadian? or guamanian? or guatemalan? or guinean? or bissau guinean? or guyanese or haitian? or honduran? or hungarian? or indian? or indonesian? or iranian? or iraqian? or iraqi? or manx or jamaican? or jordanian? or kazakhstani? or kenyan? or kirabati or kirabatian? or north korean? or korean? or kosovar? or kosovan? or kyrgyz* or lao or laotian? or latvian? or lebanese or lesothan? or lesothonian? or mosotho or basotho or liberian? or libyan? or lithuanian? or macanese or macedonian? or malagasy or madagascan? or malawian? or malaysian? or maldivian? or malian? or maltese or marshallese? or mauritanian? or mauritian? or mexican? or micronesian? or moldovan? or mongolian? or mongol or montenegrin? or moroccan? or mozambican? or burmese or myanma or namibian? or nauruan? or nepali or nepalese or netherlands antillean? or nicaraguan? or nigerien? or nigerian? or northern mariana islander? or mariana? or omani? or pakistani? or palauan? or panamanian? or papua new guinean? or paraguayan? or peruvian? or philippine? or philipine? or phillipine? or phillippine? or filipino? or filipina? or polish or pole or poles or portuguese or puerto rican? or romanian? or russian? or soviet people or soviet population or rwandan? or rwandese or ruandan? or ruandese or samoan? or sao tomean? or santomean? or saudi arabian? or saudi? or senegalese or serbian? or montenegrin? or seychellois or seychelloise? or sierra leonean? or slovak? or slovene? or solomon islander? or somali? or south african? or south sudanese or sri lankan? or ceylonese or kittitian? or nevisian? or saint lucian? or vincentian? or sudanese or surinamese? or syrian? or tajik? or tajikistani? or tanzanian? or tanganyikan? or thai or timorese? or togolese or tongan? or trinidadian? or tobagonian? or tunisian? or turk? or turkish or turkmen? or tuvaluan? or ugandan? or ukrainian? or uruguayan? or uzbek? or vanuatu* or venezuelan? or vietnamese or yemeni? or yemenite? or yemenese or yugoslav? or yugoslavian? or zambian? or zimbabwean?)

5 882,717 TS=(afghanistan or albania or algeria or "american samoa" or angola or "antigua and barbuda" or antigua or barbuda or argentina or armenia or armenian or aruba or azerbaijan or bahrain or bangladesh or barbados or "republic of belarus" or belarus or byelarus or belorussia or byelorussian or belize or "british honduras" or benin or dahomey or bhutan or bolivia or "bosnia and herzegovina" or bosnia or herzegovina or botswana or bechuanaland or brazil or brasil or bulgaria or "burkina faso" or "burkina fasso" or "upper volta" or burundi or urundi or "cabo verde" or "cape verde" or cambodia or kampuchea or "khmer republic" or cameroon or cameron or cameroun or "central african republic" or "ubangi shari" or chad or chile or china or colombia or comoros or "comoro islands" or "iles comores" or mayotte or "democratic republic of the congo" or "democratic republic congo" or congo or zaire or "costa rica" or "cote d’ivoire" or "cote d’ ivoire" or "cote divoire" or "cote d ivoire" or "ivory coast" or croatia or cuba or cyprus or "czech republic" or czechoslovakia or djibouti or "french somaliland" or dominica or "dominican republic" or ecuador or egypt or "united arab republic" or "el salvador" or "equatorial guinea" or "spanish guinea" or eritrea or estonia or eswatini or swaziland or ethiopia or fiji or gabon or "gabonese republic" or gambia or "georgia (republic) " or georgian or ghana or "gold coast" or gibraltar or greece or grenada or guam or guatemala or guinea or "guinea bissau" or guyana or "british guiana" or haiti or hispaniola or honduras or hungary or india or indonesia or timor or iran or iraq or "isle of man" or jamaica or jordan or kazakhstan or kazakh or kenya or "democratic people’s republic of korea" or "republic of korea" or "north korea" or "south korea" or korea or kosovo or kyrgyzstan or kirghizia or kirgizstan or "kyrgyz republic" or kirghiz or laos or "lao pdr" or "lao people's democratic republic" or latvia or lebanon or

"lebanese republic" or lesotho or basutoland or liberia or libya or "libyan arab jamahiriya" or lithuania or macau or macao or "macedonia (republic) " or macedonia or madagascar or "malagasy republic" or malawi or nyasaland or malaysia or "malay federation" or "malaya federation" or maldives or "indian ocean islands" or "indian ocean" or mali or malta or micronesia or "federated states of micronesia" or kiribati or "marshall islands" or nauru or "northern mariana islands" or palau or tuvalu or mauritania or mauritius or mexico or moldova or moldovian or mongolia or montenegro or morocco or ifni or mozambique or "portuguese east africa" or myanmar or burma or namibia or nepal or "netherlands antilles" or nicaragua or niger or nigeria or oman or muscat or pakistan or panama or "papua new guinea" or "new guinea" or paraguay or peru or philippines or philipines or phillipines or phillippines or poland or "polish people's republic" or portugal or "portuguese republic" or "puerto rico" or romania or russia or "russian federation" or ussr or "soviet union" or "union of soviet socialist republics" or rwanda or ruanda or samoa or "pacific islands" or polynesia or "samoan islands" or "navigator island" or "navigator islands" or "sao tome and principe" or "saudi arabia" or senegal or serbia or seychelles or "sierra leon"e or slovakia or "slovak republic" or slovenia or melanesia or "solomon island" or "solomon islands" or "norfolk island" or "norfolk islands" or somalia or "south africa" or "south sudan" or "sri lanka" or ceylon or "saint kitts and nevis" or "st. kitts and nevis" or "saint lucia" or "st. lucia" or "saint vincent and the grenadines" or "saint vincent" or "st. vincent" or grenadines or sudan or suriname or surinam or "dutch guiana" or "netherlands guiana" or syria or "syrian arab republic" or tajikistan or tadjikistan or tadzhikistan or tadzhik or tanzania or tanganyika or thailand or siam or "timor leste" or "east timor" or togo or "togolese republic" or tonga or "trinidad and tobago" or trinidad or tobago or tunisia or turkey or "turkey (republic) " or turkmenistan or turkmen or uganda or ukraine or uruguay or uzbekistan or uzbek or vanuatu or "new hebrides" or venezuela or vietnam or "viet nam" or "middle east" or "west bank" or gaza or palestine or yemen or yugoslavia or zambia or zimbabwe or "northern rhodesia" or "global south" or "africa south of the sahara" or "sub-saharan africa" or "subsaharan africa" or "africa, central" or "central africa" or "africa, northern" or "north africa" or "northern africa" or magreb or maghrib or sahara or "africa, southern" or "southern africa" or "africa, eastern" or "east africa" or "eastern africa" or "africa, western" or "west africa" or "western africa" or "west indies" or "indian ocean islands" or caribbean or "central america" or "latin america" or "south and central america" or "south america" or "asia, central" or "central asia" or "asia, northern" or "north asia" or "northern asia" or "asia, southeastern" or "southeastern asia" or "south eastern asia" or "southeast asia" or "south east asia" or "asia, western" or "western asia" or "europe, eastern" or "east europe" or "eastern europe" or "developing country" or "developing countries" or "developing nation" or "developing nations" or "developing population" or "developing populations" or "developing world" or "less developed country" or "less developed countries" or "less developed nation" or "less developed nations" or "less developed population" or "less developed populations" or "less developed world" or "lesser developed country" or "lesser developed countries" or "lesser developed nation" or "lesser developed nations" or "lesser developed population" or "lesser developed populations" or "lesser developed world" or "under developed country" or "under developed countries" or "under developed nation" or "under developed nations" or "under developed population" or "under developed populations" or "under developed world" or "underdeveloped country" or "underdeveloped countries" or "underdeveloped nation" or "underdeveloped nations" or "underdeveloped population" or "underdeveloped populations" or "underdeveloped world" or "middle income country" or "middle income countries" or "middle income nation" or "middle income nations" or "middle income population" or "middle income populations" or "low income country" or "low income countries" or "low income nation" or "low income nations" or "low income population" or "low income populations" or "lower income country" or "lower income countries" or "lower income nation" or "lower income

nations" or "lower income population" or "lower income populations" or "underserved country" or "underserved countries" or "underserved nation" or "underserved nations" or "underserved population" or "underserved populations" or "underserved world" or "under served country" or "under served countries" or "under served nation" or "under served nations" or "under served population" or " under served populations" or "under served world" or "deprived country" or "deprived countries" or "deprived nation" or "deprived nations" or "deprived population" or "deprived populations" or "deprived world" or "poor country" or "poor countries" or "poor nation" or "poor nations" or "poor population" or "poor populations" or "poor world" or "poorer country" or "poorer countries" or "poorer nation" or "poorer nations" or "poorer population" or "poorer populations" or "poorer world" or "developing economy" or "developing economies" or "less developed economy" or "less developed economies" or "lesser developed economy" or "lesser developed economies" or "under developed economy" or "under developed economies" or "underdeveloped economy" or "underdeveloped economies" or "middle income economy" or "middle income economies" or "low income economy" or "low income economies" or "lower income economy" or "lower income economies" or "low gdp" or "low gnp" or "low gross domestic" or "low gross national" or "lower gdp" or "lower gnp" or "lower gross domestic" or "lower gross national" or lmic or lmics or "third world" or "lami country" or "lami countries" or "transitional country" or "transitional countries" or "emerging economies" or "emerging economy" or "emerging nation" or "emerging nations")
4 354,340 #3 OR #2 OR #1
3 2,339 TS=((dense* or density or high* or large* or heavy or heavily) NEAR/3 (populat* or populous) NEAR/3 (area* or settlement* or district* or neighbourhood* or communit*))
2 70,206 TS=(metropolis or megalopolis or municipal* or conurbation* or suburb* or town or towns or township* or borough* or barrio or barrios)
1 309,493 TS=(urban* or city or cities or megacity or megacities or metropolitan)

Key:
* = truncation
" " = exact phrase search
? = stands for zero or one character
TS = topic search - terms in title, abstract, keyword fields
NEAR/3 = terms within three words of each other (any order)

Global Index Medicus
https://www.globalindexmedicus.net/
Searched on: 23rd June 2020
Records retrieved: 1274

6 separate searches were carried out and results deduplicated in EndNote.

1. (tw:(urban* OR city OR cities OR megacity OR megacities OR metropolitan OR metropolis OR megalopolis OR municipal* OR conurbation* OR suburb* OR town OR towns OR township* OR borough* OR barrio OR barrios OR "Urban Population" OR "Urban Health Services" OR "Hospitals, Municiple" OR "Urban Health" OR "Hospitals, Urban")) AND (ti:(cost or costs or expenditure* or expense* or spend* or pay* or paid or budget* or financ* or economic*)) AND (tw:(care OR healthcare OR health OR medical*))
2010-2020
607 hits

2. (tw:(urban* OR city OR cities OR megacity OR megacities OR metropolitan OR metropolis OR megalopolis OR municipal* OR conurbation* OR suburb* OR town OR towns OR township* OR borough* OR barrio OR barrios OR "Urban Population" OR "Urban Health Services" OR "Hospitals, Municiple" OR "Urban Health" OR "Hospitals, Urban")) AND (tw:("Health Care Costs" OR "Health Expenditures" OR "Cost of Illness" OR "Financing Personal" OR "Catastrophic Expenditure" OR "Catastrophic Illness/EC" OR catastroph* OR "out of pocket" OR oop OR oope OR "willingness to pay"))
2010-2020
530 hits

3. (tw:(slum OR slums OR ghetto OR ghettos OR shanty* OR shanties OR shack* OR favela* OR squat* OR "informal settlement" OR "informal settlements" OR "Poverty Areas")) AND (tw:(cost or costs or expenditure* or expense* or spend* or pay* or paid or budget* or financ* or economic*)) AND (tw:(care OR health OR healthcare OR medical*))
2010-2020
189 hits

4. tw:(slum OR slums OR ghetto OR ghettos OR shanty* OR shanties OR shack* OR favela* OR squat* OR "informal settlement" OR "informal settlements" OR "Poverty Areas")) AND (tw:("Health Care Costs" OR "Health Expenditures" OR "Cost of Illness" OR "Financing Personal" OR "Catastrophic Expenditure" OR "Catastrophic Illness/EC" OR catastroph* OR "out of pocket" OR oop OR oope OR "willingness to pay"))
2010-2020
10 hits

5. (tw:(urban* OR city OR cities OR megacity OR megacities OR metropolitan OR metropolis OR megalopolis OR municipal* OR conurbation* OR suburb* OR town OR towns OR township* OR borough* OR barrio OR barrios OR "Urban Population" OR "Urban Health Services" OR "Hospitals, Municiple" OR "Urban Health" OR "Hospitals, Urban")) AND (tw:(impoverish*)) AND (tw:(health OR medical*))
2010-2020
188 hits

6. (tw:(slum OR slums OR ghetto OR ghettos OR shanty* OR shanties OR shack* OR favela* OR squat* OR "informal settlement" OR "informal settlements" OR "Poverty Areas")) AND (tw:(impoverish*)) AND (tw:(health OR medical*))
2010-2020
24 hits

Key:
tw = terms in the title, abstract or subject heading fields
* = truncation
" " = exact phrase

ProQuest Dissertations & Theses A&I
via ProQuest https://www.proquest.com/
Searched on: 23rd June 2020
Records retrieved: 283

10 separate searches were carried out and results deduplicated in EndNote. The following search line was added to each search to limit retrieval to low- middle-income countries:

(TI,AB,SU(afghanistan OR albania OR algeria OR "american samoa" OR angola OR "antigua and barbuda" OR antigua OR barbuda OR argentina OR armenia OR armenian OR aruba OR azerbaijan OR bahrain OR bangladesh OR barbados OR "republic of belarus" OR belarus OR byelarus OR belorussia OR byelorussian OR belize OR "british honduras" OR benin OR dahomey OR bhutan OR bolivia OR "bosnia and herzegovina" OR bosnia OR herzegovina OR botswana OR bechuanaland OR brazil OR brasil OR bulgaria OR "burkina faso" OR "burkina fasso" OR "upper volta" OR burundi OR urundi OR "cabo verde" OR "cape verde" OR cambodia OR kampuchea OR "khmer republic" OR cameroon OR cameron OR cameroun OR "central african republic" OR "ubangi shari" OR chad OR chile OR china OR colombia OR comoros OR "comoro islands" OR "iles comores" OR mayotte OR "democratic republic of the congo" OR "democratic republic congo" OR congo OR zaire OR "costa rica" OR "cote d’ivoire" OR "cote d’ ivoire" OR "cote divoire" OR "cote d ivoire" OR "ivory coast" OR croatia OR cuba OR cyprus OR "czech republic" OR czechoslovakia OR djibouti OR "french somaliland" OR dominica OR "dominican republic" OR ecuador OR egypt OR "united arab republic" OR "el salvador" OR "equatorial guinea" OR "spanish guinea" OR eritrea OR estonia OR eswatini OR swaziland OR ethiopia OR fiji OR gabon OR "gabonese republic" OR gambia OR "georgia (republic) " OR georgian OR ghana OR "gold coast" OR gibraltar OR greece OR grenada OR guam OR guatemala OR guinea OR "guinea bissau" OR guyana OR "british guiana" OR haiti OR hispaniola OR honduras OR hungary OR india OR indonesia OR timor OR iran OR iraq OR "isle of man" OR jamaica OR jordan OR kazakhstan OR kazakh OR kenya OR "democratic people’s republic of korea" OR "republic of korea" OR "north korea" OR "south korea" OR korea OR kosovo OR kyrgyzstan OR kirghizia OR kirgizstan OR "kyrgyz republic" OR kirghiz OR laos OR "lao pdr" OR "lao people's democratic republic" OR latvia OR lebanon OR "lebanese republic" OR lesotho OR basutoland OR liberia OR libya OR "libyan arab jamahiriya" OR lithuania OR macau OR macao OR "macedonia (republic) " OR macedonia OR madagascar OR "malagasy republic" OR malawi OR nyasaland OR malaysia OR "malay federation" OR "malaya federation" OR maldives OR "indian ocean islands" OR "indian ocean" OR mali OR malta OR micronesia OR "federated states of micronesia" OR kiribati OR "marshall islands" OR nauru OR "northern mariana islands" OR palau OR tuvalu OR mauritania OR mauritius OR mexico OR moldova OR moldovian OR mongolia OR montenegro OR morocco OR ifni OR mozambique OR "portuguese east africa" OR myanmar OR burma OR namibia OR nepal OR "netherlands antilles" OR nicaragua OR niger OR nigeria OR oman OR muscat OR pakistan OR panama OR "papua new guinea" OR "new guinea" OR paraguay OR peru OR philippines OR philipines OR phillipines OR phillippines OR poland OR "polish people's republic" OR portugal OR "portuguese republic" OR "puerto rico" OR romania OR russia OR "russian federation" OR ussr OR "soviet union" OR "union of soviet socialist republics" OR rwanda OR ruanda OR samoa OR "pacific islands" OR polynesia OR "samoan islands" OR "navigator island" OR "navigator islands" OR "sao tome and principe" OR "saudi arabia" OR senegal OR serbia OR seychelles OR "sierra leon" e OR slovakia OR "slovak republic" OR slovenia OR melanesia OR "solomon island" OR "solomon islands" OR "norfolk island" OR "norfolk islands" OR somalia OR "south africa" OR "south sudan" OR "sri lanka" OR ceylon OR "saint kitts and nevis" OR "st. kitts and nevis" OR "saint lucia" OR "st. lucia" OR "saint vincent and the grenadines" OR "saint vincent" OR "st. vincent" OR grenadines OR sudan OR suriname OR surinam OR "dutch guiana" OR "netherlands guiana" OR syria OR "syrian arab republic" OR tajikistan OR tadjikistan OR tadzhikistan OR tadzhik OR tanzania OR tanganyika OR thailand OR siam OR "timor leste" OR "east timor" OR togo OR "togolese republic" OR tonga OR "trinidad and tobago" OR trinidad OR tobago OR tunisia OR turkey OR "turkey (republic) " OR turkmenistan OR turkmen OR uganda OR ukraine OR uruguay OR uzbekistan OR uzbek OR vanuatu OR "new hebrides" OR venezuela OR vietnam OR "viet nam" OR "middle east" OR "west bank" OR gaza OR palestine OR yemen OR yugoslavia OR zambia OR zimbabwe OR "northern rhodesia" OR "global south" OR "africa south of the sahara" OR "sub-saharan africa" OR "subsaharan africa" OR "africa, central" OR "central africa" OR "africa, northern" OR "north africa" OR "northern africa" OR magreb OR maghrib OR sahara OR "africa, southern" OR "southern africa" OR "africa, eastern" OR "east africa" OR "eastern africa" OR "africa, western" OR "west africa" OR "western

africa" OR "west indies" OR "indian ocean islands" OR caribbean OR "central america" OR "latin america" OR "south and central america" OR "south america" OR "asia, central" OR "central asia" OR "asia, northern" OR "north asia" OR "northern asia" OR "asia, southeastern" OR "southeastern asia" OR "south eastern asia" OR "southeast asia" OR "south east asia" OR "asia, western" OR "western asia" OR "europe, eastern" OR "east europe" OR "eastern europe" OR "developing country" OR "developing countries" OR "developing nation" OR "developing nations" OR "developing population" OR "developing populations" OR "developing world" OR "less developed country" OR "less developed countries" OR "less developed nation" OR "less developed nations" OR "less developed population" OR "less developed populations" OR "less developed world" OR "lesser developed country" OR "lesser developed countries" OR "lesser developed nation" OR "lesser developed nations" OR "lesser developed population" OR "lesser developed populations" OR "lesser developed world" OR "under developed country" OR "under developed countries" OR "under developed nation" OR "under developed nations" OR "under developed population" OR "under developed populations" OR "under developed world" OR "underdeveloped country" OR "underdeveloped countries" OR "underdeveloped nation" OR "underdeveloped nations" OR "underdeveloped population" OR "underdeveloped populations" OR "underdeveloped world" OR "middle income country" OR "middle income countries" OR "middle income nation" OR "middle income nations" OR "middle income population" OR "middle income populations" OR "low income country" OR "low income countries" OR "low income nation" OR "low income nations" OR "low income population" OR "low income populations" OR "lower income country" OR "lower income countries" OR "lower income nation" OR "lower income nations" OR "lower income population" OR "lower income populations" OR "underserved country" OR "underserved countries" OR "underserved nation" OR "underserved nations" OR "underserved population" OR "underserved populations" OR "underserved world" OR "under served country" OR "under served countries" OR "under served nation" OR "under served nations" OR "under served population" OR " under served populations" OR "under served world" OR "deprived country" OR "deprived countries" OR "deprived nation" OR "deprived nations" OR "deprived population" OR "deprived populations" OR "deprived world" OR "poor country" OR "poor countries" OR "poor nation" OR "poor nations" OR "poor population" OR "poor populations" OR "poor world" OR "poorer country" OR "poorer countries" OR "poorer nation" OR "poorer nations" OR "poorer population" OR "poorer populations" OR "poorer world" OR "developing economy" OR "developing economies" OR "less developed economy" OR "less developed economies" OR "lesser developed economy" OR "lesser developed economies" OR "under developed economy" OR "under developed economies" OR "underdeveloped economy" OR "underdeveloped economies" OR "middle income economy" OR "middle income economies" OR "low income economy" OR "low income economies" OR "lower income economy" OR "lower income economies" OR "low gdp" OR "low gnp" OR "low gross domestic" OR "low gross national" OR "lower gdp" OR "lower gnp" OR "lower gross domestic" OR "lower gross national" OR lmic OR lmics OR "third world" OR "lami country" OR "lami countries" OR "transitional country" OR "transitional countries" OR "emerging economies" OR "emerging economy" OR "emerging nation" OR "emerging nations")

1. LMIC strategy as above

AND (TI,AB,SU(urban* OR city OR cities OR megacity OR megacities OR metropolitan OR metropolis OR megalopolis OR municipal* OR conurbation* OR suburb* OR town OR towns OR township* OR borough* OR barrio OR barrios) OR TI,AB,SU((dense* OR density OR high* OR large* OR heavy OR heavily) NEAR/3 (populat OR populous) NEAR/3 (area* OR settlement* OR district* OR neighbourhood* OR communit*)))) AND (TI,AB,SU(cost* NEAR/2 (illness* OR sickness* OR disease*)) OR TI,AB,SU(cost* NEAR/3 (care OR health OR healthcare OR medical*)) OR TI,AB,SU(expenditure* NEAR/3 (care OR health OR healthcare OR medical*)))
Date limit: 2010-01-01 - 2020-06-23
164 hits

2. LMIC strategy as above
AND (TI,AB,SU(urban* OR city OR cities OR megacity OR megacities OR metropolitan OR metropolis OR megalopolis OR municipal* OR conurbation* OR suburb* OR town OR towns OR township* OR borough* OR barrio OR barrios) OR TI,AB,SU((dense* OR density OR high* OR large* OR heavy OR heavily) NEAR/3 (populat OR populous) NEAR/3 (area* OR settlement* OR district* OR neighbourhood* OR communit*))) AND (TI,AB,SU((direct OR indirect) NEAR/2 (cost* OR expenditure*)) OR TI,AB,SU("out of pocket") OR TI,AB,SU(OOP NEAR/3 (cost* OR expense* OR expenditure* OR financ* OR pay* OR paid OR spend*)) OR TI,AB,SU(OOPE))
Date limit: 2010-01-01 - 2020-06-23
52 hits

3. LMIC strategy as above
AND (TI,AB,SU(urban* OR city OR cities OR megacity OR megacities OR metropolitan OR metropolis OR megalopolis OR municipal* OR conurbation* OR suburb* OR town OR towns OR township* OR borough* OR barrio OR barrios) OR TI,AB,SU((dense* OR density OR high* OR large* OR heavy OR heavily) NEAR/3 (populat OR populous) NEAR/3 (area* OR settlement* OR district* OR neighbourhood* OR communit*))) AND TI,AB,SU((personal OR individual* OR patient* OR family* OR families OR household*) NEAR/3 (budget* OR cost* OR expense* OR expenditure* OR financ* OR pay* OR paid OR spend*) NEAR/3 (care OR health OR healthcare OR medical*))
Date limit: 2010-01-01 - 2020-06-23
27 hits

4. LMIC strategy as above
AND (TI,AB,SU(urban* OR city OR cities OR megacity OR megacities OR metropolitan OR metropolis OR megalopolis OR municipal* OR conurbation* OR suburb* OR town OR towns OR township* OR borough* OR barrio OR barrios) OR TI,AB,SU((dense* OR density OR high* OR large* OR heavy OR heavily) NEAR/3 (populat OR populous) NEAR/3 (area* OR settlement* OR district* OR neighbourhood* OR communit*))) AND TI,AB,SU(catastroph* NEAR/10 (cost* OR expense* OR expenditure* OR financ* OR pay* OR paid OR spend*))
Date limit: 2010-01-01 - 2020-06-23
8 hits

5. LMIC strategy as above
AND (TI,AB,SU(urban* OR city OR cities OR megacity OR megacities OR metropolitan OR metropolis OR megalopolis OR municipal* OR conurbation* OR suburb* OR town OR towns OR township* OR borough* OR barrio OR barrios) OR TI,AB,SU((dense* OR density OR high* OR large* OR heavy OR heavily) NEAR/3 (populat OR populous) NEAR/3 (area* OR settlement* OR district* OR neighbourhood* OR communit*))) AND TI,AB,SU((willing* OR unwilling*) NEAR/3 (pay*) NEAR/3 (care OR health OR healthcare OR medical*))
Date limit: 2010-01-01 - 2020-06-23
1 hit

6. LMIC strategy as above
AND (TI,AB,SU(urban* OR city OR cities OR megacity OR megacities OR metropolitan OR metropolis OR megalopolis OR municipal* OR conurbation* OR suburb* OR town OR towns OR township* OR borough* OR barrio OR barrios) OR TI,AB,SU((dense* OR density OR high* OR large* OR heavy OR heavily) NEAR/3 (populat OR populous) NEAR/3 (area* OR settlement* OR district* OR neighbourhood* OR communit*))) AND (TI,AB,SU((medical* OR health) NEAR/3 impoverish*) OR TI,AB,SU(burden* NEAR/3 (cost* OR expenditure* OR economic* OR financ*) NEAR/3 (care OR

health OR healthcare OR medical*)) OR TI,AB,SU((afford* OR unafford*) NEAR/5 (cost* OR pay*)) OR TI,AB,SU((able OR ability OR capacit* OR unable OR inability) NEAR/2 pay*))
Date limit: 2010-01-01 - 2020-06-23
81 hits

7. LMIC strategy as above
AND (TI,AB,SU(slum OR slums OR ghetto OR ghettos OR shanty* OR shanties OR shack* OR favela*) OR TI,AB,SU((informal* OR precarious* OR irregular*) NEAR/3 settlement*) OR TI,AB,SU((poverty OR impoverish* OR squatter*) NEAR/3 (area* OR settlement*))) AND ((TI,AB,SU(cost* NEAR/2 (illness* OR sickness* OR disease*)) OR TI,AB,SU(cost* NEAR/3 (care OR health OR healthcare OR medical*)) OR TI,AB,SU(expenditure* NEAR/3 (care OR health OR healthcare OR medical*))) OR (TI,AB,SU((direct OR indirect) NEAR/2 (cost* OR expenditure*)) OR TI,AB,SU("out of pocket") OR TI,AB,SU(OOP NEAR/3 (cost* OR expense* OR expenditure* OR financ* OR pay* OR paid OR spend*)) OR TI,AB,SU(OOPE)))
Date limit: 2010-01-01 - 2020-06-23
15 hits

8. LMIC strategy as above
AND (TI,AB,SU(slum OR slums OR ghetto OR ghettos OR shanty* OR shanties OR shack* OR favela*) OR TI,AB,SU((informal* OR precarious* OR irregular*) NEAR/3 settlement*) OR TI,AB,SU((poverty OR impoverish* OR squatter*) NEAR/3 (area* OR settlement*))) AND TI,AB,SU((personal OR individual* OR patient* OR family* OR families OR household*) NEAR/3 (budget* OR cost* OR expense* OR expenditure* OR financ* OR pay* OR paid OR spend*) NEAR/3 (care OR health OR healthcare OR medical*))
Date limit: 2010-01-01 - 2020-06-23
3 hits

9. LMIC strategy as above
AND TI,AB,SU(slum OR slums OR ghetto OR ghettos OR shanty* OR shanties OR shack* OR favela*) OR TI,AB,SU((informal* OR precarious* OR irregular*) NEAR/3 settlement*) OR TI,AB,SU((poverty OR impoverish* OR squatter*) NEAR/3 (area* OR settlement*))) AND TI,AB,SU(catastroph* NEAR/10 (cost* OR expense* OR expenditure* OR financ* OR pay* OR paid OR spend*))
Date limit: 2010-01-01 - 2020-06-23
2 hits

10. LMIC strategy as above
AND (TI,AB,SU(slum OR slums OR ghetto OR ghettos OR shanty* OR shanties OR shack* OR favela*) OR TI,AB,SU((informal* OR precarious* OR irregular*) NEAR/3 settlement*) OR TI,AB,SU((poverty OR impoverish* OR squatter*) NEAR/3 (area* OR settlement*))) AND (TI,AB,SU((willing* OR unwilling*) NEAR/3 (pay*) NEAR/3 (care OR health OR healthcare OR medical*)) OR (TI,AB,SU((medical* OR health) NEAR/3 impoverish*) OR TI,AB,SU(burden* NEAR/3 (cost* OR expenditure* OR economic* OR financ*) NEAR/3 (care OR health OR healthcare OR medical*)) OR TI,AB,SU((afford* OR unafford*) NEAR/5 (cost* OR pay*)) OR TI,AB,SU((able OR ability OR capacit* OR unable OR inability) NEAR/2 pay*)))
Date limit: 2010-01-01 - 2020-06-23
9 hits

Key:
TI,AB,SU = terms in title, abstract, or subject heading fields
* = truncation
" "   = exact phrase search

NEAR/3 = terms within three words of each other (any order)

Econpapers
https://econpapers.repec.org/

OpenGrey
http://www.opengrey.eu/

World Bank

OECD

References

1. Effective Practice and Organisation of Care (EPOC) Cochrane group. LMIC filters. The Cochrane Collaboration. 2020 [accessed 14th May 2020]. Available from: https://epoc.cochrane.org/lmic-filters
