# Supplementary material for: Economics of healthcare access in low-income and middle-income countries: a protocol for a scoping review of the economic impacts of seeking healthcare on slum-dwellers compared with other city residents
Source: BMJ Open. 2021 Jul 9;11(7):e045441. doi: 10.1136/bmjopen-2020-045441 (PMC8273471; doi:10.1136/bmjopen-2020-045441)
Supplement: Author's manuscript [file bmjopen-2020-045441.draft_revisions.pdf]

BMJ Open

BMJ Open is committed to open peer review. As part of this commitment we make the peer review history of every article we publish publicly available.

When an article is published we post the peer reviewers' comments and the authors' responses online. We also post the versions of the paper that were used during peer review. These are the versions that the peer review comments apply to.

The versions of the paper that follow are the versions that were submitted during the peer review process. They are not the versions of record or the final published versions. They should not be cited or distributed as the published version of this manuscript.

BMJ Open is an open access journal and the full, final, typeset and author-corrected version of record of the manuscript is available on our site with no access controls, subscription charges or pay-per-view fees (http://bmjopen.bmj.com).

If you have any questions on BMJ Open's open peer review process please email info.bmjopen@bmj.com

BMJ Open

The economics of health-care access in low and middle-income countries: A protocol for a scoping review of the economic impacts of seeking health care on slum-dwellers compared with other city residents.

Journal:	*BMJ Open*
Manuscript ID	bmjopen-2020-045441
Article Type:	Protocol
Date Submitted by the Author:	01-Oct-2020
Complete List of Authors:	Teixeira de Siqueira-Filha, Noemia; University of York<br>Li, Jinshuo; University of York, Department of Health Sciences<br>Kibuchi, Eliud; University of Glasgow<br>Quayyum , Zahidul ; BRAC University James P Grant School of Public Health,<br>Phillips-Howard, Penelope; Liverpool School of Tropical Medicine, Department of Clinical Sciences<br>Awal, Abdul; BRAC<br>Mithu, Md. Imran Hossain; BRAC<br>Manzoor, Farzana; BRAC<br>Karunga, Robinson; LVCT<br>Saidu, Samuel; COMAHS<br>Smith, John; COMAHS<br>Sai, Varun; The George Institute for Global Health India<br>Garimella, Sureka; The George Institute for Global Health India<br>Chumo, Ivy; African Population and Health Research Center<br>Mberu, Blessing; African Population and Health Research Center<br>Tolhurst, Rachel; Liverpool School of Tropical Medicine, Clinical Sciences and International Public Health<br>Mazumdar, Sumit; Centre for Health Economics<br>Rao, Vinodkumar ; The Society for Promotion of Area Resource Center<br>Farnaz, Nadia; BRAC University James P Grant School of Public Health, School of Public Health; BRAC University James P Grant School of Public Health, School of Public Health<br>Alam, Wafa; BRAC University,<br>Elsey, Helen ; University of York,
Keywords:	HEALTH ECONOMICS, HEALTH SERVICES ADMINISTRATION & MANAGEMENT, Health policy < HEALTH SERVICES ADMINISTRATION & MANAGEMENT, Protocols & guidelines < HEALTH SERVICES ADMINISTRATION & MANAGEMENT

SCHOLARONE™
Manuscripts

I, the Submitting Author has the right to grant and does grant on behalf of all authors of the Work (as defined in the below author licence), an exclusive licence and/or a non-exclusive licence for contributions from authors who are: i) UK Crown employees; ii) where BMJ has agreed a CC-BY licence shall apply, and/or iii) in accordance with the terms applicable for US Federal Government officers or employees acting as part of their official duties; on a worldwide, perpetual, irrevocable, royalty-free basis to BMJ Publishing Group Ltd ("BMJ") its licensees and where the relevant Journal is co-owned by BMJ to the co-owners of the Journal, to publish the Work in this journal and any other BMJ products and to exploit all rights, as set out in our licence.

The Submitting Author accepts and understands that any supply made under these terms is made by BMJ to the Submitting Author unless you are acting as an employee on behalf of your employer or a postgraduate student of an affiliated institution which is paying any applicable article publishing charge ("APC") for Open Access articles. Where the Submitting Author wishes to make the Work available on an Open Access basis (and intends to pay the relevant APC), the terms of reuse of such Open Access shall be governed by a Creative Commons licence – details of these licences and which Creative Commons licence will apply to this Work are set out in our licence referred to above.

Other than as permitted in any relevant BMJ Author's Self Archiving Policies, I confirm this Work has not been accepted for publication elsewhere, is not being considered for publication elsewhere and does not duplicate material already published. I confirm all authors consent to publication of this Work and authorise the granting of this licence.

For peer review only

Title: The economics of health-care access in low and middle-income countries: A protocol for a scoping review of the economic impacts of seeking health care on slum-dwellers compared with other city residents.

Collaborating authors: [1]Noemia Siqueira-Filha, [1]Jinshuo Li, [2]Eliud Kibuchi, [3]Zahidul Quayyum, [4]Penelope A Phillips-Howard, [3]Abdul Awal, [3]Imran Mithu, [3]Farzana Manzoor, [5]Robinson Karuga, [6]Samuel Saidu, [6]John Smith, [7]Varun Sai, [7]Sureka Garimella, [8] Ivy Chumo, [8] Blessing Mberu, [4]Rachel Tolhurst, [1]Sumit Mazumdar,[9]Vinodkumar Rao, [3]Nadia Farnaz, [3]Wafa Alam, [1]Helen Elsey

Noemia.teixeira.siqueira@gmail.com, jinshuo.li@york.ac.uk, Eliud.Kibuchi@glasgow.ac.uk, zahidul.quayyum@bracu.ac.bd, Penelope.Phillips-Howard@lstmed.ac.uk, abdul.awal@bracu.ac.bd, imran.mithu@bracu.ac.bd, farzana.manzoor@bracu.ac.bd, Robinson.Karuga@lvcthealth.org, samuelsaidu0@gmail.com, smithhawa95@gmail.com, varun.sbbkey@gmail.com, sgarimella@georgeinstitute.org.in, ichumo@aphrc.org, bmberu@aphrc.org, Rachel.Tolhurst@lstmed.ac.uk, sumit.mazumdar@york.ac.uk, vinodkumar.rao@gmail.com, nadia.farnaz@bracu.ac.bd, wafa.alam@bracu.ac.bd, helen.elsey@york.ac.uk

[1] University of York, York, UK
[2] University of Glasgow, Glasgow, UK
[3] James P Grant School of Public Health, Brac University, Dhaka, Bangladesh
[4] Liverpool School of Tropical Medicine, Liverpool, UK
[5] LVCT Health, Nairobi, Kenya
[6] COMAHS: University of Sierra Leone, Freetown, Sierra Leone,
[7] The George Institute for Global Health, New Delhi, India
[8] African Population and Health Research Center, Nairobi, Kenya
[9] The Society for Promotion of Area Resource Centres, Mumbai, India

Corresponding author: Noemia Teixeira de Siqueira Filha, Noemia.teixeira.siqueira@gmail.com, Department of Health Sciences, Faculty of Science, University of York, Seebohm Rowntree Building (Area 2), University of York, Heslington, York-UK, YO10 5DD.

Key words: scoping review, informal settlements, costs, catastrophic health expenditures, low-middle-income countries

Word count: 2,110

For peer review only

For peer review only - http://bmjopen.bmj.com/site/about/guidelines.xhtml

Strengths and limitations of this study

- Slum communities are extremely vulnerable and marginalized, reviews on the economic burden of accessing health care can contribute to inform decisions on how best to remodel urban health systems and improve equitable access to these communities.
- There is no language restriction in this review, per reviewed studies, grey literature and reports from12 databases including leading organizations on urban health research will be searched.
- We will assess the quality of studies by using the Consolidated Health Economic Evaluation Reporting Standards and the Tool to Estimate Patient's Costs.
- Slums communities in rural areas will not be included in this review.

For peer review only

For peer review only - http://bmjopen.bmj.com/site/about/guidelines.xhtml

Abstract

Introduction: People living in slums face several challenges to access healthcare. Scarce and low-quality public health facilities are common problems in these communities. Costs and prevalence of catastrophic health expenditures (CHE) have also been reported as high in studies conducted in slums in developing countries and those suffering from chronic conditions and the poorest households seem to be more vulnerable to financial hardship. The Covid-19 pandemic may be aggravating the economic impact on the extremely vulnerable population living in slums due to the long-term consequences of the disease. The objective of this review is to report the economic impact of seeking health care on slum-dwellers in terms of costs and CHE. We will compare the economic impact on slum-dwellers with other city residents.

Methods and analysis: This scoping review adopts the framework suggested by Arksey and O'Malley. The review is part of the ARISE research consortium, which aims to enhance accountability to improve the health and wellbeing of marginalized populations living in slums in India, Bangladesh, Sierra Leone, and Kenya. Costs of accessing health care will be updated to 2020 prices using the inflation rates reported by the International Monetary Fund. Costs will be presented in International Dollars by using purchase power parity. The prevalence of CHE will also be reported.

Ethics and dissemination: Ethical approval is not required for scoping reviews. We will disseminate our results alongside the events organized by the ARISE consortium and international conferences. The final manuscript will be submitted to an open-access international journal.

For peer review only - http://bmjopen.bmj.com/site/about/guidelines.xhtml

1. Background

The United Nations estimates that an increase in population and migration will add a further 2.5 billion people to the urban population by 2050. Ninety per cent of this growth will occur in Asian and African countries.[1] In 2014, 30% of the total urban population in developing countries were living in slums.[2] The absolute number of 881,080 people is close to the entire population of the two most populous countries in the world, India (1.4 billion inhabitants) and China (1.3 billion inhabitants).[3] While urbanization is one possible driver of economic growth,[4] it also increases inequities within cities and those living in slums suffer grossly inadequate access to health care, clean water, sanitation, safe housing and basic amenities.[5]

Although the traditional assumption describes an urban advantage in accessing health-care,[6] many barriers to access remain, and physical proximity to health providers does not always translate to better health, particularly for the urban poor.[7] While analysis of demographic and health surveys (DHSs) across 73 low and middle-income countries (LMICs) showed that children in urban slums have better health than their rural counterparts,[8] under 5 mortality in Kenya and Bangladesh was higher in urban slums than in rural areas (Kenya: 79.8 vs 56 per 1000 live births; Bangladesh: 57 vs 49 per 1000 live births).[7,9] These conflicting data may in part be due to limitations in the DHS sampling methods where urban poor were under-represented.[10,11]

Urbanisation has been seen as a determinant of health and wellbeing, resulting in a double burden of communicable and non-communicable diseases (NCDs).[12] A spectrum of research has shown how slums constitute unhealthy places, posing critical health challenges for urban residents. Children living in slums are particularly vulnerable to malnutrition, recurrent diarrhoea, stunted growth, and long-term cognitive development challenges.[13,14] Prevalence of diabetes and hypertension,[6,15] and poor mental health have increased dramatically in slums.[7,8] Injuries, both unintentional and as a result of violence,[16] nutrition transition,[17,18] obesity,[19] tobacco,[20,21] and alcohol abuse[22,23] are also disproportionately high in slum communities. All of these studies highlight how these poor health conditions are driven by the stresses, work patterns and changing social and gender norms within cities. There is also increasing evidence on the role of socioeconomic factors contributing to common forms of mental illness in slum-dwellers, further exacerbating the burden of disease and inequities related to mental health in these communities.[24]

Provider pluralism is common in many cities in LMICs and some governments have encouraged the adoption of pluralistic models for healthcare delivery in slums, stablishing public-private partnerships between Non-Governmental Organizations (NGOs), government and private providers. The Urban Primary Health Care Programme project, implemented by the government of Bangladesh, has positively influenced health outcomes for the urban poor in the country.[25] NGOs have frequently led the way in providing health care services for slum-dwellers, leading to some reduction in intra-urban disparities, particularly in terms of reproductive health.[26]

While this pluralism of providers may improve physical access of poor urban communities to health-care, several studies have described the barriers faced by slum-dweller on the pathway to health services. Contentious relationships between city authorities and slums-dwellers,[27] and scant public health services whose the access needs to be negotiated through a 'middlemen'[28] have been reported in several slums. High out of pocket expenditure of urban residents, limited coverage of health insurance and access to subsidised government schemes, particularly among migrants who frequently lack the required registration documents[29–31] are also common in these spaces. In addition, important differences in costs of accessing health care for chronic conditions were found among and within slums in Bangladesh. Slum-dwellers in Tongi community incurred doble the costs of those residents of Sylhet (US$ 323 vs USD 169, 2014 prices). Whlist the wealthiest households (4th and 5th quintiles) were more likely to incur higher costs compared to the poorest households (1st and 2nd quintiles) at the same slum.[31] The prevalence of catastrophic health expenditure (CHE) has has also been reported as high. The degree of CHE varies according to the threshold and methods

For peer review only - http://bmjopen.bmj.com/site/about/guidelines.xhtml

adopted to calculate this indicator.[32] The economic and social impacts of seeking health care for slum-dwellers may be further exacerbated by the Covid-19 outbreak due to the severity of the symptoms and its long-term consequences. Several reports have shown high incidence and mortality due to Covid-19 in slums areas of Chembur, Matunga and Dahisar in Mumbai, India.[33,34]

By understanding the structure of expenditure of urban poor residents on health care, we will be able to inform decisions on how best to remodel urban health systems and improve equitable access. The objective of this scoping review is therefore to map the evidence available on the economic impact of seeking health care on slum-dwellers compared with other city residents. We will quantify the economic impact in terms of CHE, direct and indirect costs incurred by these communities during the search for health care and treatment.

Methods

This scoping review will be developed as part of the ARISE research consortium, funded by the Global Challenges Research Fund (GCRF). The consortium aims to enhance accountability and improve the equitable health and wellbeing of marginalised populations living in slums in LMICs. The review is part of the ARISE work package *Metrics, Epidemiology, and Economics,* which aims to collect metrics to reflect lived realities, inequities, and priorities for transformative social changes in slums by using epidemiological and health economics tools.[35]

This review protocol follows the framework suggested by Arksey and O'Malley, 2005[36] and Levac et al, 2010[37] and It was registered at the Research Registry (https://www.researchregistry.com/), ID: reviewregistry947.

Identifying the research question

We formulated our research question based on the ARISE principle to generate evidence to promote accountability and responsiveness of slum-dwellers (ARISE, 2019). The PCC (population, concept, and context) framework was used to formulate the research question.[38] We will explore the concept of the economic impact of seeking health care on slum communities compared with other city-residents in the context of urban areas in LMICs. Box 1 shows the glossary of operational definitions adopted in this review.

For peer review only - http://bmjopen.bmj.com/site/about/guidelines.xhtml

Box 1. Glossary of operational definitions.

Low and middle income countries: Gross National Income (GNI) per capita of $1,035 or less for low income economies; GNI per capita between $1,036 and $4,045 for lower middle-income economies; and GNI per capita between $4,046 and $12,535 for upper middle-income economies.[3]
Urban informal settlements, slums: households living in urban areas who lack at least one of the following: security of housing tenure, easy access to water or sanitation, sufficient living space, durable housing.[39]
Economic impact: in this review, the economic impact is defined as the financial impact in terms of prevalence of catastrophic health expenditure, direct, indirect and total costs.
Catastrophic health expenditures (CHE): proportion of the household annual income committed with health care that results in financial hardship for the family. The World Health Organization (WHO) indicates a threshold of 40% of a household's non-subsistence income.[40] However, other thresholds have been applied in the calculation of CHE.
Direct costs: all costs due to resource use that are completely attributable to the use of a health care intervention or illness.[41]
Direct medical costs: out of pocket expenses incurred with medication, tests and health care facilities fees [41].
Direct non-medical costs: out of pocket expenses incurred with transportation, food, accommodation and care-givers.[41]
Indirect costs: income and time lost during the search for health care, while waiting for appointments and during hospitalization.[41]

Search strategy and identification of relevant studies

The search strategy aimed to identify studies examining the costs of healthcare for slum dwellers or city residents in LMICs. A search strategy was developed by an information specialist using Ovid MEDLINE and based on the review eligibility criteria indicated in Table 1.

Search terms were gathered by applying key concepts: slum dwellers, slums, informal settlements, and urban areas (population), healthcare costs (concept), and LMICs (context). Both text word and subject heading searches for each concept were included in the strategy. Retrieval was limited to publications within the last 10 years (2010-2020). No language restrictions were applied. The final search strategy for MEDLINE was agreed by NTSF and HE and then translated as appropriate for the other databases and resources. The complete search strategy is presented in the supplementary material.

Databases containing literature from the fields of health, economics and social science was searched in MEDLINE (Ovid), Embase (Ovid), EconLit (Ovid), Science Citation Index (Web of Science), Social Science Citation Index (Web of Science) and Global Index Medicus. Also, the following resources containing grey literature such as theses and dissertations, and reports from leading organizations on urban health research were searched: Proquest Dissertations and Theses (A&I), Econpapers, OpenGrey, the World Bank, the Organisation for Economic Co-operation and Development (OECD) and The UN-Habitat websites. EndNote was used for reference management and duplicate removal.

For peer review only - http://bmjopen.bmj.com/site/about/guidelines.xhtml

Table 1. Inclusion and exclusion criteria of the scoping review.

Appraisal	Inclusion Criteria	Exclusion Criteria
Country	Low and middle-income countries	High income countries
Article type	Peer reviewed published articles, theses and reports from the World Bank, Organisation for Economic Co-operation and Development and UN-Habitat	Case reports, protocols, news article, editorial, conference abstracts, comments
Study design	Economic evaluations, cost and cost-effectiveness studies	Willingness to pay, health finance and economic evaluations addressing the provider perspective, systematic and scoping reviews
Focus of study	Access to health care: NGOs, private and public sector, outpatient and inpatient care. Studies that disaggregate by slum/non-slum or wealth quintile/poor/non-poor; studies that are slum specific	Studies that do not disaggregate between rural and urban or urban wealth categories/slum and city level; studies focused on rural areas

Selection of papers and data extraction

Two reviewers will independently screen the studies by title and abstract generating two lists of retrieved studies to be compared following the pathway indicated on Figure 1. The number of records identified, duplicates removed, titles and abstracts screened, studies retrieved and included in the review will be recorded in a PRISMA flowchart.[42] Rayyan software (http://rayyan.qcri.org)[43] will be used to manage the screening of all identified studies.

The data extraction will also be conducted by two reviewers, who will extract the data using a pre-designed extraction form in the format of Excel spreadsheet. Discordances in the inclusion of studies and data extraction will be discussed with a third researcher and agreement reached by consensus. We will extract the following information: location (country/city), characteristics of the health sector, socio-economic profile of the study population, study design, sampling method, and sample size, method of data collection and calculation of costs, types of health-care services used, types of health conditions, quality of health care, study outcomes (costs and catastrophic health expenditure). Data will be classified into the following cost components: direct medical costs (consultations, tests, medicines, and hospitalization, etc.), direct non-medical cost (transport and food during healthcare visits) and indirect costs (income and time lost). If data allow, costs will be stratified by socioeconomic status, sex, age, ethnicity, health condition, Kind of health service sought and obtained, and regions (slum, non-slum, other). This data extraction strategy will be piloted and adjusted, if necessary.

Quality assessment

Although quality assessment is not a mandatory step for scoping reviews, we will examine the methodological quality of the studies by using checklists recommended for costing and cost-effectiveness studies.

We will assess the quality of studies based on the Consolidated Health Economic Evaluation Reporting Standards (CHEERS).[44] We will also use the Tool to Estimate Patient's Costs (TBCA)[45] to evaluate methods applied to estimate costs and procedures adopted for interviewing patients. The quality assessment will be focused on the methods reported in the studies through the assessment of the criteria indicated in Table 2.

For peer review only - http://bmjopen.bmj.com/site/about/guidelines.xhtml

Table 2. Items included in the quality assessment of the manuscripts

**Consolidated Health Economic Evaluation Reporting Standards**	Cost estimation methods
	Target population
	Setting and location
	Study perspective
	Sources used for resource quantities and unit costs
	Period for resources estimation; quantities and unit costs
	Methods for adjusting unit costs to the reporting year and performing currency conversion
	Values for main categories of estimated costs
	Choice of discount rate(s) used for costs and outcomes and why (studies with time horizon above one year are expected to discount rates to adjust costs estimates.)
	Time horizon over which costs and consequences are being evaluated and why
**Tool to Estimate Patient's Costs**	Patient interview procedures
	Methods used for valuing indirect costs

Collating, summarizing, and reporting the data

Costs reported before 2020 will be updated using USD inflation rates.[46] Costs reported in local currency will then be converted to US Dollars using exchange rates as reported in the OANDA website (OANDA, 2018). To allow comparison of results reported by different countries, costs will be presented in International Dollars ($) applying purchase power parity,[47] 2020 prices. The conversation to international dollars will apply World Bank indices.[48] Results will be presented as the average cost of seeking health care comparing slum dwellers vs other city residents. We will first estimate average total costs and average direct medical, non-medical and indirect costs. When available, we will also estimate disaggregated data on cost by health sector (public/private/NGO; Formal vs Informal), by wealth quintile or household assets, and by disease profile (Non-communicable diseases, communicable diseases, accidents and violence). The range of reported means across studies, unweighted average of means (with standard deviation), and the median of means (with interquartile range) will be presented for each costing category. The prevalence of CHE will be reported as range, unweighted average, and median. For studies reporting data from several countries, each country will be analysed as a separate observation.

We will report our review following the guideline Preferred Reporting Items for Systematic reviews and Meta-Analyses extension for Scoping Reviews (PRISMA-ScR) Checklist (Tricco *et al.*, 2018). Results will be synthesized and presented using a narrative and tables.

Ethics

Ethical approval is not required for scoping reviews.

Patient and public involvement

We formulated our research question based on the ARISE principle to generate evidence to promote accountability and responsiveness of slum-dwellers. Results generated by this review will potentially

For peer review only - http://bmjopen.bmj.com/site/about/guidelines.xhtml

guide the decision-making process to improve access to health care, and alleviate the economic impact of diseases on slum communities.

Our review team includes participants from all ARISE partners: Liverpool School of Tropical Medicine, University of York and University of Glasgow in UK; Brac University in Bangladesh; LVCT Health and African Population and Health Research Center in Kenya; COMAHS: University of Sierra Leone in Sierra Leone; The George Institute for Global Health and The Society for Promotion of Area Resource Centres in India. The review has a strong capacity strengthening element and all reviewers are completing the Cochrane interactive learning modules (https://training.cochrane.org/interactivelearning). The team is also meeting regularly to develop skills and ensure quality and consistency throughout the review.

Patients are not directly involved in the review process as this is based on secondary data. However, we will disseminate the results of this review alongside the events organised by the ARISE hub, such as project meetings, seminars, webinars, and capacity building training programme. Additionally, we will produce policy briefs including key results of the review, knowledge gaps and priority actions for representatives of the slum communities, policy-makers and other stakeholders in the countries where the ARISE project is working (India, Bangladesh, Sierra Leone, Kenya).

This scoping review addresses a topic of interest to the Sustainable Development Goals (SDG), particularly SDG 3, Good Health and Wellbeing, and 10, Reduce Inequalities. Thus, our review will be disseminated at international conferences and will potentially contribute to formulating public policies to improve access to health care by slum dwellers.

The final manuscript will be submitted to an open-access international journal to reach a larger audience.

Contributions

The review team is distributed in three groups and participants were allocated to these groups according to their skills, experience in conducting systematic reviews and data analysis, and time availability. NTSF, HE, JL, EK, PPH, ZQ are part of the *core group* and are responsible for developing the main activities of the scoping review, such as screen and select the manuscripts, extract the data, and data analysis and writing the manuscript and reports. The second group comprises *new reviewers*. They will participate in the whole review process under the guidance and mentorship of the participants from the core group. AA, IM, FM, RK, SS, JS, VS, SG, and IC are part of the *new reviewer* group. The third group comprises the *advisory group* and includes senior researchers with expertise in health economics, urban health, and slums. They will guide the core team on the data analysis process, definitions of key-word, such as slums and economic impact, also acting as tie-breakers on the occasion of disagreements in the inclusion and data extraction stages of the review. BM, VR, RT, and SM are part of this group.

This protocol was written by NTSF and HE with contributions and approval from all co-authors.

Funding sources/sponsors: this review is supported by ARISE UK Research and Innovation's Global Challenges Research Fund (ARISE's Metrics, Epidemiology and Economics work package group/University of York). EK also acknowledges support from the Medical Research Council and Scottish Government Chief Scientist Office funding (MC_UU_12017/13, SPHSU13). The funders had or will have no role in the development of this protocol, the collection and analyses, or interpretation of results, or in the writing or publication of the review's results.

Competing interests: None declared.

For peer review only - http://bmjopen.bmj.com/site/about/guidelines.xhtml

Acknowledgment: We thank Melissa Harden, the information specialist form the Centre for Reviews and Dissemination at the University of York, for developing the search strategy.

Data availability: No data have been generated as this is a study protocol.

For peer review only

For peer review only - http://bmjopen.bmj.com/site/about/guidelines.xhtml

References

1. United Nations. World Urbanization Prospects. The 2018 revision. New York; 2019.

2. United Nations. The Millennium Development Goals Report [Internet]. United Nations. 2015. Available from: https://visit.un.org/millenniumgoals/2008highlevel/pdf/MDG_Report_2008_Addendum.pdf

3. The World Bank. World Bank Country and Lending Groups [Internet]. 2020 [cited 2020 Sep 22]. Available from: https://datahelpdesk.worldbank.org/knowledgebase/articles/906519

4. Chen M, Zhang H, Liu W, Zhang W. The Global Pattern of Urbanization and Economic Growth : Evidence from the Last Three Decades. Plos O. 2014;9(8).

5. Ezeh A, Oyebode O, Satterthwaite D, Chen YF, Ndugwa R, Sartori J, et al. The history, geography, and sociology of slums and the health problems of people who live in slums. Lancet [Internet]. 2017;389(10068):547–58. Available from: http://dx.doi.org/10.1016/S0140-6736(16)31650-6

6. Shawar YR, Crane LG. Generating global political priority for urban health : the role of the urban health epistemic community. 2017;(June):1161–73.

7. Mberu BU, Haregu TN, Kyobutungi C, Ezeh AC. Health and health-related indicators in slum , rural , and urban communities : a comparative analysis. 2016;1:1–13.

8. Fink G, Günther I, Hill K. Slum Residence and Child Health in Developing Countries. Demography. 2014;51:1175–1197.

9. National Institute of Population Research and Training. Bangladesh Urban Health Survey 2013. Final report. 2015.

10. Elsey H, Thomson DR, Lin RY, Maharjan U, Agarwal S, Newell J. Addressing Inequities in Urban Health : Do Decision-Makers Have the Data They Need ? Report from the Urban Health Data Special Session at International Conference on Urban Health Dhaka 2015. J Urban Heal [Internet]. 2016;93(3):526–37. Available from: http://dx.doi.org/10.1007/s11524-016-0046-9

11. Lucci P, Bhatkal T. Monitoring progress on urban poverty Are current data and indicators fit for. 2014;(October).

12. Patil RR. Social Work in Public Health Urbanization as a Determinant of Health : A Socioepidemiological Perspective Urbanization as a Determinant of Health : A Socioepidemiological Perspective. 2014;1918.

13. Kimani-Murage EW, Fotso JC, Egondi T, Abuya B, Elungata P, Ziraba AK, et al. Trends in childhood mortality in Kenya: The urban advantage has seemingly been wiped out. Heal Place [Internet]. 2014;29:95–103. Available from: http://dx.doi.org/10.1016/j.healthplace.2014.06.003

14. Fotso JC, Madise N, Baschieri A, Cleland J, Zulu E, Kavao Mutua M, et al. Child growth in urban deprived settings: Does household poverty status matter? At which stage of child development? Heal Place [Internet]. 2012;18(2):375–84. Available from: http://dx.doi.org/10.1016/j.healthplace.2011.12.003

15. Ogunsina MA, Anumah FO. Prevalence and correlates of hypertension and diabetes mellitus in an urban community in North-Western Nigeria. 2018;8688:1–7.

16. Mberu B, Wamukoya M, Oti S, Kyobutungi C. Trends in Causes of Adult Deaths among the Urban Poor : Evidence from Nairobi Urban Health and Demographic Trends in Causes of Adult

For peer review only - http://bmjopen.bmj.com/site/about/guidelines.xhtml

Deaths among the Urban Poor : Evidence from Nairobi Urban Health and Demographic Surveillance System , 2003 – 2012. J Urban Heal Urban Heal. 2015;(March):2003–12.

17. Govindaraj R, Raju D, Secci F, Chowdhury S, Frere J-J. Health and nutrition in Bangladesh: Social Determinants and Governance. World Bank Group; 2018.

18. Peters R, Amugsi DA, Mberu B, Ensor T, Hill AJ, Newell JN, et al. Nutrition transition , overweight and obesity among rural-to-urban migrant women in Kenya Nutrition transition , overweight and obesity among rural-to-urban migrant women in Kenya. 2019;(June).

19. Ofori-asenso R, Agyeman AA, Laar A, Boateng D. Overweight and obesity epidemic in Ghana — a systematic review and meta-analysis. BMC Public Health [Internet]. 2016; Available from: http://dx.doi.org/10.1186/s12889-016-3901-4

20. Williams CT, Grier SA, Marks AS. " Coming to Town " : The Impact of Urbanicity , Cigarette Advertising , and Network Norms on the Smoking Attitudes of Black Women in Cape Town , South Africa. 2008;85(4):472–85.

21. Rawal LB, Biswas T, Khandker NN, Saha SR, Chowdhury MMB, Khan ANS, et al. Non-communicable disease (NCD) risk factors and diabetes among adults living in slum areas of Dhaka, Bangladesh. PLoS One. 2017;12(10):1–15.

22. Gitatui M, Kimani S, Muniu S, Okube O, Kimani S. Determinants of harmful use of alcohol among urban slum dwelling adults in Kenya. 2019;19(4):2906–25.

23. Adhikari TB, Rijal A, Kallestrup P, Neupane D. Alcohol consumption pattern in western Nepal : findings from the COBIN baseline survey. 2019;(September).

24. Fisher M. A theory of public wellbeing. BMC Public Health. 2019;19(1):1–12.

25. Albis MLF, Bhadra SK, Chin B. Impact evaluation of contracting primary health care services in urban Bangladesh. BMC Health Serv Res [Internet]. 2019;854. Available from: http://dx.doi.org/10.1186/s12913-019-4406-5

26. Angeles G, Ahsan KZ. Reducing Inequity in Urban Health : Have the Intra-urban Differentials in Reproductive Health Service Utilization and Child Nutritional Outcome Narrowed in Bangladesh ? Reducing Inequity in Urban Health : Have the Intra-urban Differentials in Reproductive. 2018;(September).

27. Van Belle S, Affun-Adegbulu C, Soors W, Srinivas PN, Hegel G, Van Damme W, et al. COVID-19 and informal settlements: An urgent call to rethink urban governance. Int J Equity Health. 2020;19(1):1–2.

28. Bartlett JD, Vivrette R. Ways to Promote Children ' s Resilience to the COVID-19 Pandemic. 2020.

29. Mirzoev T, Poudel AN, Gissing S, Thuy T, Doan D, Ferdous T, et al. Is evidence-informed urban health planning a myth or reality ? Lessons from a qualitative assessment in three Asian cities. 2019;(October):773–83.

30. Otieno PO, Wambiya EOA, Mohamed SM, Mutua MK, Kibe PM, Mwangi B, et al. Access to primary healthcare services and associated factors in urban slums in. 2020;1–9.

31. Adams AM, Islam R, Yusuf SS, Panasci A, Crowell N. Healthcare seeking for chronic illness among adult slum dwellers in Bangladesh : A descriptive cross-sectional study in two urban settings. PLoS One [Internet]. 2020;1–18. Available from: http://dx.doi.org/10.1371/journal.pone.0233635

For peer review only

For peer review only - http://bmjopen.bmj.com/site/about/guidelines.xhtml

32. Buigut S, Ettarh R, Amendah DD. Catastrophic health expenditure and its determinants in Kenya slum communities. Int J Equity Health [Internet]. 2015;14(1):1–12. Available from: ???

33. Biswas S. India coronavirus: “More than half of Mumbai slum-dwellers had Covid-19” [Internet]. BBC News. 2020 [cited 2020 Sep 29]. Available from: https://www.bbc.co.uk/news/world-asia-india-53576653

34. The Guardian. Half of Mumbai’s slum residents have had coronavirus – study [Internet]. 2020 [cited 2020 Sep 29]. Available from: https://www.theguardian.com/world/2020/jul/29/half-of-mumbais-slum-residents-have-had-coronavirus-study

35. ARISE. ARISE, Accountability in Urban Health [Internet]. 2019 [cited 2020 Jun 6]. Available from: http://www.ariseconsortium.org/

36. Arksey H, O’Malley L. Scoping studies: Towards a methodological framework. Int J Soc Res Methodol Theory Pract. 2005;8(1):19–32.

37. Levac D, Colquhoun H, O’Brien KK. Scoping studies: Advancing the methodology. Implement Sci. 2010;5(1):1–9.

38. The Joanna Briggs Institute. The Systematic Review of Economic Evaluation Evidence [Internet]. The Joanna Briggs Institute; 2014. p. 1–40. Available from: www.joannabriggs.org

39. UN-Habitat. Slums : Some Definitions Slums : Neither Bricks nor Mortar , Non-Durable Housing [Internet]. United Nations; 2007. p. 3–4. Available from: https://mirror.unhabitat.org/documents/media_centre/sowcr2006/SOWCR 5.pdf

40. Xu K, Evans DB, Kawabata K, Zeramdini R, Klavus J, Murray CJL. Household catastrophic health expenditure : a multicountry analysis. Lancet. 2003;362:111–7.

41. Kirch W, editor. Direct Costs. In: Encyclopedia of Public Health [Internet]. Dordrecht: Springer Netherlands; 2008. p. 267. Available from: https://doi.org/10.1007/978-1-4020-5614-7_799

42. Tricco AC, Lillie E, Zarin W, O’Brien KK, Colquhoun H, Levac D, et al. PRISMA extension for scoping reviews (PRISMA-ScR): Checklist and explanation. Ann Intern Med. 2018;169(7):467–73.

43. Ouzzani M, Hammady H, Fedorowicz Z, Elmagarmid A. Rayyan-a web and mobile app for systematic reviews. Syst Rev. 2016;5(1):1–10.

44. Husereau D, Drummond M, Petrou S, Carswell C, Moher D, Greenberg D. Consolidated Health Economic Evaluation Reporting Standards (CHEERS)--Explanation and elaboration: A report of the ISPOR Health Economic Evaluations Publication Guidelines Task Force. Value Heal. 2013;16:231–50.

45. World Health Organization. The tool to estimate patients’ costs. 2008.

46. International Monetary Fund. International Monetary Fund [Internet]. 2020. Available from: https://www.imf.org/en/Data

47. Kirch W, editor. Purchasing Power Parity. In: Encyclopedia of Public Health [Internet]. Dordrecht: Springer Netherlands; 2008. p. 1220. Available from: https://doi.org/10.1007/978-1-4020-5614-7_2880

48. The World Bank. World Development Indicators: Exchange rates and prices [Internet]. 2017 [cited 2016 Nov 1]. Available from: http://wdi.worldbank.org/table/4.16

For peer review only

For peer review only - http://bmjopen.bmj.com/site/about/guidelines.xhtml

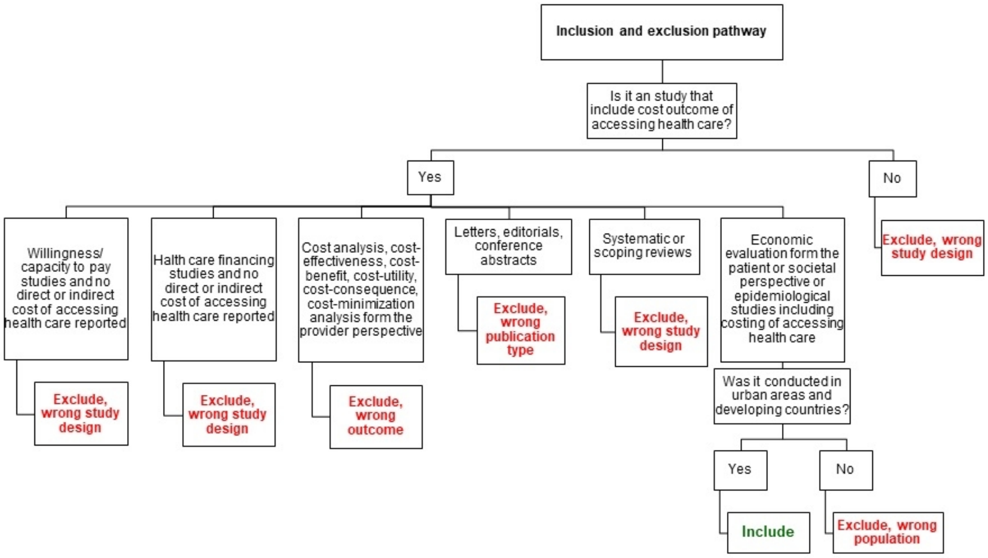

Figure 1. Inclusion and exclusion pathway of the scoping review.

235x145mm (96 x 96 DPI)

Supplementary material

The economics of health-care for slum-dwellers

Literature search

The aim of the search strategy was to identify studies examining the costs of healthcare for slum dwellers or city residents in low- and middle-income countries (LMIC). A search strategy was developed by an information specialist using Ovid MEDLINE. Search terms were gathered together for the following four key concepts: slum dwellers, urban areas, healthcare costs and LMIC. Both textword and subject heading searches for each concept were included in the strategy. Retrieval was limited to publications within the last 10 years. No langauage restrictions were applied. The final search strategy for MEDLINE was agreed by the review team and then translated as appropriate for the other databases and resources.

Databases containing literature from the fields of health, economics and social science were searched on 22nd June 2020: MEDLINE (Ovid), Embase (Ovid), EconLit (Ovid), Science Citation Index (Web of Science), Social Science Citation Index (Web of Science) and Global Index Medicus. In addition, the following resources containing grey literature such as theses and dissertations, working papers and reports were searched: Proquest Dissertations and Theses (A&I), Econpapers, OpenGrey, the World Bank website and the Organisation for Economic Co-operation and Development (OECD) website.

EndNote was used for reference management and duplicate removal. The search strategies for all databases and resources can be found in the appendix.

For peer review only

Appendix

The search strategies for MEDLINE and EMBASE incorporated a low- and middle-income countries (LMIC) search filter developed by the Effective Practice and Organisation of Care (EPOC) Cochrane group.[1] Where possible, this filter was translated for use in the other databases searched.

MEDLINE ALL
via Ovid http://ovidsp.ovid.com/
1946 to June 19, 2020
Search date: 22nd June 2020
Records retrieved: 2368

1 Urban Population/ (58856)
2 Cities/ (19180)
3 Urban Health/ (17793)
4 Urban Health Services/ (3613)
5 Hospitals, Urban/ (7341)
6 urban$.ti,ab. (156001)
7 (city or cities or megacity or megacities or metropolitan).ti,ab. (169349)
8 (metropolis or megalopolis or municipal$ or conurbation$ or suburb$ or town or towns or township$ or borough$ or barrio or barrios).ti,ab. (81323)
9 ((dense$ or density or high$ or large$ or heavy or heavily) adj3 (populat$ or populous) adj3 (area$ or settlement$ or district$ or neighbourhood$ or communit$)).ti,ab. (3451)
10 or/1-9 (390327)
11 (afghanistan or albania or algeria or american samoa or angola or "antigua and barbuda" or antigua or barbuda or argentina or armenia or armenian or aruba or azerbaijan or bahrain or bangladesh or barbados or republic of belarus or belarus or byelarus or belorussia or byelorussian or belize or british honduras or benin or dahomey or bhutan or bolivia or "bosnia and herzegovina" or bosnia or herzegovina or botswana or bechuanaland or brazil or brasil or bulgaria or burkina faso or burkina fasso or upper volta or burundi or urundi or cabo verde or cape verde or cambodia or kampuchea or khmer republic or cameroon or cameron or cameroun or central african republic or ubangi shari or chad or chile or china or colombia or comoros or comoro islands or iles comores or mayotte or democratic republic of the congo or democratic republic congo or congo or zaire or costa rica or "cote d'ivoire" or "cote d' ivoire" or cote divoire or cote d ivoire or ivory coast or croatia or cuba or cyprus or czech republic or czechoslovakia or djibouti or french somaliland or dominica or dominican republic or ecuador or egypt or united arab republic or el salvador or equatorial guinea or spanish guinea or eritrea or estonia or eswatini or swaziland or ethiopia or fiji or gabon or gabonese republic or gambia or "georgia (republic)" or georgian or ghana or gold coast or gibraltar or greece or grenada or guam or guatemala or guinea or guinea bissau or guyana or british guiana or haiti or hispaniola or honduras or hungary or india or indonesia or timor or iran or iraq or isle of man or jamaica or jordan or kazakhstan or kazakh or kenya or "democratic people's republic of korea" or republic of korea or north korea or south korea or korea or kosovo or kyrgyzstan or kirghizia or kirgizstan or kyrgyz republic or kirghiz or laos or lao pdr or "lao people's democratic republic" or latvia or lebanon or lebanese republic or lesotho or basutoland or liberia or libya or libyan arab jamahiriya or lithuania or macau or macao or "macedonia (republic)" or macedonia or madagascar or malagasy republic or malawi or nyasaland or malaysia or malay federation or malaya federation or maldives or indian ocean islands or indian ocean or mali or malta or micronesia or federated states of micronesia or kiribati or marshall islands or nauru or northern mariana islands or palau or tuvalu or mauritania or mauritius or mexico or moldova or moldovian or mongolia or montenegro or morocco or ifni or mozambique or portuguese east africa or myanmar or burma or namibia or nepal or netherlands antilles or nicaragua or niger or nigeria or oman or muscat or pakistan or panama or

For peer review only

papua new guinea or new guinea or paraguay or peru or philippines or philipines or phillipines or phillippines or poland or "polish people's republic" or portugal or portuguese republic or puerto rico or romania or russia or russian federation or ussr or soviet union or union of soviet socialist republics or rwanda or ruanda or samoa or pacific islands or polynesia or samoan islands or navigator island or navigator islands or "sao tome and principe" or saudi arabia or senegal or serbia or seychelles or sierra leone or slovakia or slovak republic or slovenia or melanesia or solomon island or solomon islands or norfolk island or norfolk islands or somalia or south africa or south sudan or sri lanka or ceylon or "saint kitts and nevis" or "st. kitts and nevis" or saint lucia or "st. lucia" or "saint vincent and the grenadines" or saint vincent or "st. vincent" or grenadines or sudan or suriname or surinam or dutch guiana or netherlands guiana or syria or syrian arab republic or tajikistan or tadjikistan or tadzhikistan or tadzhik or tanzania or tanganyika or thailand or siam or timor leste or east timor or togo or togolese republic or tonga or "trinidad and tobago" or trinidad or tobago or tunisia or turkey or "turkey (republic)" or turkmenistan or turkmen or uganda or ukraine or uruguay or uzbekistan or uzbek or vanuatu or new hebrides or venezuela or vietnam or viet nam or middle east or west bank or gaza or palestine or yemen or yugoslavia or zambia or zimbabwe or northern rhodesia or global south or africa south of the sahara or sub-saharan africa or subsaharan africa or africa, central or central africa or africa, northern or north africa or northern africa or magreb or maghrib or sahara or africa, southern or southern africa or africa, eastern or east africa or eastern africa or africa, western or west africa or western africa or west indies or indian ocean islands or caribbean or central america or latin america or "south and central america" or south america or asia, central or central asia or asia, northern or north asia or northern asia or asia, southeastern or southeastern asia or south eastern asia or southeast asia or south east asia or asia, western or western asia or europe, eastern or east europe or eastern europe or developing country or developing countries or developing nation? or developing population? or developing world or less developed countr* or less developed nation? or less developed population? or less developed world or lesser developed countr* or lesser developed nation? or lesser developed population? or lesser developed world or under developed countr* or under developed nation? or under developed population? or under developed world or underdeveloped countr* or underdeveloped nation? or underdeveloped population? or underdeveloped world or middle income countr* or middle income nation? or middle income population? or low income countr* or low income nation? or low income population? or lower income countr* or lower income nation? or lower income population? or underserved countr* or underserved nation? or underserved population? or underserved world or under served countr* or under served nation? or under served population? or under served world or deprived countr* or deprived nation? or deprived population? or deprived world or poor countr* or poor nation? or poor population? or poor world or poorer countr* or poorer nation? or poorer population? or poorer world or developing economy* or developing economies or less developed econom* or lesser developed econom* or under developed econom* or underdeveloped econom* or middle income econom* or low income econom* or lower income econom* or low gdp or low gnp or low gross domestic or low gross national or lower gdp or lower gnp or lower gross domestic or lower gross national or lmic or lmics or third world or lami countr* or transitional countr* or emerging economy or emerging economies or emerging nation?).ti,ab,sh,kf. (1907989)

12 (afghan or afghans or afghani or albanian? algerian? or american samoan? or angolan? or antiguan? or barbudan? or argentine? or argentinian? or argentinean? or armenian? or aruban? or azerbaijani? or bahraini? or bangladeshi? or bangalees or bajan? or belarusian? or byelorussian? or belizean? or beninese? or bhutanese or bolivian? or bosnian? or botswana or batswana or brazilian? or brasilian? or bulgarian? or burkinabe or burkinese or burundian? or cape verdean? or cabo verdean? or cambodian? or khmer or cameroonian? or central african? or chadian? or chilean? or chinese or colombian? or comorian? or congolese or costa rican? or ivorian? or croatian? or cuban? or cypriot? or czech? or djiboutian? or dominican? or ecuadorian? or egyptian? or salvadoran? or equatorial guinean? or equatoguinean? or eritrean? or estonian? or swazi? or swati? or ethiopian? or fijian or gabonese or gabonaise or gambian? or georgian? or ghanaian? or gibraltarian? or greek?

For peer review only

or grenadian? or guamanian? or guatemalan? or guinean? or bissau guinean? or guyanese or haitian? or honduran? or hungarian? or indian? or indonesian? or iranian? or iraqian? or iraqi? or manx or jamaican? or jordanian? or kazakhstani? or kenyan? or kirabati or kirabatian? or north korean? or korean? or kosovar? or kosovan? or kyrgyz* or lao or laotian? or latvian? or lebanese or lesothan? or lesothonian? or mosotho or basotho or liberian? or libyan? or lithuanian? or macanese or macedonian? or malagasy or madagascan? or malawian? or malaysian? or maldivian? or malian? or maltese or marshallese? or mauritanian? or mauritian? or mexican? or micronesian? or moldovan? or mongolian? or mongol or montenegrin? or moroccan? or mozambican? or burmese or myanma or namibian? or nauruan? or nepali or nepalese or netherlands antillean? or nicaraguan? or nigerien? or nigerian? or northern mariana islander? or mariana? or omani? or pakistani? or palauan? or panamanian? or papua new guinean? or paraguayan? or peruvian? or philippine? or philipine? or phillipine? or phillippine? or filipino? or filipina? or polish or pole or poles or portuguese or puerto rican? or romanian? or russian? or soviet people or soviet population or rwandan? or rwandese or ruandan? or ruandese or samoan? or sao tomean? or santomean? or saudi arabian? or saudi? or senegalese or serbian? or montenegrin? or seychellois or seychelloise? or sierra leonean? or slovak? or slovene? or solomon islander? or somali? or south african? or south sudanese or sri lankan? or ceylonese or kittitian? or nevisian? or saint lucian? or vincentian? or sudanese or surinamese? or syrian? or tajik? or tajikistani? or tanzanian? or tanganyikan? or thai or timorese? or togolese or tongan? or trinidadian? or tobagonian? or tunisian? or turk? or turkish or turkmen? or tuvaluan? or ugandan? or ukrainian? or uruguayan? or uzbek? or vanuatu* or venezuelan? or vietnamese or yemeni? or yemenite? or yemenese or yugoslav? or yugoslavian? or zambian? or zimbabwean?).ti,ab,sh,kf. (834927)
13 11 or 12 (2269396)
14 10 and 13 (169934)
15 poverty areas/ (6047)
16 (slum or slums or ghetto or ghettos or shanty$ or shanties or shack$ or favela$).ti,ab. (5200)
17 (informal$ adj3 settlement$).ti,ab. (558)
18 ((poverty or impoverish$) adj3 (area$ or settlement$)).ti,ab. (881)
19 ((precarious$ or irregular$) adj3 settlement$).ti,ab. (8)
20 (squatter$ adj3 (area$ or settlement$)).ti,ab. (227)
21 or/15-20 (11156)
22 "cost of illness"/ (27054)
23 exp Health Care Costs/ (64997)
24 Health Expenditures/ (20173)
25 exp Financing, Personal/ (6053)
26 Catastrophic Illness/ec [Economics] (550)
27 (cost$ adj2 (illness$ or sickness$ or disease$)).ti,ab. (5084)
28 (cost$ adj3 (care or health or healthcare or medical$)).ti,ab. (76646)
29 (expenditure$ adj3 (care or health or healthcare or medical$)).ti,ab. (13957)
30 ((direct or indirect) adj2 (cost$ or expenditure$)).ti,ab. (15577)
31 out of pocket.ti,ab. (5698)
32 (OOP adj3 (cost$ or expense$ or expenditure$ or financ$ or pay$ or paid or spend$)).ti,ab. (505)
33 OOPE.ti,ab. (74)
34 ((personal or individual$ or patient$ or family$ or families or household$) adj3 (budget$ or cost$ or expense$ or expenditure$ or financ$ or pay$ or paid or spend$) adj3 (care or health or healthcare or medical$)).ti,ab. (9512)
35 (catastroph$ adj10 (cost$ or expense$ or expenditure$ or financ$ or pay$ or paid or spend$)).ti,ab. (1323)
36 ((medical$ or health) adj3 impoverish$).ti,ab. (232)

For peer review only

37 (burden$ adj3 (cost$ or expenditure$ or economic$ or financ$) adj3 (care or health or healthcare or medical$)).ti,ab. (4485)
38 ((willing$ or unwilling$) adj3 pay$ adj3 (care or health or healthcare or medical$)).ti,ab. (323)
39 ((afford$ or unafford$) adj5 (cost$ or pay$)).ti,ab. (2273)
40 ((able or ability or capacit$ or unable or inability) adj2 pay$).ti,ab. (1460)
41 or/22-40 (185296)
42 14 and 41 (3361)
43 21 and 41 (267)
44 42 or 43 (3541)
45 letter.pt. (1084673)
46 editorial.pt. (532516)
47 historical article.pt. (358723)
48 or/45-47 (1956361)
49 44 not 48 (3506)
50 exp animals/ not humans/ (4709143)
51 49 not 50 (3497)
52 limit 51 to yr="2010 -Current" (2368)

Key:
/ = subject heading (MeSH heading)
exp = exploded subject heading (MeSH heading)
/ec = economics subheading
$ = truncation
* = truncation
" " = exact phrase search
? = stands for zero or one characters within a word or at the end of a word
ti,ab = terms in either title or abstract fields
kf = terms in author keywords field
sh = terms in the subject heading field
pt = terms in publication type field
adj3 = terms within three words of each other (any order)
adj = terms next to each other in the same order

Embase
via Ovid http://ovidsp.ovid.com/
1974 to 2020 June 19
Search date: 22nd June 2020
Records retrieved: 2958

1 urban area/ (60321)
2 urban population/ (44806)
3 urban health/ (1037)
4 urban hospital/ (303)
5 city/ (38692)
6 suburban area/ (2548)
7 suburban population/ (670)
8 (city or cities or megacity or megacities or metropolitan).ti,ab. (219736)
9 (metropolis or megalopolis or municipal$ or conurbation$ or suburb$ or town or towns or township$ or borough$ or barrio or barrios).ti,ab. (100040)
10 ((dense$ or density or high$ or large$ or heavy or heavily) adj3 (populat$ or populous) adj3 (area$ or settlement$ or district$ or neighbourhood$ or communit$)).ti,ab. (4243)

For peer review only

11 urban$.ti,ab. (200356)
12 or/1-11 (486662)
13 (afghanistan or albania or algeria or american samoa or angola or "antigua and barbuda" or antigua or barbuda or argentina or armenia or armenian or aruba or azerbaijan or bahrain or bangladesh or barbados or republic of belarus or belarus or byelarus or belorussia or byelorussian or belize or british honduras or benin or dahomey or bhutan or bolivia or "bosnia and herzegovina" or bosnia or herzegovina or botswana or bechuanaland or brazil or brasil or bulgaria or burkina faso or burkina fasso or upper volta or burundi or urundi or cabo verde or cape verde or cambodia or kampuchea or khmer republic or cameroon or cameron or cameroun or central african republic or ubangi shari or chad or chile or china or colombia or comoros or comoro islands or iles comores or mayotte or democratic republic of the congo or democratic republic congo or congo or zaire or costa rica or "cote d'ivoire" or "cote d' ivoire" or cote divoire or cote d ivoire or ivory coast or croatia or cuba or cyprus or czech republic or czechoslovakia or djibouti or french somaliland or dominica or dominican republic or ecuador or egypt or united arab republic or el salvador or equatorial guinea or spanish guinea or eritrea or estonia or eswatini or swaziland or ethiopia or fiji or gabon or gabonese republic or gambia or "georgia (republic)" or georgian or ghana or gold coast or gibraltar or greece or grenada or guam or guatemala or guinea or guinea bissau or guyana or british guiana or haiti or hispaniola or honduras or hungary or india or indonesia or timor or iran or iraq or isle of man or jamaica or jordan or kazakhstan or kazakh or kenya or "democratic people's republic of korea" or republic of korea or north korea or south korea or korea or kosovo or kyrgyzstan or kirghizia or kirgizstan or kyrgyz republic or kirghiz or laos or lao pdr or "lao people's democratic republic" or latvia or lebanon or lebanese republic or lesotho or basutoland or liberia or libya or libyan arab jamahiriya or lithuania or macau or macao or "macedonia (republic)" or macedonia or madagascar or malagasy republic or malawi or nyasaland or malaysia or malay federation or malaya federation or maldives or indian ocean islands or indian ocean or mali or malta or micronesia or federated states of micronesia or kiribati or marshall islands or nauru or northern mariana islands or palau or tuvalu or mauritania or mauritius or mexico or moldova or moldovian or mongolia or montenegro or "montenegro (republic)" or morocco or ifni or mozambique or portuguese east africa or myanmar or burma or namibia or nepal or netherlands antilles or nicaragua or niger or nigeria or oman or muscat or pakistan or panama or papua new guinea or new guinea or paraguay or peru or philippines or philipines or phillipines or phillippines or poland or "polish people's republic" or portugal or portuguese republic or puerto rico or romania or russia or russian federation or ussr or soviet union or union of soviet socialist republics or rwanda or ruanda or samoa or pacific islands or polynesia or samoan islands or navigator island or navigator islands or "sao tome and principe" or saudi arabia or senegal or serbia or seychelles or sierra leone or slovakia or slovak republic or slovenia or melanesia or solomon island or solomon islands or norfolk island or norfolk islands or somalia or south africa or south sudan or sri lanka or ceylon or "saint kitts and nevis" or "st. kitts and nevis" or saint lucia or "st. lucia" or "saint vincent and the grenadines" or saint vincent or "st. vincent" or grenadines or sudan or suriname or surinam or dutch guiana or netherlands guiana or syria or syrian arab republic or tajikistan or tadjikistan or tadzhikistan or tadzhik or tanzania or tanganyika or thailand or siam or timor leste or east timor or togo or togolese republic or tonga or "trinidad and tobago" or trinidad or tobago or tunisia or turkey or "turkey (republic)" or turkmenistan or turkmen or uganda or ukraine or uruguay or uzbekistan or uzbek or vanuatu or new hebrides or venezuela or vietnam or viet nam or middle east or west bank or gaza or palestine or yemen or yugoslavia or zambia or zimbabwe or northern rhodesia or global south or africa south of the sahara or "sub saharan africa" or subsaharan africa or africa, central or central africa or africa, northern or north africa or northern africa or magreb or maghrib or sahara or africa, southern or southern africa or africa, eastern or east africa or eastern africa or africa, western or west africa or western africa or west indies or indian ocean islands or caribbean region or caribbean islands or caribbean or central america or latin america or "south and central america" or south america or asia, central or central asia or asia, northern or north asia or northern asia or asia, southeastern or southeastern asia or south eastern asia or

For peer review only

southeast asia or south east asia or asia, western or western asia or europe, eastern or east europe or eastern europe or developing country or developing countries or developing nation? or developing population? or developing world or less developed countr* or less developed nation? or less developed population? or less developed world or lesser developed countr* or lesser developed nation? or lesser developed population? or lesser developed world or under developed countr* or under developed nation? or under developed population? or under developed world or underdeveloped countr* or underdeveloped nation? or underdeveloped population? or underdeveloped world or middle income countr* or middle income nation? or middle income population? or low income countr* or low income nation? or low income population? or lower income countr* or lower income nation? or lower income population? or underserved countr* or underserved nation? or underserved population? or underserved world or under served countr* or under served nation? or under served population? or under served world or deprived countr* or deprived nation? or deprived population? or deprived world or poor countr* or poor nation? or poor population? or poor world or poorer countr* or poorer nation? or poorer population? or poorer world or developing econom* or less developed econom* or lesser developed econom* or under developed econom* or underdeveloped econom* or middle income econom* or low income econom* or lower income econom* or low gdp or low gnp or low gross domestic or low gross national or lower gdp or lower gnp or lower gross domestic or lower gross national or lmic or lmics or third world or lami countr* or transitional countr* or emerging economies or emerging economy or emerging nation?).ti,ab,sh,kw. (2145013)

14 (afghan or afghans or afghani or albanian? algerian? or american samoan? or angolan? or antiguan? or barbudan? or argentine? or argentinian? or argentinean? or armenian? or aruban? or azerbaijani? or bahraini? or bangladeshi? or bangalees or bajan? or belarusian? or byelorussian? or belizean? or beninese? or bhutanese or bolivian? or bosnian? or botswana or batswana or brazilian? or brasilian? or bulgarian? or burkinabe or burkinese or burundian? or cape verdean? or cabo verdean? or cambodian? or khmer or cameroonian? or central african? or chadian? or chilean? or chinese or colombian? or comorian? or congolese or costa rican? or ivorian? or croatian? or cuban? or cypriot? or czech? or djiboutian? or dominican? or ecuadorian? or egyptian? or salvadoran? or equatorial guinean? or equatoguinean? or eritrean? or estonian? or swazi? or swati? or ethiopian? or fijian or gabonese or gabonaise or gambian? or georgian? or ghanaian? or gibraltarian? or greek? or grenadian? or guamanian? or guatemalan? or guinean? or bissau guinean? or guyanese or haitian? or honduran? or hungarian? or indian? or indonesian? or iranian? or iraqian? or iraqi? or manx or jamaican? or jordanian? or kazakhstani? or kenyan? or kirabati or kirabatian? or north korean? or korean? or kosovar? or kosovan? or kyrgyz* or lao or laotian? or latvian? or lebanese or lesothan? or lesothonian? or mosotho or basotho or liberian? or libyan? or lithuanian? or macanese or macedonian? or malagasy or madagascan? or malawian? or malaysian? or maldivian? or malian? or maltese or marshallese? or mauritanian? or mauritian? or mexican? or micronesian? or moldovan? or mongolian? or mongol or montenegrin? or moroccan? or mozambican? or burmese or myanma or namibian? or nauruan? or nepali or nepalese or netherlands antillean? or nicaraguan? or nigerien? or nigerian? or northern mariana islander? or mariana? or omani? or pakistani? or palauan? or panamanian? or papua new guinean? or paraguayan? or peruvian? or philippine? or philipine? or phillipine? or phillippine? or filipino? or filipina? or polish or pole or poles or portuguese or puerto rican? or romanian? or russian? or soviet people or soviet population or rwandan? or rwandese or ruandan? or ruandese or samoan? or sao tomean? or santomean? or saudi arabian? or saudi? or senegalese or serbian? or montenegrin? or seychellois or seychelloise? or sierra leonean? or slovak? or slovene? or solomon islander? or somali? or south african? or south sudanese or sri lankan? or ceylonese or kittitian? or nevisian? or saint lucian? or vincentian? or sudanese or surinamese? or syrian? or tajik? or tajikistani? or tanzanian? or tanganyikan? or thai or timorese? or togolese or tongan? or trinidadian? or tobagonian? or tunisian? or turk? or turkish or turkmen? or tuvaluan? or ugandan? or ukrainian? or uruguayan? or uzbek? or vanuatu* or

For peer review only

venezuelan? or vietnamese or yemeni? or yemenite? or yemenese or yugoslav? or yugoslavian? or zambian? or zimbabwean?).ti,ab,sh,kw. (1119374)
15 13 or 14 (2730608)
16 12 and 15 (203906)
17 (slum or slums or ghetto or ghettos or shanty$ or shanties or shack$ or favela$).mp. (5658)
18 (informal$ adj3 settlement$).mp. (628)
19 ((poverty or impoverish$) adj3 (area$ or settlement$)).mp. (1075)
20 ((precarious$ or irregular$) adj3 settlement$).mp. (12)
21 (squatter$ adj3 (area$ or settlement$)).mp. (207)
22 or/17-21 (7364)
23 "cost of illness"/ (19139)
24 exp "health care cost"/ (289949)
25 health economics/ (32736)
26 willingness to pay/ (695)
27 catastrophic health expenditure/ (32)
28 out of pocket/ (3)
29 (cost$ adj2 (illness$ or sickness$ or disease$)).ti,ab. (7680)
30 (cost$ adj3 (care or health or healthcare or medical$)).ti,ab. (114334)
31 (expenditure$ adj3 (care or health or healthcare or medical$)).ti,ab. (18625)
32 ((direct or indirect) adj2 (cost$ or expenditure$)).ti,ab. (26279)
33 out of pocket.ti,ab. (8054)
34 (OOP adj3 (cost$ or expense$ or expenditure$ or financ$ or pay$ or paid or spend$)).ti,ab. (797)
35 OOPE.ti,ab. (95)
36 ((personal or individual$ or patient$ or family$ or families or household$) adj3 (budget$ or cost$ or expense$ or expenditure$ or financ$ or pay$ or paid or spend$) adj3 (care or health or healthcare or medical$)).ti,ab. (14828)
37 (catastroph$ adj10 (cost$ or expense$ or expenditure$ or financ$ or pay$ or paid or spend$)).ti,ab. (1521)
38 ((medical$ or health) adj3 impoverish$).ti,ab. (241)
39 (burden$ adj3 (cost$ or expenditure$ or economic$ or financ$) adj3 (care or health or healthcare or medical$)).ti,ab. (6941)
40 ((willing$ or unwilling$) adj3 pay$ adj3 (care or health or healthcare or medical$)).ti,ab. (458)
41 ((afford$ or unafford$) adj5 (cost$ or pay$)).ti,ab. (3175)
42 ((able or ability or capacit$ or unable or inability) adj2 pay$).ti,ab. (1801)
43 or/23-42 (414755)
44 16 and 43 (5138)
45 22 and 43 (202)
46 44 or 45 (5237)
47 letter.pt. (1118273)
48 editorial.pt. (655452)
49 47 or 48 (1773725)
50 46 not 49 (5179)
51 limit 50 to conference abstracts (970)
52 50 not 51 (4209)
53 limit 52 to yr="2010 -Current" (2958)

Key:
/ = subject heading (EMTREE term)
exp = exploded subject heading (EMTREE term)
$ = truncation

For peer review only

* = truncation
“ “ = exact phrase search
? = stands for zero or one characters within a word or at the end of a word
ti,ab = terms in either title or abstract fields
mp = multi-purpose fields search – includes title, abstract, author keywords, or subject headings field
kw = terms in author keywords field
sh = terms in the subject heading field
pt = terms in publication type field
adj3 = terms within three words of each other (any order)
adj = terms next to each other in the same order

EconLit
via Ovid http://ovidsp.ovid.com/
1886 to June 11 2020
Search date: 22nd June 2020
Records retrieved: 197

1 (city or cities or megacity or megacities or metropolitan).ti,ab. (35238)
2 (metropolis or megalopolis or municipal$ or conurbation$ or suburb$ or town or towns or township$ or borough$ or barrio or barrios).ti,ab. (15322)
3 ((dense$ or density or high$ or large$ or heavy or heavily) adj3 (populat$ or populous) adj3 (area$ or settlement$ or district$ or neighbourhood$ or communit$)).ti,ab. (356)
4 urban$.ti,ab. (36202)
5 or/1-4 (69848)
6 (afghanistan or albania or algeria or american samoa or angola or "antigua and barbuda" or antigua or barbuda or argentina or armenia or armenian or aruba or azerbaijan or bahrain or bangladesh or barbados or republic of belarus or belarus or byelarus or belorussia or byelorussian or belize or british honduras or benin or dahomey or bhutan or bolivia or "bosnia and herzegovina" or bosnia or herzegovina or botswana or bechuanaland or brazil or brasil or bulgaria or burkina faso or burkina fasso or upper volta or burundi or urundi or cabo verde or cape verde or cambodia or kampuchea or khmer republic or cameroon or cameron or cameroun or central african republic or ubangi shari or chad or chile or china or colombia or comoros or comoro islands or iles comores or mayotte or democratic republic of the congo or democratic republic congo or congo or zaire or costa rica or "cote d’ivoire" or "cote d’ ivoire" or cote divoire or cote d ivoire or ivory coast or croatia or cuba or cyprus or czech republic or czechoslovakia or djibouti or french somaliland or dominica or dominican republic or ecuador or egypt or united arab republic or el salvador or equatorial guinea or spanish guinea or eritrea or estonia or eswatini or swaziland or ethiopia or fiji or gabon or gabonese republic or gambia or "georgia (republic)" or georgian or ghana or gold coast or gibraltar or greece or grenada or guam or guatemala or guinea or guinea bissau or guyana or british guiana or haiti or hispaniola or honduras or hungary or india or indonesia or timor or iran or iraq or isle of man or jamaica or jordan or kazakhstan or kazakh or kenya or "democratic people’s republic of korea" or republic of korea or north korea or south korea or korea or kosovo or kyrgyzstan or kirghizia or kirgizstan or kyrgyz republic or kirghiz or laos or lao pdr or "lao people's democratic republic" or latvia or lebanon or lebanese republic or lesotho or basutoland or liberia or libya or libyan arab jamahiriya or lithuania or macau or macao or "macedonia (republic)" or macedonia or madagascar or malagasy republic or malawi or nyasaland or malaysia or malay federation or malaya federation or maldives or indian ocean islands or indian ocean or mali or malta or micronesia or federated states of micronesia or kiribati or marshall islands or nauru or northern mariana islands or palau or tuvalu or mauritania or mauritius or mexico or moldova or moldovian or mongolia or montenegro or morocco or ifni or mozambique or portuguese east africa or myanmar or burma or namibia or nepal

For peer review only

or netherlands antilles or nicaragua or niger or nigeria or oman or muscat or pakistan or panama or papua new guinea or new guinea or paraguay or peru or philippines or philipines or phillipines or phillippines or poland or "polish people's republic" or portugal or portuguese republic or puerto rico or romania or russia or russian federation or ussr or soviet union or union of soviet socialist republics or rwanda or ruanda or samoa or pacific islands or polynesia or samoan islands or navigator island or navigator islands or "sao tome and principe" or saudi arabia or senegal or serbia or seychelles or sierra leone or slovakia or slovak republic or slovenia or melanesia or solomon island or solomon islands or norfolk island or norfolk islands or somalia or south africa or south sudan or sri lanka or ceylon or "saint kitts and nevis" or "st. kitts and nevis" or saint lucia or "st. lucia" or "saint vincent and the grenadines" or saint vincent or "st. vincent" or grenadines or sudan or suriname or surinam or dutch guiana or netherlands guiana or syria or syrian arab republic or tajikistan or tadjikistan or tadzhikistan or tadzhik or tanzania or tanganyika or thailand or siam or timor leste or east timor or togo or togolese republic or tonga or "trinidad and tobago" or trinidad or tobago or tunisia or turkey or "turkey (republic)" or turkmenistan or turkmen or uganda or ukraine or uruguay or uzbekistan or uzbek or vanuatu or new hebrides or venezuela or vietnam or viet nam or middle east or west bank or gaza or palestine or yemen or yugoslavia or zambia or zimbabwe or northern rhodesia or global south or africa south of the sahara or sub-saharan africa or subsaharan africa or africa, central or central africa or africa, northern or north africa or northern africa or magreb or maghrib or sahara or africa, southern or southern africa or africa, eastern or east africa or eastern africa or africa, western or west africa or western africa or west indies or indian ocean islands or caribbean or central america or latin america or "south and central america" or south america or asia, central or central asia or asia, northern or north asia or northern asia or asia, southeastern or southeastern asia or south eastern asia or southeast asia or south east asia or asia, western or western asia or europe, eastern or east europe or eastern europe or developing country or developing countries or developing nation? or developing population? or developing world or less developed countr* or less developed nation? or less developed population? or less developed world or lesser developed countr* or lesser developed nation? or lesser developed population? or lesser developed world or under developed countr* or under developed nation? or under developed population? or under developed world or underdeveloped countr* or underdeveloped nation? or underdeveloped population? or underdeveloped world or middle income countr* or middle income nation? or middle income population? or low income countr* or low income nation? or low income population? or lower income countr* or lower income nation? or lower income population? or underserved countr* or underserved nation? or underserved population? or underserved world or under served countr* or under served nation? or under served population? or under served world or deprived countr* or deprived nation? or deprived population? or deprived world or poor countr* or poor nation? or poor population? or poor world or poorer countr* or poorer nation? or poorer population? or poorer world or developing econom* or less developed econom* or lesser developed econom* or under developed econom* or underdeveloped econom* or middle income econom* or low income econom* or lower income econom* or low gdp or low gnp or low gross domestic or low gross national or lower gdp or lower gnp or lower gross domestic or lower gross national or lmic or lmics or third world or lami countr* or transitional countr* or emerging economies or emerging economy or emerging nation?).ti,ab,sh,kw. (270638)

7 (afghan or afghans or afghani or albanian? algerian? or american samoan? or angolan? or antiguan? or barbudan? or argentine? or argentinian? or argentinean? or armenian? or aruban? or azerbaijani? or bahraini? or bangladeshi? or bangalees or bajan? or belarusian? or byelorussian? or belizean? or beninese? or bhutanese or bolivian? or bosnian? or botswana or batswana or brazilian? or brasilian? or bulgarian? or burkinabe or burkinese or burundian? or cape verdean? or cabo verdean? or cambodian? or khmer or cameroonian? or central african? or chadian? or chilean? or chinese or colombian? or comorian? or congolese or costa rican? or ivorian? or croatian? or cuban? or cypriot? or czech? or djiboutian? or dominican? or ecuadorian? or egyptian? or salvadoran? or equatorial guinean? or equatoguinean? or eritrean? or estonian? or swazi? or swati? or ethiopian?

For peer review only - http://bmjopen.bmj.com/site/about/guidelines.xhtml

or fijian or gabonese or gabonaise or gambian? or georgian? or ghanaian? or gibraltarian? or greek? or grenadian? or guamanian? or guatemalan? or guinean? or bissau guinean? or guyanese or haitian? or honduran? or hungarian? or indian? or indonesian? or iranian? or iraqian? or iraqi? or manx or jamaican? or jordanian? or kazakhstani? or kenyan? or kirabati or kirabatian? or north korean? or korean? or kosovar? or kosovan? or kyrgyz* or lao or laotian? or latvian? or lebanese or lesothan? or lesothonian? or mosotho or basotho or liberian? or libyan? or lithuanian? or macanese or macedonian? or malagasy or madagascan? or malawian? or malaysian? or maldivian? or malian? or maltese or marshallese? or mauritanian? or mauritian? or mexican? or micronesian? or moldovan? or mongolian? or mongol or montenegrin? or moroccan? or mozambican? or burmese or myanma or namibian? or nauruan? or nepali or nepalese or netherlands antillean? or nicaraguan? or nigerien? or nigerian? or northern mariana islander? or mariana? or omani? or pakistani? or palauan? or panamanian? or papua new guinean? or paraguayan? or peruvian? or philippine? or philipine? or phillipine? or phillippine? or filipino? or filipina? or polish or pole or poles or portuguese or puerto rican? or romanian? or russian? or soviet people or soviet population or rwandan? or rwandese or ruandan? or ruandese or samoan? or sao tomean? or santomean? or saudi arabian? or saudi? or senegalese or serbian? or montenegrin? or seychellois or seychelloise? or sierra leonean? or slovak? or slovene? or solomon islander? or somali? or south african? or south sudanese or sri lankan? or ceylonese or kittitian? or nevisian? or saint lucian? or vincentian? or sudanese or surinamese? or syrian? or tajik? or tajikistani? or tanzanian? or tanganyikan? or thai or timorese? or togolese or tongan? or trinidadian? or tobagonian? or tunisian? or turk? or turkish or turkmen? or tuvaluan? or ugandan? or ukrainian? or uruguayan? or uzbek? or vanuatu* or venezuelan? or vietnamese or yemeni? or yemenite? or yemenese or yugoslav? or yugoslavian? or zambian? or zimbabwean?).ti,ab,sh,kw. (120512)

8 6 or 7 (320416)
9 5 and 8 (23130)
10 (slum or slums or ghetto or ghettos or shanty$ or shanties or shack$ or favela$).ti,ab. (1156)
11 (informal$ adj3 settlement$).ti,ab. (206)
12 ((poverty or impoverish$) adj3 (area$ or settlement$)).ti,ab. (379)
13 ((precarious$ or irregular$) adj3 settlement$).ti,ab. (11)
14 (squatter$ adj3 (area$ or settlement$)).ti,ab. (70)
15 or/10-14 (1743)
16 (cost$ adj2 (illness$ or sickness$ or disease$)).ti,ab. (415)
17 (cost$ adj3 (care or health or healthcare or medical$)).ti,ab. (3305)
18 (expenditure$ adj3 (care or health or healthcare or medical$)).ti,ab. (2943)
19 ((direct or indirect) adj2 (cost$ or expenditure$)).ti,ab. (1415)
20 out of pocket.ti,ab. (868)
21 (OOP adj3 (cost$ or expense$ or expenditure$ or financ$ or pay$ or paid or spend$)).ti,ab. (71)
22 OOPE.ti,ab. (4)
23 ((personal or individual$ or patient$ or family$ or families or household$) adj3 (budget$ or cost$ or expense$ or expenditure$ or financ$ or pay$ or paid or spend$) adj3 (care or health or healthcare or medical$)).ti,ab. (599)
24 (catastroph$ adj10 (cost$ or expense$ or expenditure$ or financ$ or pay$ or paid or spend$)).ti,ab. (449)
25 ((medical$ or health) adj3 impoverish$).ti,ab. (23)
26 (burden$ adj3 (cost$ or expenditure$ or economic$ or financ$) adj3 (care or health or healthcare or medical$)).ti,ab. (106)
27 ((willing$ or unwilling$) adj3 pay$ adj3 (care or health or healthcare or medical$)).ti,ab. (127)
28 ((afford$ or unafford$) adj5 (cost$ or pay$)).ti,ab. (315)
29 ((able or ability or capacit$ or unable or inability) adj2 pay$).ti,ab. (717)
30 or/16-29 (9428)
31 9 and 30 (292)

32 15 and 30 (18)
33 31 or 32 (302)
34 limit 33 to yr="2010 -Current" (197)

Key:
$ = truncation
* = truncation
" " = exact phrase search
? = stands for zero or one characters within a word or at the end of a word
ti,ab = terms in either title or abstract fields
kw = terms in keywords field
sh = terms in the subject heading field
adj3 = terms within three words of each other (any order)
adj = terms next to each other in the same order

Science Citation Index
via Web of Science, Clarivate Analytics https://clarivate.com/
1900 – 19th June 2020
Searched on: 22nd June 2020
Records retrieved: 1538

# 35 1,538 #32 not #33
Indexes=SCI-EXPANDED Timespan=2010-2020
# 34 1,922 #32 not #33
# 33 1,028,173 CF=conference
# 32 1,929 #31 OR #30
# 31 99 #29 AND #14
# 30 1,888 #29 AND #8
# 29 102,229 #28 OR #27 OR #26 OR #25 OR #24 OR #23 OR #22 OR #21 OR #20 OR #19 OR #18 OR #17 OR #16 OR #15
# 28 1,622 TS=((able or ability or capacit* or unable or inability) NEAR/2 pay*)
# 27 3,128 TS=((afford* or unafford*) NEAR/5 (cost* or pay*))
# 26 379 TS=((willing* or unwilling*) NEAR/3 pay* NEAR/3 (care or health or healthcare or medical*))
# 25 4,603 TS=(burden* NEAR/3 (cost* or expenditure* or economic* or financ*) NEAR/3 (care or health or healthcare or medical*))
# 24 207 TS=((medical* or health) NEAR/3 impoverish*)
# 23 1,220 TS=(catastroph* NEAR/10 (cost* or expense* or expenditure* or financ* or pay* or paid or spend*))
# 22 11,648 TS=((personal or individual* or patient* or family* or families or household*) NEAR/3 (budget* or cost* or expense* or expenditure* or financ* or pay* or paid or spend*) NEAR/3 (care or health or healthcare or medical*))
# 21 46 TS=OOPE
# 20 368 TS=(OOP NEAR/3 (cost* or expense* or expenditure* or financ* or pay* or paid or spend*))
# 19 4,311 TS="out of pocket"
# 18 16,894 TS=((direct or indirect) NEAR/2 (cost* or expenditure*))
# 17 11,186 TS=(expenditure* NEAR/3 (care or health or healthcare or medical*))
# 16 68,145 TS=(cost* NEAR/3 (care or health or healthcare or medical*))
# 15 8,010 TS=(cost* NEAR/2 (illness* or sickness* or disease*))
# 14 7,749 #13 OR #12 OR #11 OR #10 OR #9

For peer review only

13 99 TS=(squatter* NEAR/3 (area* or settlement*))
12 37 TS=((precarious* or irregular*) NEAR/3 settlement*)
11 1,094 TS=((poverty or impoverish*) NEAR/3 (area* or settlement*))
10 791 TS=(informal NEAR/3 settlement*)
9 6,013 TS=(slum or slums or ghetto or ghettos or shanty* or shanties or shack* or favela*)
8 186,228 #7 AND #4
7 2,792,724 #6 OR #5
6 589,412 TS=(afghan or afghans or afghani or albanian? algerian? or american samoan? or angolan? or antiguan? or barbudan? or argentine? or argentinian? or argentinean? or armenian? or aruban? or azerbaijani? or bahraini? or bangladeshi? or bangalees or bajan? or belarusian? or byelorussian? or belizean? or beninese? or bhutanese or bolivian? or bosnian? or botswana or batswana or brazilian? or brasilian? or bulgarian? or burkinabe or burkinese or burundian? or cape verdean? or cabo verdean? or cambodian? or khmer or cameroonian? or central african? or chadian? or chilean? or chinese or colombian? or comorian? or congolese or costa rican? or ivorian? or croatian? or cuban? or cypriot? or czech? or djiboutian? or dominican? or ecuadorian? or egyptian? or salvadoran? or equatorial guinean? or equatoguinean? or eritrean? or estonian? or swazi? or swati? or ethiopian? or fijian or gabonese or gabonaise or gambian? or georgian? or ghanaian? or gibraltarian? or greek? or grenadian? or guamanian? or guatemalan? or guinean? or bissau guinean? or guyanese or haitian? or honduran? or hungarian? or indian? or indonesian? or iranian? or iraqian? or iraqi? or manx or jamaican? or jordanian? or kazakhstani? or kenyan? or kirabati or kirabatian? or north korean? or korean? or kosovar? or kosovan? or kyrgyz* or lao or laotian? or latvian? or lebanese or lesothan? or lesothonian? or mosotho or basotho or liberian? or libyan? or lithuanian? or macanese or macedonian? or malagasy or madagascan? or malawian? or malaysian? or maldivian? or malian? or maltese or marshallese? or mauritanian? or mauritian? or mexican? or micronesian? or moldovan? or mongolian? or mongol or montenegrin? or moroccan? or mozambican? or burmese or myanma or namibian? or nauruan? or nepali or nepalese or netherlands antillean? or nicaraguan? or nigerien? or nigerian? or northern mariana islander? or mariana? or omani? or pakistani? or palauan? or panamanian? or papua new guinean? or paraguayan? or peruvian? or philippine? or philipine? or phillipine? or phillippine? or filipino? or filipina? or polish or pole or poles or portuguese or puerto rican? or romanian? or russian? or soviet people or soviet population or rwandan? or rwandese or ruandan? or ruandese or samoan? or sao tomean? or santomean? or saudi arabian? or saudi? or senegalese or serbian? or montenegrin? or seychellois or seychelloise? or sierra leonean? or slovak? or slovene? or solomon islander? or somali? or south african? or south sudanese or sri lankan? or ceylonese or kittitian? or nevisian? or saint lucian? or vincentian? or sudanese or surinamese? or syrian? or tajik? or tajikistani? or tanzanian? or tanganyikan? or thai or timorese? or togolese or tongan? or trinidadian? or tobagonian? or tunisian? or turk? or turkish or turkmen? or tuvaluan? or ugandan? or ukrainian? or uruguayan? or uzbek? or vanuatu* or venezuelan? or vietnamese or yemeni? or yemenite? or yemenese or yugoslav? or yugoslavian? or zambian? or zimbabwean?)
5 2,363,830 TS=(afghanistan or albania or algeria or "american samoa" or angola or "antigua and barbuda" or antigua or barbuda or argentina or armenia or armenian or aruba or azerbaijan or bahrain or bangladesh or barbados or "republic of belarus" or belarus or byelarus or belorussia or byelorussian or belize or "british honduras" or benin or dahomey or bhutan or bolivia or "bosnia and herzegovina" or bosnia or herzegovina or botswana or bechuanaland or brazil or brasil or bulgaria or "burkina faso" or "burkina fasso" or "upper volta" or burundi or urundi or "cabo verde" or "cape verde" or cambodia or kampuchea or "khmer republic" or cameroon or cameron or cameroun or "central african republic" or

For peer review only

"ubangi shari" or chad or chile or china or colombia or comoros or "comoro islands" or "iles comores" or mayotte or "democratic republic of the congo" or "democratic republic congo" or congo or zaire or "costa rica" or "cote d'ivoire" or "cote d' ivoire" or "cote divoire" or "cote d ivoire" or "ivory coast" or croatia or cuba or cyprus or "czech republic" or czechoslovakia or djibouti or "french somaliland" or dominica or "dominican republic" or ecuador or egypt or "united arab republic" or "el salvador" or "equatorial guinea" or "spanish guinea" or eritrea or estonia or eswatini or swaziland or ethiopia or fiji or gabon or "gabonese republic" or gambia or "georgia (republic) " or georgian or ghana or "gold coast" or gibraltar or greece or grenada or guam or guatemala or guinea or "guinea bissau" or guyana or "british guiana" or haiti or hispaniola or honduras or hungary or india or indonesia or timor or iran or iraq or "isle of man" or jamaica or jordan or kazakhstan or kazakh or kenya or "democratic people's republic of korea" or "republic of korea" or "north korea" or "south korea" or korea or kosovo or kyrgyzstan or kirghizia or kirgizstan or "kyrgyz republic" or kirghiz or laos or "lao pdr" or "lao people's democratic republic" or latvia or lebanon or "lebanese republic" or lesotho or basutoland or liberia or libya or "libyan arab jamahiriya" or lithuania or macau or macao or "macedonia (republic) " or macedonia or madagascar or "malagasy republic" or malawi or nyasaland or malaysia or "malay federation" or "malaya federation" or maldives or "indian ocean islands" or "indian ocean" or mali or malta or micronesia or "federated states of micronesia" or kiribati or "marshall islands" or nauru or "northern mariana islands" or palau or tuvalu or mauritania or mauritius or mexico or moldova or moldovian or mongolia or montenegro or morocco or ifni or mozambique or "portuguese east africa" or myanmar or burma or namibia or nepal or "netherlands antilles" or nicaragua or niger or nigeria or oman or muscat or pakistan or panama or "papua new guinea" or "new guinea" or paraguay or peru or philippines or philipines or phillipines or phillippines or poland or "polish people's republic" or portugal or "portuguese republic" or "puerto rico" or romania or russia or "russian federation" or ussr or "soviet union" or "union of soviet socialist republics" or rwanda or ruanda or samoa or "pacific islands" or polynesia or "samoan islands" or "navigator island" or "navigator islands" or "sao tome and principe" or "saudi arabia" or senegal or serbia or seychelles or "sierra leon"e or slovakia or "slovak republic" or slovenia or melanesia or "solomon island" or "solomon islands" or "norfolk island" or "norfolk islands" or somalia or "south africa" or "south sudan" or "sri lanka" or ceylon or "saint kitts and nevis" or "st. kitts and nevis" or "saint lucia" or "st. lucia" or "saint vincent and the grenadines" or "saint vincent" or "st. vincent" or grenadines or sudan or suriname or surinam or "dutch guiana" or "netherlands guiana" or syria or "syrian arab republic" or tajikistan or tadjikistan or tadzhikistan or tadzhik or tanzania or tanganyika or thailand or siam or "timor leste" or "east timor" or togo or "togolese republic" or tonga or "trinidad and tobago" or trinidad or tobago or tunisia or turkey or "turkey (republic) " or turkmenistan or turkmen or uganda or ukraine or uruguay or uzbekistan or uzbek or vanuatu or "new hebrides" or venezuela or vietnam or "viet nam" or "middle east" or "west bank" or gaza or palestine or yemen or yugoslavia or zambia or zimbabwe or "northern rhodesia" or "global south" or "africa south of the sahara" or "sub-saharan africa" or "subsaharan africa" or "africa, central" or "central africa" or "africa, northern" or "north africa" or "northern africa" or magreb or maghrib or sahara or "africa, southern" or "southern africa" or "africa, eastern" or "east africa" or "eastern africa" or "africa, western" or "west africa" or "western africa" or "west indies" or "indian ocean islands" or caribbean or "central america" or "latin america" or "south and central america" or "south america" or "asia, central" or "central asia" or "asia, northern" or "north asia" or "northern asia" or "asia, southeastern" or "southeastern asia" or "south eastern asia" or "southeast asia" or "south east asia" or "asia, western" or "western asia" or "europe, eastern" or "east europe" or "eastern europe" or "developing country" or "developing countries" or "developing nation" or "developing nations" or "developing population" or "developing populations" or "developing world" or "less

For peer review only

developed country" or "less developed countries" or "less developed nation" or "less developed nations" or "less developed population" or "less developed populations" or "less developed world" or "lesser developed country" or "lesser developed countries" or "lesser developed nation" or "lesser developed nations" or "lesser developed population" or "lesser developed populations" or "lesser developed world" or "under developed country" or "under developed countries" or "under developed nation" or "under developed nations" or "under developed population" or "under developed populations" or "under developed world" or "underdeveloped country" or "underdeveloped countries" or "underdeveloped nation" or "underdeveloped nations" or "underdeveloped population" or "underdeveloped populations" or "underdeveloped world" or "middle income country" or "middle income countries" or "middle income nation" or "middle income nations" or "middle income population" or "middle income populations" or "low income country" or "low income countries" or "low income nation" or "low income nations" or "low income population" or "low income populations" or "lower income country" or "lower income countries" or "lower income nation" or "lower income nations" or "lower income population" or "lower income populations" or "underserved country" or "underserved countries" or "underserved nation" or "underserved nations" or "underserved population" or "underserved populations" or "underserved world" or "under served country" or "under served countries" or "under served nation" or "under served nations" or "under served population" or "under served populations" or "under served world" or "deprived country" or "deprived countries" or "deprived nation" or "deprived nations" or "deprived population" or "deprived populations" or "deprived world" or "poor country" or "poor countries" or "poor nation" or "poor nations" or "poor population" or "poor populations" or "poor world" or "poorer country" or "poorer countries" or "poorer nation" or "poorer nations" or "poorer population" or "poorer populations" or "poorer world" or "developing economy" or "developing economies" or "less developed economy" or "less developed economies" or "lesser developed economy" or "lesser developed economies" or "under developed economy" or "under developed economies" or "underdeveloped economy" or "underdeveloped economies" or "middle income economy" or "middle income economies" or "low income economy" or "low income economies" or "lower income economy" or "lower income economies" or "low gdp" or "low gnp" or "low gross domestic" or "low gross national" or "lower gdp" or "lower gnp" or "lower gross domestic" or "lower gross national" or lmic or lmics or "third world" or "lami country" or "lami countries" or "transitional country" or "transitional countries" or "emerging economies" or "emerging economy" or "emerging nation" or "emerging nations")

4 517,582 #3 OR #2 OR #1

3 7,570 TS=((dense* or density or high* or large* or heavy or heavily) NEAR/3 (populat* or populous) NEAR/3 (area* or settlement* or district* or neighbourhood* or communit*))

2 121,532 TS=(metropolis or megalopolis or municipal* or conurbation* or suburb* or town or towns or township* or borough* or barrio or barrios)

1 422,185 TS=(urban* or city or cities or megacity or megacities or metropolitan)

Key:
* = truncation
" " = exact phrase search
? = stands for zero or one character
TS = topic search - terms in title, abstract, keyword fields
NEAR/3 = terms within three words of each other (any order)

Social Science Citation Index
via Web of Science, Clarivate Analytics https://clarivate.com/

For peer review only

1956 – 19th June 2020
Searched on: 22nd June 2020
Records retrieved: 1226

# 35 1,226 #32 not #33
Indexes=SSCI Timespan=2010-2020
# 34 1,545 #32 not #33
# 33 58,510 CF=conference
# 32 1,557 #31 OR #30
# 31 87 #29 AND #14
# 30 1,527 #29 AND #8
# 29 50,953 #28 OR #27 OR #26 OR #25 OR #24 OR #23 OR #22 OR #21 OR #20 OR #19 OR #18 OR #17 OR #16 OR #15
# 28 1,469 TS=((able or ability or capacit* or unable or inability) NEAR/2 pay*)
# 27 1,102 TS=((afford* or unafford*) NEAR/5 (cost* or pay*))
# 26 364 TS=((willing* or unwilling*) NEAR/3 pay* NEAR/3 (care or health or healthcare or medical*))
# 25 1,709 TS=(burden* NEAR/3 (cost* or expenditure* or economic* or financ*) NEAR/3 (care or health or healthcare or medical*))
# 24 235 TS=((medical* or health) NEAR/3 impoverish*)
# 23 1,100 TS=(catastroph* NEAR/10 (cost* or expense* or expenditure* or financ* or pay* or paid or spend*))
# 22 6,230 TS=((personal or individual* or patient* or family* or families or household*) NEAR/3 (budget* or cost* or expense* or expenditure* or financ* or pay* or paid or spend*) NEAR/3 (care or health or healthcare or medical*))
# 21 41 TS=OOPE
# 20 358 TS=(OOP NEAR/3 (cost* or expense* or expenditure* or financ* or pay* or paid or spend*))
# 19 3,675 TS="out of pocket"
# 18 6,153 TS=((direct or indirect) NEAR/2 (cost* or expenditure*))
# 17 9,050 TS=(expenditure* NEAR/3 (care or health or healthcare or medical*))
# 16 32,024 TS=(cost* NEAR/3 (care or health or healthcare or medical*))
# 15 3,308 TS=(cost* NEAR/2 (illness* or sickness* or disease*))
# 14 8,916 #13 OR #12 OR #11 OR #10 OR #9
# 13 357 TS=(squatter* NEAR/3 (area* or settlement*))
# 12 44 TS=((precarious* or irregular*) NEAR/3 settlement*)
# 11 1,297 TS=((poverty or impoverish*) NEAR/3 (area* or settlement*))
# 10 1,304 TS=(informal NEAR/3 settlement*)
# 9 6,468 TS=(slum or slums or ghetto or ghettos or shanty* or shanties or shack* or favela*)
# 8 103,299 #7 AND #4
# 7 984,954 #6 OR #5
# 6 197,784 TS=(afghan or afghans or afghani or albanian? algerian? or american samoan? or angolan? or antiguan? or barbudan? or argentine? or argentinian? or argentinean? or armenian? or aruban? or azerbaijani? or bahraini? or bangladeshi? or bangalees or bajan? or belarusian? or byelorussian? or belizean? or beninese? or bhutanese or bolivian? or bosnian? or botswana or batswana or brazilian? or brasilian? or bulgarian? or burkinabe or burkinese or burundian? or cape verdean? or cabo verdean? or cambodian? or khmer or cameroonian? or central african? or chadian? or chilean? or chinese or colombian? or comorian? or congolese or costa rican? or ivorian? or croatian? or cuban? or cypriot? or czech? or djiboutian? or dominican? or ecuadorian? or egyptian? or salvadoran? or equatorial

guinean? or equatoguinean? or eritrean? or estonian? or swazi? or swati? or ethiopian? or fijian or gabonese or gabonaise or gambian? or georgian? or ghanaian? or gibraltarian? or greek? or grenadian? or guamanian? or guatemalan? or guinean? or bissau guinean? or guyanese or haitian? or honduran? or hungarian? or indian? or indonesian? or iranian? or iraqian? or iraqi? or manx or jamaican? or jordanian? or kazakhstani? or kenyan? or kirabati or kirabatian? or north korean? or korean? or kosovar? or kosovan? or kyrgyz* or lao or laotian? or latvian? or lebanese or lesothan? or lesothonian? or mosotho or basotho or liberian? or libyan? or lithuanian? or macanese or macedonian? or malagasy or madagascan? or malawian? or malaysian? or maldivian? or malian? or maltese or marshallese? or mauritanian? or mauritian? or mexican? or micronesian? or moldovan? or mongolian? or mongol or montenegrin? or moroccan? or mozambican? or burmese or myanma or namibian? or nauruan? or nepali or nepalese or netherlands antillean? or nicaraguan? or nigerien? or nigerian? or northern mariana islander? or mariana? or omani? or pakistani? or palauan? or panamanian? or papua new guinean? or paraguayan? or peruvian? or philippine? or philipine? or phillipine? or phillippine? or filipino? or filipina? or polish or pole or poles or portuguese or puerto rican? or romanian? or russian? or soviet people or soviet population or rwandan? or rwandese or ruandan? or ruandese or samoan? or sao tomean? or santomean? or saudi arabian? or saudi? or senegalese or serbian? or montenegrin? or seychellois or seychelloise? or sierra leonean? or slovak? or slovene? or solomon islander? or somali? or south african? or south sudanese or sri lankan? or ceylonese or kittitian? or nevisian? or saint lucian? or vincentian? or sudanese or surinamese? or syrian? or tajik? or tajikistani? or tanzanian? or tanganyikan? or thai or timorese? or togolese or tongan? or trinidadian? or tobagonian? or tunisian? or turk? or turkish or turkmen? or tuvaluan? or ugandan? or ukrainian? or uruguayan? or uzbek? or vanuatu* or venezuelan? or vietnamese or yemeni? or yemenite? or yemenese or yugoslav? or yugoslavian? or zambian? or zimbabwean?)

5 882,717 TS=(afghanistan or albania or algeria or "american samoa" or angola or "antigua and barbuda" or antigua or barbuda or argentina or armenia or armenian or aruba or azerbaijan or bahrain or bangladesh or barbados or "republic of belarus" or belarus or byelarus or belorussia or byelorussian or belize or "british honduras" or benin or dahomey or bhutan or bolivia or "bosnia and herzegovina" or bosnia or herzegovina or botswana or bechuanaland or brazil or brasil or bulgaria or "burkina faso" or "burkina fasso" or "upper volta" or burundi or urundi or "cabo verde" or "cape verde" or cambodia or kampuchea or "khmer republic" or cameroon or cameron or cameroun or "central african republic" or "ubangi shari" or chad or chile or china or colombia or comoros or "comoro islands" or "iles comores" or mayotte or "democratic republic of the congo" or "democratic republic congo" or congo or zaire or "costa rica" or "cote d'ivoire" or "cote d' ivoire" or "cote divoire" or "cote d ivoire" or "ivory coast" or croatia or cuba or cyprus or "czech republic" or czechoslovakia or djibouti or "french somaliland" or dominica or "dominican republic" or ecuador or egypt or "united arab republic" or "el salvador" or "equatorial guinea" or "spanish guinea" or eritrea or estonia or eswatini or swaziland or ethiopia or fiji or gabon or "gabonese republic" or gambia or "georgia (republic) " or georgian or ghana or "gold coast" or gibraltar or greece or grenada or guam or guatemala or guinea or "guinea bissau" or guyana or "british guiana" or haiti or hispaniola or honduras or hungary or india or indonesia or timor or iran or iraq or "isle of man" or jamaica or jordan or kazakhstan or kazakh or kenya or "democratic people's republic of korea" or "republic of korea" or "north korea" or "south korea" or korea or kosovo or kyrgyzstan or kirghizia or kirgizstan or "kyrgyz republic" or kirghiz or laos or "lao pdr" or "lao people's democratic republic" or latvia or lebanon or "lebanese republic" or lesotho or basutoland or liberia or libya or "libyan arab jamahiriya" or lithuania or macau or macao or "macedonia (republic) " or macedonia or madagascar or "malagasy republic" or malawi or nyasaland or malaysia or "malay federation" or "malaya

For peer review only

federation" or maldives or "indian ocean islands" or "indian ocean" or mali or malta or micronesia or "federated states of micronesia" or kiribati or "marshall islands" or nauru or "northern mariana islands" or palau or tuvalu or mauritania or mauritius or mexico or moldova or moldovian or mongolia or montenegro or morocco or ifni or mozambique or "portuguese east africa" or myanmar or burma or namibia or nepal or "netherlands antilles" or nicaragua or niger or nigeria or oman or muscat or pakistan or panama or "papua new guinea" or "new guinea" or paraguay or peru or philippines or philipines or phillipines or phillippines or poland or "polish people's republic" or portugal or "portuguese republic" or "puerto rico" or romania or russia or "russian federation" or ussr or "soviet union" or "union of soviet socialist republics" or rwanda or ruanda or samoa or "pacific islands" or polynesia or "samoan islands" or "navigator island" or "navigator islands" or "sao tome and principe" or "saudi arabia" or senegal or serbia or seychelles or "sierra leon"e or slovakia or "slovak republic" or slovenia or melanesia or "solomon island" or "solomon islands" or "norfolk island" or "norfolk islands" or somalia or "south africa" or "south sudan" or "sri lanka" or ceylon or "saint kitts and nevis" or "st. kitts and nevis" or "saint lucia" or "st. lucia" or "saint vincent and the grenadines" or "saint vincent" or "st. vincent" or grenadines or sudan or suriname or surinam or "dutch guiana" or "netherlands guiana" or syria or "syrian arab republic" or tajikistan or tadjikistan or tadzhikistan or tadzhik or tanzania or tanganyika or thailand or siam or "timor leste" or "east timor" or togo or "togolese republic" or tonga or "trinidad and tobago" or trinidad or tobago or tunisia or turkey or "turkey (republic) " or turkmenistan or turkmen or uganda or ukraine or uruguay or uzbekistan or uzbek or vanuatu or "new hebrides" or venezuela or vietnam or "viet nam" or "middle east" or "west bank" or gaza or palestine or yemen or yugoslavia or zambia or zimbabwe or "northern rhodesia" or "global south" or "africa south of the sahara" or "sub-saharan africa" or "subsaharan africa" or "africa, central" or "central africa" or "africa, northern" or "north africa" or "northern africa" or magreb or maghrib or sahara or "africa, southern" or "southern africa" or "africa, eastern" or "east africa" or "eastern africa" or "africa, western" or "west africa" or "western africa" or "west indies" or "indian ocean islands" or caribbean or "central america" or "latin america" or "south and central america" or "south america" or "asia, central" or "central asia" or "asia, northern" or "north asia" or "northern asia" or "asia, southeastern" or "southeastern asia" or "south eastern asia" or "southeast asia" or "south east asia" or "asia, western" or "western asia" or "europe, eastern" or "east europe" or "eastern europe" or "developing country" or "developing countries" or "developing nation" or "developing nations" or "developing population" or "developing populations" or "developing world" or "less developed country" or "less developed countries" or "less developed nation" or "less developed nations" or "less developed population" or "less developed populations" or "less developed world" or "lesser developed country" or "lesser developed countries" or "lesser developed nation" or "lesser developed nations" or "lesser developed population" or "lesser developed populations" or "lesser developed world" or "under developed country" or "under developed countries" or "under developed nation" or "under developed nations" or "under developed population" or "under developed populations" or "under developed world" or "underdeveloped country" or "underdeveloped countries" or "underdeveloped nation" or "underdeveloped nations" or "underdeveloped population" or "underdeveloped populations" or "underdeveloped world" or "middle income country" or "middle income countries" or "middle income nation" or "middle income nations" or "middle income population" or "middle income populations" or "low income country" or "low income countries" or "low income nation" or "low income nations" or "low income population" or "low income populations" or "lower income country" or "lower income countries" or "lower income nation" or "lower income nations" or "lower income population" or "lower income populations" or "underserved country" or "underserved countries" or "underserved nation" or "underserved nations" or "underserved population" or "underserved populations" or "underserved world" or "under

For peer review only

served country" or "under served countries" or "under served nation" or "under served nations" or "under served population" or " under served populations" or "under served world" or "deprived country" or "deprived countries" or "deprived nation" or "deprived nations" or "deprived population" or "deprived populations" or "deprived world" or "poor country" or "poor countries" or "poor nation" or "poor nations" or "poor population" or "poor populations" or "poor world" or "poorer country" or "poorer countries" or "poorer nation" or "poorer nations" or "poorer population" or "poorer populations" or "poorer world" or "developing economy" or "developing economies" or "less developed economy" or "less developed economies" or "lesser developed economy" or "lesser developed economies" or "under developed economy" or "under developed economies" or "underdeveloped economy" or "underdeveloped economies" or "middle income economy" or "middle income economies" or "low income economy" or "low income economies" or "lower income economy" or "lower income economies" or "low gdp" or "low gnp" or "low gross domestic" or "low gross national" or "lower gdp" or "lower gnp" or "lower gross domestic" or "lower gross national" or lmic or lmics or "third world" or "lami country" or "lami countries" or "transitional country" or "transitional countries" or "emerging economies" or "emerging economy" or "emerging nation" or "emerging nations")
4 354,340 #3 OR #2 OR #1
3 2,339 TS=((dense* or density or high* or large* or heavy or heavily) NEAR/3 (populat* or populous) NEAR/3 (area* or settlement* or district* or neighbourhood* or communit*))
2 70,206 TS=(metropolis or megalopolis or municipal* or conurbation* or suburb* or town or towns or township* or borough* or barrio or barrios)
1 309,493 TS=(urban* or city or cities or megacity or megacities or metropolitan)

Key:
* = truncation
" " = exact phrase search
? = stands for zero or one character
TS = topic search - terms in title, abstract, keyword fields
NEAR/3 = terms within three words of each other (any order)

Global Index Medicus
https://www.globalindexmedicus.net/
Searched on: 23rd June 2020
Records retrieved: 1274

6 separate searches were carried out and results deduplicated in EndNote.

1. (tw:(urban* OR city OR cities OR megacity OR megacities OR metropolitan OR metropolis OR megalopolis OR municipal* OR conurbation* OR suburb* OR town OR towns OR township* OR borough* OR barrio OR barrios OR "Urban Population" OR "Urban Health Services" OR "Hospitals, Municiple" OR "Urban Health" OR "Hospitals, Urban")) AND (ti:(cost or costs or expenditure* or expense* or spend* or pay* or paid or budget* or financ* or economic*)) AND (tw:(care OR healthcare OR health OR medical*))
2010-2020
607 hits

2. (tw:(urban* OR city OR cities OR megacity OR megacities OR metropolitan OR metropolis OR megalopolis OR municipal* OR conurbation* OR suburb* OR town OR towns OR township* OR borough* OR barrio OR barrios OR "Urban Population" OR "Urban Health Services" OR "Hospitals,

Municiple" OR "Urban Health" OR "Hospitals, Urban")) AND (tw:("Health Care Costs" OR "Health Expenditures" OR "Cost of Illness" OR "Financing Personal" OR "Catastrophic Expenditure" OR "Catastrophic Illness/EC" OR catastroph* OR "out of pocket" OR oop OR oope OR "willingness to pay"))
2010-2020
530 hits

3. (tw:(slum OR slums OR ghetto OR ghettos OR shanty* OR shanties OR shack* OR favela* OR squat* OR "informal settlement" OR "informal settlements" OR "Poverty Areas")) AND (tw:(cost or costs or expenditure* or expense* or spend* or pay* or paid or budget* or financ* or economic*)) AND (tw:(care OR health OR healthcare OR medical*))
2010-2020
189 hits

4. tw:(slum OR slums OR ghetto OR ghettos OR shanty* OR shanties OR shack* OR favela* OR squat* OR "informal settlement" OR "informal settlements" OR "Poverty Areas")) AND (tw:("Health Care Costs" OR "Health Expenditures" OR "Cost of Illness" OR "Financing Personal" OR "Catastrophic Expenditure" OR "Catastrophic Illness/EC" OR catastroph* OR "out of pocket" OR oop OR oope OR "willingness to pay"))
2010-2020
10 hits

5. (tw:(urban* OR city OR cities OR megacity OR megacities OR metropolitan OR metropolis OR megalopolis OR municipal* OR conurbation* OR suburb* OR town OR towns OR township* OR borough* OR barrio OR barrios OR "Urban Population" OR "Urban Health Services" OR "Hospitals, Municiple" OR "Urban Health" OR "Hospitals, Urban")) AND (tw:(impoverish*)) AND (tw:(health OR medical*))
2010-2020
188 hits

6. (tw:(slum OR slums OR ghetto OR ghettos OR shanty* OR shanties OR shack* OR favela* OR squat* OR "informal settlement" OR "informal settlements" OR "Poverty Areas")) AND (tw:(impoverish*)) AND (tw:(health OR medical*))
2010-2020
24 hits

Key:
tw = terms in the title, abstract or subject heading fields
* = truncation
" " = exact phrase

ProQuest Dissertations & Theses A&I
via ProQuest https://www.proquest.com/
Searched on: 23rd June 2020
Records retrieved: 283

10 separate searches were carried out and results deduplicated in EndNote. The following search line was added to each search to limit retrieval to low- middle-income countries:

(TI,AB,SU(afghanistan OR albania OR algeria OR "american samoa" OR angola OR "antigua and barbuda" OR antigua OR barbuda OR argentina OR armenia OR armenian OR aruba OR azerbaijan OR

For peer review only

bahrain OR bangladesh OR barbados OR "republic of belarus" OR belarus OR byelarus OR belorussia OR byelorussian OR belize OR "british honduras" OR benin OR dahomey OR bhutan OR bolivia OR "bosnia and herzegovina" OR bosnia OR herzegovina OR botswana OR bechuanaland OR brazil OR brasil OR bulgaria OR "burkina faso" OR "burkina fasso" OR "upper volta" OR burundi OR urundi OR "cabo verde" OR "cape verde" OR cambodia OR kampuchea OR "khmer republic" OR cameroon OR cameron OR cameroun OR "central african republic" OR "ubangi shari" OR chad OR chile OR china OR colombia OR comoros OR "comoro islands" OR "iles comores" OR mayotte OR "democratic republic of the congo" OR "democratic republic congo" OR congo OR zaire OR "costa rica" OR "cote d’ivoire" OR "cote d’ ivoire" OR "cote divoire" OR "cote d ivoire" OR "ivory coast" OR croatia OR cuba OR cyprus OR "czech republic" OR czechoslovakia OR djibouti OR "french somaliland" OR dominica OR "dominican republic" OR ecuador OR egypt OR "united arab republic" OR "el salvador" OR "equatorial guinea" OR "spanish guinea" OR eritrea OR estonia OR eswatini OR swaziland OR ethiopia OR fiji OR gabon OR "gabonese republic" OR gambia OR "georgia (republic) " OR georgian OR ghana OR "gold coast" OR gibraltar OR greece OR grenada OR guam OR guatemala OR guinea OR "guinea bissau" OR guyana OR "british guiana" OR haiti OR hispaniola OR honduras OR hungary OR india OR indonesia OR timor OR iran OR iraq OR "isle of man" OR jamaica OR jordan OR kazakhstan OR kazakh OR kenya OR "democratic people’s republic of korea" OR "republic of korea" OR "north korea" OR "south korea" OR korea OR kosovo OR kyrgyzstan OR kirghizia OR kirgizstan OR "kyrgyz republic" OR kirghiz OR laos OR "lao pdr" OR "lao people's democratic republic" OR latvia OR lebanon OR "lebanese republic" OR lesotho OR basutoland OR liberia OR libya OR "libyan arab jamahiriya" OR lithuania OR macau OR macao OR "macedonia (republic) " OR macedonia OR madagascar OR "malagasy republic" OR malawi OR nyasaland OR malaysia OR "malay federation" OR "malaya federation" OR maldives OR "indian ocean islands" OR "indian ocean" OR mali OR malta OR micronesia OR "federated states of micronesia" OR kiribati OR "marshall islands" OR nauru OR "northern mariana islands" OR palau OR tuvalu OR mauritania OR mauritius OR mexico OR moldova OR moldovian OR mongolia OR montenegro OR morocco OR ifni OR mozambique OR "portuguese east africa" OR myanmar OR burma OR namibia OR nepal OR "netherlands antilles" OR nicaragua OR niger OR nigeria OR oman OR muscat OR pakistan OR panama OR "papua new guinea" OR "new guinea" OR paraguay OR peru OR philippines OR philipines OR phillipines OR phillippines OR poland OR "polish people's republic" OR portugal OR "portuguese republic" OR "puerto rico" OR romania OR russia OR "russian federation" OR ussr OR "soviet union" OR "union of soviet socialist republics" OR rwanda OR ruanda OR samoa OR "pacific islands" OR polynesia OR "samoan islands" OR "navigator island" OR "navigator islands" OR "sao tome and principe" OR "saudi arabia" OR senegal OR serbia OR seychelles OR "sierra leon" e OR slovakia OR "slovak republic" OR slovenia OR melanesia OR "solomon island" OR "solomon islands" OR "norfolk island" OR "norfolk islands" OR somalia OR "south africa" OR "south sudan" OR "sri lanka" OR ceylon OR "saint kitts and nevis" OR "st. kitts and nevis" OR "saint lucia" OR "st. lucia" OR "saint vincent and the grenadines" OR "saint vincent" OR "st. vincent" OR grenadines OR sudan OR suriname OR surinam OR "dutch guiana" OR "netherlands guiana" OR syria OR "syrian arab republic" OR tajikistan OR tadjikistan OR tadzhikistan OR tadzhik OR tanzania OR tanganyika OR thailand OR siam OR "timor leste" OR "east timor" OR togo OR "togolese republic" OR tonga OR "trinidad and tobago" OR trinidad OR tobago OR tunisia OR turkey OR "turkey (republic) " OR turkmenistan OR turkmen OR uganda OR ukraine OR uruguay OR uzbekistan OR uzbek OR vanuatu OR "new hebrides" OR venezuela OR vietnam OR "viet nam" OR "middle east" OR "west bank" OR gaza OR palestine OR yemen OR yugoslavia OR zambia OR zimbabwe OR "northern rhodesia" OR "global south" OR "africa south of the sahara" OR "sub-saharan africa" OR "subsaharan africa" OR "africa, central" OR "central africa" OR "africa, northern" OR "north africa" OR "northern africa" OR magreb OR maghrib OR sahara OR "africa, southern" OR "southern africa" OR "africa, eastern" OR "east africa" OR "eastern africa" OR "africa, western" OR "west africa" OR "western africa" OR "west indies" OR "indian ocean islands" OR caribbean OR "central america" OR "latin america" OR "south and central america" OR "south america" OR "asia, central" OR "central asia" OR "asia, northern" OR "north asia" OR "northern asia" OR "asia, southeastern" OR "southeastern asia"

OR "south eastern asia" OR "southeast asia" OR "south east asia" OR "asia, western" OR "western asia" OR "europe, eastern" OR "east europe" OR "eastern europe" OR "developing country" OR "developing countries" OR "developing nation" OR "developing nations" OR "developing population" OR "developing populations" OR "developing world" OR "less developed country" OR "less developed countries" OR "less developed nation" OR "less developed nations" OR "less developed population" OR "less developed populations" OR "less developed world" OR "lesser developed country" OR "lesser developed countries" OR "lesser developed nation" OR "lesser developed nations" OR "lesser developed population" OR "lesser developed populations" OR "lesser developed world" OR "under developed country" OR "under developed countries" OR "under developed nation" OR "under developed nations" OR "under developed population" OR "under developed populations" OR "under developed world" OR "underdeveloped country" OR "underdeveloped countries" OR "underdeveloped nation" OR "underdeveloped nations" OR "underdeveloped population" OR "underdeveloped populations" OR "underdeveloped world" OR "middle income country" OR "middle income countries" OR "middle income nation" OR "middle income nations" OR "middle income population" OR "middle income populations" OR "low income country" OR "low income countries" OR "low income nation" OR "low income nations" OR "low income population" OR "low income populations" OR "lower income country" OR "lower income countries" OR "lower income nation" OR "lower income nations" OR "lower income population" OR "lower income populations" OR "underserved country" OR "underserved countries" OR "underserved nation" OR "underserved nations" OR "underserved population" OR "underserved populations" OR "underserved world" OR "under served country" OR "under served countries" OR "under served nation" OR "under served nations" OR "under served population" OR " under served populations" OR "under served world" OR "deprived country" OR "deprived countries" OR "deprived nation" OR "deprived nations" OR "deprived population" OR "deprived populations" OR "deprived world" OR "poor country" OR "poor countries" OR "poor nation" OR "poor nations" OR "poor population" OR "poor populations" OR "poor world" OR "poorer country" OR "poorer countries" OR "poorer nation" OR "poorer nations" OR "poorer population" OR "poorer populations" OR "poorer world" OR "developing economy" OR "developing economies" OR "less developed economy" OR "less developed economies" OR "lesser developed economy" OR "lesser developed economies" OR "under developed economy" OR "under developed economies" OR "underdeveloped economy" OR "underdeveloped economies" OR "middle income economy" OR "middle income economies" OR "low income economy" OR "low income economies" OR "lower income economy" OR "lower income economies" OR "low gdp" OR "low gnp" OR "low gross domestic" OR "low gross national" OR "lower gdp" OR "lower gnp" OR "lower gross domestic" OR "lower gross national" OR lmic OR lmics OR "third world" OR "lami country" OR "lami countries" OR "transitional country" OR "transitional countries" OR "emerging economies" OR "emerging economy" OR "emerging nation" OR "emerging nations")

1. LMIC strategy as above
AND (TI,AB,SU(urban* OR city OR cities OR megacity OR megacities OR metropolitan OR metropolis OR megalopolis OR municipal* OR conurbation* OR suburb* OR town OR towns OR township* OR borough* OR barrio OR barrios) OR TI,AB,SU((dense* OR density OR high* OR large* OR heavy OR heavily) NEAR/3 (populat OR populous) NEAR/3 (area* OR settlement* OR district* OR neighbourhood* OR communit*)))) AND (TI,AB,SU(cost* NEAR/2 (illness* OR sickness* OR disease*)) OR TI,AB,SU(cost* NEAR/3 (care OR health OR healthcare OR medical*)) OR TI,AB,SU(expenditure* NEAR/3 (care OR health OR healthcare OR medical*)))
Date limit: 2010-01-01 - 2020-06-23
164 hits

2. LMIC strategy as above

AND (TI,AB,SU(urban* OR city OR cities OR megacity OR megacities OR metropolitan OR metropolis OR megalopolis OR municipal* OR conurbation* OR suburb* OR town OR towns OR township* OR borough* OR barrio OR barrios) OR TI,AB,SU((dense* OR density OR high* OR large* OR heavy OR heavily) NEAR/3 (populat OR populous) NEAR/3 (area* OR settlement* OR district* OR neighbourhood* OR communit*))) AND (TI,AB,SU((direct OR indirect) NEAR/2 (cost* OR expenditure*)) OR TI,AB,SU("out of pocket") OR TI,AB,SU(OOP NEAR/3 (cost* OR expense* OR expenditure* OR financ* OR pay* OR paid OR spend*)) OR TI,AB,SU(OOPE))
Date limit: 2010-01-01 - 2020-06-23
52 hits

3. LMIC strategy as above
AND (TI,AB,SU(urban* OR city OR cities OR megacity OR megacities OR metropolitan OR metropolis OR megalopolis OR municipal* OR conurbation* OR suburb* OR town OR towns OR township* OR borough* OR barrio OR barrios) OR TI,AB,SU((dense* OR density OR high* OR large* OR heavy OR heavily) NEAR/3 (populat OR populous) NEAR/3 (area* OR settlement* OR district* OR neighbourhood* OR communit*))) AND TI,AB,SU((personal OR individual* OR patient* OR family* OR families OR household*) NEAR/3 (budget* OR cost* OR expense* OR expenditure* OR financ* OR pay* OR paid OR spend*) NEAR/3 (care OR health OR healthcare OR medical*))
Date limit: 2010-01-01 - 2020-06-23
27 hits

4. LMIC strategy as above
AND (TI,AB,SU(urban* OR city OR cities OR megacity OR megacities OR metropolitan OR metropolis OR megalopolis OR municipal* OR conurbation* OR suburb* OR town OR towns OR township* OR borough* OR barrio OR barrios) OR TI,AB,SU((dense* OR density OR high* OR large* OR heavy OR heavily) NEAR/3 (populat OR populous) NEAR/3 (area* OR settlement* OR district* OR neighbourhood* OR communit*))) AND TI,AB,SU(catastroph* NEAR/10 (cost* OR expense* OR expenditure* OR financ* OR pay* OR paid OR spend*))
Date limit: 2010-01-01 - 2020-06-23
8 hits

5. LMIC strategy as above
AND (TI,AB,SU(urban* OR city OR cities OR megacity OR megacities OR metropolitan OR metropolis OR megalopolis OR municipal* OR conurbation* OR suburb* OR town OR towns OR township* OR borough* OR barrio OR barrios) OR TI,AB,SU((dense* OR density OR high* OR large* OR heavy OR heavily) NEAR/3 (populat OR populous) NEAR/3 (area* OR settlement* OR district* OR neighbourhood* OR communit*))) AND TI,AB,SU((willing* OR unwilling*) NEAR/3 (pay*) NEAR/3 (care OR health OR healthcare OR medical*))
Date limit: 2010-01-01 - 2020-06-23
1 hit

6. LMIC strategy as above
AND (TI,AB,SU(urban* OR city OR cities OR megacity OR megacities OR metropolitan OR metropolis OR megalopolis OR municipal* OR conurbation* OR suburb* OR town OR towns OR township* OR borough* OR barrio OR barrios) OR TI,AB,SU((dense* OR density OR high* OR large* OR heavy OR heavily) NEAR/3 (populat OR populous) NEAR/3 (area* OR settlement* OR district* OR neighbourhood* OR communit*))) AND (TI,AB,SU((medical* OR health) NEAR/3 impoverish*) OR TI,AB,SU(burden* NEAR/3 (cost* OR expenditure* OR economic* OR financ*) NEAR/3 (care OR health OR healthcare OR medical*)) OR TI,AB,SU((afford* OR unafford*) NEAR/5 (cost* OR pay*)) OR TI,AB,SU((able OR ability OR capacit* OR unable OR inability) NEAR/2 pay*))
Date limit: 2010-01-01 - 2020-06-23

For peer review only

81 hits

7. LMIC strategy as above
AND (TI,AB,SU(slum OR slums OR ghetto OR ghettos OR shanty* OR shanties OR shack* OR favela*) OR TI,AB,SU((informal* OR precarious* OR irregular*) NEAR/3 settlement*) OR TI,AB,SU((poverty OR impoverish* OR squatter*) NEAR/3 (area* OR settlement*))) AND ((TI,AB,SU(cost* NEAR/2 (illness* OR sickness* OR disease*)) OR TI,AB,SU(cost* NEAR/3 (care OR health OR healthcare OR medical*)) OR TI,AB,SU(expenditure* NEAR/3 (care OR health OR healthcare OR medical*))) OR (TI,AB,SU((direct OR indirect) NEAR/2 (cost* OR expenditure*)) OR TI,AB,SU("out of pocket") OR TI,AB,SU(OOP NEAR/3 (cost* OR expense* OR expenditure* OR financ* OR pay* OR paid OR spend*)) OR TI,AB,SU(OOPE)))
Date limit: 2010-01-01 - 2020-06-23
15 hits

8. LMIC strategy as above
AND (TI,AB,SU(slum OR slums OR ghetto OR ghettos OR shanty* OR shanties OR shack* OR favela*) OR TI,AB,SU((informal* OR precarious* OR irregular*) NEAR/3 settlement*) OR TI,AB,SU((poverty OR impoverish* OR squatter*) NEAR/3 (area* OR settlement*))) AND TI,AB,SU((personal OR individual* OR patient* OR family* OR families OR household*) NEAR/3 (budget* OR cost* OR expense* OR expenditure* OR financ* OR pay* OR paid OR spend*) NEAR/3 (care OR health OR healthcare OR medical*))
Date limit: 2010-01-01 - 2020-06-23
3 hits

9. LMIC strategy as above
AND TI,AB,SU(slum OR slums OR ghetto OR ghettos OR shanty* OR shanties OR shack* OR favela*) OR TI,AB,SU((informal* OR precarious* OR irregular*) NEAR/3 settlement*) OR TI,AB,SU((poverty OR impoverish* OR squatter*) NEAR/3 (area* OR settlement*))) AND TI,AB,SU(catastroph* NEAR/10 (cost* OR expense* OR expenditure* OR financ* OR pay* OR paid OR spend*))
Date limit: 2010-01-01 - 2020-06-23
2 hits

10. LMIC strategy as above
AND (TI,AB,SU(slum OR slums OR ghetto OR ghettos OR shanty* OR shanties OR shack* OR favela*) OR TI,AB,SU((informal* OR precarious* OR irregular*) NEAR/3 settlement*) OR TI,AB,SU((poverty OR impoverish* OR squatter*) NEAR/3 (area* OR settlement*))) AND (TI,AB,SU((willing* OR unwilling*) NEAR/3 (pay*) NEAR/3 (care OR health OR healthcare OR medical*)) OR (TI,AB,SU((medical* OR health) NEAR/3 impoverish*) OR TI,AB,SU(burden* NEAR/3 (cost* OR expenditure* OR economic* OR financ*) NEAR/3 (care OR health OR healthcare OR medical*)) OR TI,AB,SU((afford* OR unafford*) NEAR/5 (cost* OR pay*)) OR TI,AB,SU((able OR ability OR capacit* OR unable OR inability) NEAR/2 pay*)))
Date limit: 2010-01-01 - 2020-06-23
9 hits

Key:
TI,AB,SU = terms in title, abstract, or subject heading fields
* = truncation
" " = exact phrase search
NEAR/3 = terms within three words of each other (any order)

For peer review only

Econpapers
https://econpapers.repec.org/

OpenGrey
http://www.opengrey.eu/

World Bank

OECD

References

1. Effective Practice and Organisation of Care (EPOC) Cochrane group. LMIC filters. The Cochrane Collaboration. 2020 [accessed 14th May 2020]. Available from: https://epoc.cochrane.org/lmic-filters

For peer review only

Reporting checklist for protocol of a systematic review.

Based on the PRISMA-P guidelines.

Instructions to authors

Complete this checklist by entering the page numbers from your manuscript where readers will find each of the items listed below.

Your article may not currently address all the items on the checklist. Please modify your text to include the missing information. If you are certain that an item does not apply, please write "n/a" and provide a short explanation.

Upload your completed checklist as an extra file when you submit to a journal.

In your methods section, say that you used the PRISMA-Preporting guidelines, and cite them as:

Moher D, Shamseer L, Clarke M, Ghersi D, Liberati A, Petticrew M, Shekelle P, Stewart LA. Preferred Reporting Items for Systematic Review and Meta-Analysis Protocols (PRISMA-P) 2015 statement. Syst Rev. 2015;4(1):1.

		Reporting Item	Page Number
**Title**			
Identification	#1a	Identify the report as a protocol of a systematic review	1
Update	#1b	If the protocol is for an update of a previous systematic review, identify as such	N/A

For peer review only

**Registration**			
	#2	If registered, provide the name of the registry (such as PROSPERO) and registration number	5
**Authors**			
Contact	#3a	Provide name, institutional affiliation, e-mail address of all protocol authors; provide physical mailing address of corresponding author	1
Contribution	#3b	Describe contributions of protocol authors and identify the guarantor of the review	9
**Amendments**			
	#4	If the protocol represents an amendment of a previously completed or published protocol, identify as such and list changes; otherwise, state plan for documenting important protocol amendments	N/A
**Support**			
Sources	#5a	Indicate sources of financial or other support for the review	9
Sponsor	#5b	Provide name for the review funder and / or sponsor	9
Role of sponsor or funder	#5c	Describe roles of funder(s), sponsor(s), and / or institution(s), if any, in developing the protocol	9
**Introduction**			

For peer review only

Rationale	#6	Describe the rationale for the review in the context of what is already known	4
Objectives	#7	Provide an explicit statement of the question(s) the review will address with reference to participants, interventions, comparators, and outcomes (PICO)	5
**Methods**			
Eligibility criteria	#8	Specify the study characteristics (such as PICO, study design, setting, time frame) and report characteristics (such as years considered, language, publication status) to be used as criteria for eligibility for the review	7
Information sources	#9	Describe all intended information sources (such as electronic databases, contact with study authors, trial registers or other grey literature sources) with planned dates of coverage	6
Search strategy	#10	Present draft of search strategy to be used for at least one electronic database, including planned limits, such that it could be repeated	6
Study records - data management	#11a	Describe the mechanism(s) that will be used to manage records and data throughout the review	6
Study records - selection process	#11b	State the process that will be used for selecting studies (such as two independent reviewers) through each phase of the review (that is, screening, eligibility and inclusion in meta-analysis)	7

For peer review only

Study records - data collection process	#11c	Describe planned method of extracting data from reports (such as piloting forms, done independently, in duplicate), any processes for obtaining and confirming data from investigators	7
Data items	#12	List and define all variables for which data will be sought (such as PICO items, funding sources), any pre-planned data assumptions and simplifications	6, 8
Outcomes and prioritization	#13	List and define all outcomes for which data will be sought, including prioritization of main and additional outcomes, with rationale	8
Risk of bias in individual studies	#14	Describe anticipated methods for assessing risk of bias of individual studies, including whether this will be done at the outcome or study level, or both; state how this information will be used in data synthesis	7
Data synthesis	#15a	Describe criteria under which study data will be quantitatively synthesised	8
Data synthesis	#15b	If data are appropriate for quantitative synthesis, describe planned summary measures, methods of handling data and methods of combining data from studies, including any planned exploration of consistency (such as I2, Kendall's τ)	8
Data synthesis	#15c	Describe any proposed additional analyses (such as sensitivity or subgroup analyses, meta-regression)	8

For peer review only

Data synthesis	#15d	If quantitative synthesis is not appropriate, describe the type of summary planned	N/A
Meta-bias(es)	#16	Specify any planned assessment of meta-bias(es) (such as publication bias across studies, selective reporting within studies)	N/A
Confidence in cumulative evidence	#17	Describe how the strength of the body of evidence will be assessed (such as GRADE)	N/A

The PRISMA-P checklist is distributed under the terms of the Creative Commons Attribution License CC-BY 4.0. This checklist was completed on 30. September 2020 using https://www.goodreports.org/, a tool made by the EQUATOR Network in collaboration with Penelope.ai

For peer review only

BMJ Open

The economics of health-care access in low and middle-income countries: A protocol for a scoping review of the economic impacts of seeking health care on slum-dwellers compared with other city residents.

Journal:	*BMJ Open*
Manuscript ID	bmjopen-2020-045441.R1
Article Type:	Protocol
Date Submitted by the Author:	17-Jun-2021
Complete List of Authors:	Teixeira de Siqueira-Filha, Noemia; University of York<br>Li, Jinshuo; University of York, Department of Health Sciences<br>Kibuchi, Eliud; University of Glasgow<br>Quayyum , Zahidul ; BRAC University James P Grant School of Public Health,<br>Phillips-Howard, Penelope; Liverpool School of Tropical Medicine, Department of Clinical Sciences<br>Awal, Abdul; BRAC<br>Mithu, Md. Imran Hossain; BRAC<br>Manzoor, Farzana; BRAC<br>Karuga, Robinson; LVCT<br>Saidu, Samuel; COMAHS<br>Smith, John; COMAHS<br>Sai, Varun; The George Institute for Global Health India<br>Garimella, Sureka; The George Institute for Global Health India<br>Chumo, Ivy; African Population and Health Research Center<br>Mberu, Blessing; African Population and Health Research Center<br>Tolhurst, Rachel; Liverpool School of Tropical Medicine, Clinical Sciences and International Public Health<br>Mazumdar, Sumit; Centre for Health Economics<br>Rao, Vinodkumar ; The Society for Promotion of Area Resource Center<br>Farnaz, Nadia; BRAC University James P Grant School of Public Health, School of Public Health; BRAC University James P Grant School of Public Health, School of Public Health<br>Alam, Wafa; BRAC University,<br>Elsey, Helen ; University of York,
**Primary Subject Heading**:	Health economics
Secondary Subject Heading:	Global health, Health economics, Health services research
Keywords:	HEALTH ECONOMICS, HEALTH SERVICES ADMINISTRATION & MANAGEMENT, Health policy < HEALTH SERVICES ADMINISTRATION & MANAGEMENT, Protocols & guidelines < HEALTH SERVICES ADMINISTRATION & MANAGEMENT

I, the Submitting Author has the right to grant and does grant on behalf of all authors of the Work (as defined in the below author licence), an exclusive licence and/or a non-exclusive licence for contributions from authors who are: i) UK Crown employees; ii) where BMJ has agreed a CC-BY licence shall apply, and/or iii) in accordance with the terms applicable for US Federal Government officers or employees acting as part of their official duties; on a worldwide, perpetual, irrevocable, royalty-free basis to BMJ Publishing Group Ltd ("BMJ") its licensees and where the relevant Journal is co-owned by BMJ to the co-owners of the Journal, to publish the Work in this journal and any other BMJ products and to exploit all rights, as set out in our licence.

The Submitting Author accepts and understands that any supply made under these terms is made by BMJ to the Submitting Author unless you are acting as an employee on behalf of your employer or a postgraduate student of an affiliated institution which is paying any applicable article publishing charge ("APC") for Open Access articles. Where the Submitting Author wishes to make the Work available on an Open Access basis (and intends to pay the relevant APC), the terms of reuse of such Open Access shall be governed by a Creative Commons licence – details of these licences and which Creative Commons licence will apply to this Work are set out in our licence referred to above.

Other than as permitted in any relevant BMJ Author's Self Archiving Policies, I confirm this Work has not been accepted for publication elsewhere, is not being considered for publication elsewhere and does not duplicate material already published. I confirm all authors consent to publication of this Work and authorise the granting of this licence.

For peer review only

Title: The economics of health-care access in low and middle-income countries: A protocol for a scoping review of the economic impacts of seeking health care on slum-dwellers compared with other city residents.

Collaborating authors: [1]Noemia Teixeira de Siqueira-Filha, [1]Jinshuo Li, [2]Eliud Kibuchi, [3]Zahidul Quayyum, [4]Penelope A Phillips-Howard, [3]Abdul Awal, [3]Md Imran Hossain Mithu, [3]Farzana Manzoor, [5]Robinson Karuga, [6]Samuel Saidu, [6]John Smith, [7]Varun Sai, [7]Sureka Garimella, [8] Ivy Chumo, [8] Blessing Mberu, [4]Rachel Tolhurst, [1]Sumit Mazumdar,[9]Vinodkumar Rao, [3]Nadia Farnaz, [3]Wafa Alam, [1]Helen Elsey

Noemia.teixeira.siqueira@gmail.com, jinshuo.li@york.ac.uk, Eliud.Kibuchi@glasgow.ac.uk, zahidul.quayyum@bracu.ac.bd, Penelope.Phillips-Howard@lstmed.ac.uk, abdul.awal@bracu.ac.bd, imran.mithu@bracu.ac.bd, farzana.manzoor@bracu.ac.bd, Rkaruga@lvcthealth.org, samuelsaidu0@gmail.com, smithhawa95@gmail.com, varun.sbbkey@gmail.com, sgarimella@georgeinstitute.org.in, ichumo@aphrc.org, bmberu@aphrc.org, Rachel.Tolhurst@lstmed.ac.uk, sumit.mazumdar@york.ac.uk, vinodkumar.rao@gmail.com, nadia.farnaz@bracu.ac.bd, wafa.alam@bracu.ac.bd, helen.elsey@york.ac.uk

[1] University of York, York, UK
[2] University of Glasgow, Glasgow, UK
[3] James P Grant School of Public Health, Brac University, Dhaka, Bangladesh
[4] Liverpool School of Tropical Medicine, Liverpool, UK
[5] LVCT Health, Nairobi, Kenya
[6] COMAHS: University of Sierra Leone, Freetown, Sierra Leone,
[7] The George Institute for Global Health, New Delhi, India
[8] African Population and Health Research Center, Nairobi, Kenya
[9] The Society for Promotion of Area Resource Centres, Mumbai, India

Corresponding author: Noemia Teixeira de Siqueira Filha, Noemia.teixeira.siqueira@gmail.com, Department of Health Sciences, Faculty of Science, University of York, Seebohm Rowntree Building (Area 2), University of York, Heslington, York-UK, YO10 5DD.

Key words: scoping review, informal settlements, costs, catastrophic health expenditures, low-middle-income countries

Word count: 2,684

For peer review only

For peer review only - http://bmjopen.bmj.com/site/about/guidelines.xhtml

Abstract

Introduction: People living in slums face several challenges to access healthcare. Scarce and low-quality public health facilities are common problems in these communities. Costs and prevalence of catastrophic health expenditures (CHE) have also been reported as high in studies conducted in slums in developing countries and those suffering from chronic conditions and the poorest households seem to be more vulnerable to financial hardship. The Covid-19 pandemic may be aggravating the economic impact on the extremely vulnerable population living in slums due to the long-term consequences of the disease. The objective of this review is to report the economic impact of seeking health care on slum-dwellers in terms of costs and CHE. We will compare the economic impact on slum-dwellers with other city residents.

Methods and analysis: This scoping review adopts the framework suggested by Arksey and O'Malley. The review is part of the ARISE research consortium, which aims to enhance accountability to improve the health and wellbeing of marginalized populations living in slums in India, Bangladesh, Sierra Leone, and Kenya. Costs of accessing health care will be updated to 2020 prices using the inflation rates reported by the International Monetary Fund. Costs will be presented in International Dollars by using purchase power parity. The prevalence of CHE will also be reported.

Ethics and dissemination: Ethical approval is not required for scoping reviews. We will disseminate our results alongside the events organized by the ARISE consortium and international conferences. The final manuscript will be submitted to an open-access international journal.

Registration number at the Research Registry: reviewregistry947

For peer review only - http://bmjopen.bmj.com/site/about/guidelines.xhtml

Strengths and limitations of this study

- This is a comprehensive scoping review that includes studies addressing costs of illness and cost of accessing health care to identify inequities within the urban context.
- There is no language restriction in this review, per reviewed studies, grey literature and reports from12 databases including leading organizations on urban health research will be searched.
- We will assess the quality of studies by using the Consolidated Health Economic Evaluation Reporting Standards and the Tool to Estimate Patient's Costs.
- Slums communities in rural areas will not be included in this review.

For peer review only

For peer review only - http://bmjopen.bmj.com/site/about/guidelines.xhtml

1. Background

The United Nations estimates that an increase in population and migration will add a further 2.5 billion people to the urban population by 2050. Ninety per cent of this growth will occur in Asian and African countries.[1] In 2014, 30% of the total urban population in developing countries were living in slums.[2] The absolute number of 881,080,000 people is close to the entire population of the two most populous countries in the world, India (1.4 billion inhabitants) and China (1.3 billion inhabitants).[3] While urbanization is one possible driver of economic growth,[4] it also increases inequities within cities and those living in slum households and neighbourhoods suffer grossly inadequate access to health care, clean water, sanitation, safe housing and basic amenities.[5]

Although the traditional assumption describes an urban advantage in accessing health-care,[6] many barriers to access remain, and physical proximity to health providers does not always translate to better health, particularly for the urban poor.[7] While analysis of demographic and health surveys (DHSs) across 73 low and middle-income countries (LMICs) showed that children in urban slums have better health than their rural counterparts,[8] this masks intra urban inequities. For example, under 5 mortality in Kenya and Bangladesh was higher in urban slums than in rural areas (Kenya: 79.8 vs 56 per 1000 live births; Bangladesh: 57 vs 49 per 1000 live births).[7,9] These conflicting data may in part be due to limitations in traditional sampling methods used by DHS and other national surveys where urban poor are frequently under-represented.[10,11]

Urbanisation has been seen as a determinant of health and wellbeing, resulting in a double burden of communicable and non-communicable diseases (NCDs).[12] A spectrum of research has shown how slums constitute unhealthy places, posing critical health challenges for urban residents. Children living in slums are particularly vulnerable to malnutrition, recurrent diarrhoea, stunted growth, and long-term cognitive development challenges.[13,14] Prevalence of diabetes and hypertension,[6,15] and poor mental health have increased dramatically in slums.[7,8] Injuries, both unintentional and as a result of violence,[16] nutrition transition,[17,18] obesity,[19] tobacco[20,21] and alcohol abuse[22,23] are also disproportionately high in slum communities. All of these studies highlight how these poor health conditions are driven by the stresses, work patterns and changing social and gender norms within cities. There is also increasing evidence on the role of socioeconomic factors contributing to common forms of mental illness in slum-dwellers, further exacerbating the burden of disease and inequities related to mental health in these communities.[24]

Provider pluralism is common in many cities in LMICs and some governments have encouraged the adoption of pluralistic models for healthcare delivery in slums, establishing public-private partnerships between Non-Governmental Organizations (NGOs), government and private providers. The Urban Primary Health Care Programme project, implemented by the government of Bangladesh, has positively influenced health outcomes for the urban poor in the country.[25] NGOs have frequently led the way in providing health care services for slum-dwellers, leading to some reduction in intra-urban disparities, particularly in terms of reproductive health.[26]

While this pluralism of providers may improve physical access of poor urban communities to health-care, several studies have described the barriers faced by slum-dweller on the pathway to health services. Contentious relationships between city authorities and slums-dwellers,[27] and scant public health services whose the access needs to be negotiated through a 'middlemen'[28] have been reported in several slums. High out of pocket expenditure of urban residents, limited coverage of health insurance and access to subsidised government schemes, particularly among migrants who frequently lack the required registration documents[29–31] are also common. In addition, important differences in costs of accessing health care for chronic conditions were found among and within

For peer review only - http://bmjopen.bmj.com/site/about/guidelines.xhtml

slums in Bangladesh. Slum-dwellers in Tongi community incurred doble the costs of those residents of Sylhet (US$ 323 vs USD 169, 2014 prices). Whlist the wealthiest households (4th and 5th quintiles) were more likely to incur higher costs compared to the poorest households (1st and 2nd quintiles) at the same slum.[31] The prevalence of catastrophic health expenditure (CHE) has has also been reported as high. The degree of CHE varies according to the threshold and methods adopted to calculate this indicator.[32] The economic and social impacts of seeking health care for slum-dwellers may be further exacerbated by the Covid-19 outbreak due to the severity of the symptoms and its long-term consequences. Several reports have shown high incidence and mortality due to Covid-19 in slums areas of Chembur, Matunga and Dahisar in Mumbai, India.[33,34]

By understanding the structure of expenditure of urban poor residents on health care, we will be able to inform decisions on how best to remodel urban health systems and improve equitable access. The objective of this scoping review is therefore to map the evidence available on the economic impact of seeking health care on slum-dwellers and the urban poor compared with other city residents. We will quantify the economic impact in terms of CHE, direct and indirect costs incurred by these communities during the search for health care and treatment and identify any inequities with better-off urban residents.

Methods

This scoping review will be developed as part of the ARISE research consortium, funded by the Global Challenges Research Fund (GCRF). The consortium aims to enhance accountability and improve the equitable health and wellbeing of marginalised populations living in slums in LMICs. The review is part of the ARISE work package *Metrics, Epidemiology, and Economics,* which aims to collect metrics to reflect lived realities, inequities, and priorities for transformative social changes in slums by using epidemiological and health economics tools.[35]

This review protocol follows the framework suggested by Arksey and O'Malley, 2005[36] and Levac et al, 2010[37] and It was registered at the Research Registry (https://www.researchregistry.com/), ID: reviewregistry947. The review process and searches began in June 2020 and we aim to publish our findings by June 2022.

Identifying the research question

We formulated our research question based on the ARISE principle to generate evidence to promote accountability and responsiveness of slum-dwellers (ARISE, 2019). The PCC (population, concept, and context) framework was used to formulate the research question.[38] We will explore the concept of the economic impact of seeking health care on slum communities compared with other city-residents in the context of urban areas in LMICs. Box 1 shows the glossary of operational definitions adopted in this review.

For peer review only - http://bmjopen.bmj.com/site/about/guidelines.xhtml

For peer review only

Box 1. Glossary of operational definitions.

Low and middle income countries: Gross National Income (GNI) per capita of $1,035 or less for low income economies; GNI per capita between $1,036 and $4,045 for lower middle-income economies; and GNI per capita between $4,046 and $12,535 for upper middle-income economies.[3]
Urban informal settlements, slums: households living in urban areas who lack at least one of the following: security of housing tenure, easy access to water or sanitation, sufficient living space, durable housing.[39]
Economic impact: in this review, the economic impact is defined as the financial impact in terms of prevalence of catastrophic health expenditure, direct, indirect and total costs.
Catastrophic health expenditures (CHE): proportion of the household annual income committed with health care that results in financial hardship for the family. The World Health Organization (WHO) indicates a threshold of 40% of a household's non-subsistence income.[40] However, other thresholds have been applied in the calculation of CHE.
Direct costs: all costs due to resource use that are completely attributable to the use of a health care intervention or illness.[41]
Direct medical costs: out of pocket expenses incurred with medication, tests and health care facilities fees [41].
Direct non-medical costs: out of pocket expenses incurred with transportation, food, accommodation and care-givers.[41]
Indirect costs: income and time lost during the search for health care, while waiting for appointments and during hospitalization.[41]

Search strategy and identification of relevant studies

The search strategy aimed to identify studies examining the costs of healthcare for slum dwellers or city residents in LMICs. A search strategy was developed by an information specialist using Ovid MEDLINE and based on the review eligibility criteria indicated in Table 1.

Search terms were gathered by applying key concepts: slum dwellers, slums, informal settlements, and urban areas (population), healthcare costs (concept), and LMICs (context). Both text word and subject heading searches for each concept were included in the strategy. Retrieval was limited to publications within the last 10 years (2010-2020). No language restrictions were applied. The final search strategy for MEDLINE was agreed by NTSF and HE and then translated as appropriate for the other databases and resources. The complete search strategy is presented in the supplementary material.

Databases containing literature from the fields of health, economics and social science was searched in MEDLINE (Ovid), Embase (Ovid), EconLit (Ovid), Science Citation Index (Web of Science), Social Science Citation Index (Web of Science) and Global Index Medicus. Also, the following resources containing grey literature such as theses and dissertations, and reports from leading organizations on urban health research were searched: Proquest Dissertations and Theses (A&I), Econpapers, OpenGrey, the World Bank, the Organisation for Economic Co-operation and Development (OECD) and The UN-Habitat websites. EndNote was used for reference management and duplicate removal.

For peer review only

For peer review only - http://bmjopen.bmj.com/site/about/guidelines.xhtml

Table 1. Inclusion and exclusion criteria of the scoping review.

Appraisal	Inclusion Criteria	Exclusion Criteria
Country	Low and middle-income countries	High income countries
Article type	Peer reviewed published articles, theses and reports from the World Bank, Organisation for Economic Co-operation and Development and UN-Habitat	Case reports, protocols, news article, editorial, conference abstracts, comments
Study design	Economic evaluations, cost and cost-effectiveness studies	Willingness to pay, health finance and economic evaluations addressing the provider perspective, systematic and scoping reviews
Focus of study	Access to health care: NGOs, private and public sector, outpatient and inpatient care. Studies that disaggregate by slum/non-slum or wealth quintile/poor/non-poor; studies that are slum specific	Studies that do not disaggregate between rural and urban or urban wealth categories/slum and city level; studies focused on rural areas

Selection of papers and data extraction

Two reviewers will independently screen the studies by title and abstract generating two lists of retrieved studies to be compared following the pathway indicated on Figure 1. The number of records identified, duplicates removed, titles and abstracts screened, studies retrieved and included in the review will be recorded in a PRISMA flowchart.[42] Rayyan software (http://rayyan.qcri.org)[43] will be used to manage the screening of all identified studies.

The data extraction will also be conducted by two reviewers, who will extract the data using a pre-designed extraction form in the format of Excel spreadsheet. Discordances in the inclusion of studies and data extraction will be discussed with a third researcher and agreement reached by consensus. We will extract the following information: location (country/city), characteristics of the health sector, socio-economic profile of the study population, study design, sampling method, and sample size, measure of spending/payment , type of costs included, cost analysis, and method applied to calculate catastrophic costs, types of health-care services used, types of health conditions, quality of health care, study outcomes (costs and catastrophic health expenditure). Data will be classified into the following cost components: direct medical costs (consultations, tests, medicines, and hospitalization, etc.), direct non-medical cost (transport and food during healthcare visits) and indirect costs (income and time lost). If data allow, costs will be stratified by socioeconomic status, sex, age, ethnicity, health condition, kind of health service sought and obtained, and regions (slum, non-slum, other). We will extract data on both relative and absolute difference in costs between slum/non-slum and where possible stratified by socio-economic status. This data extraction strategy will be piloted and adjusted, if necessary.

Quality assessment

Although quality assessment is not a mandatory step for scoping reviews, we will examine the methodological quality of the studies by using checklists recommended for costing and cost-effectiveness studies.

We will assess the quality of studies based on the Consolidated Health Economic Evaluation Reporting Standards (CHEERS).[44] We will also use the Tool to Estimate Patient's Costs (TBCA)[45] to evaluate methods applied to estimate costs and procedures adopted for interviewing patients. The

For peer review only - http://bmjopen.bmj.com/site/about/guidelines.xhtml

quality assessment will be focused on the methods reported in the studies through the assessment of the criteria indicated in Table 2.

Table 2. Items included in the quality assessment of the manuscripts

**Consolidated Health Economic Evaluation Reporting Standards**	Cost estimation methods
	Target population
	Setting and location
	Study perspective
	Sources used for resource quantities and unit costs
	Period for resources estimation; quantities and unit costs
	Methods for adjusting unit costs to the reporting year and performing currency conversion
	Values for main categories of estimated costs
	Choice of discount rate(s) used for costs and outcomes and why (studies with time horizon above one year are expected to discount rates to adjust costs estimates.)
	Time horizon over which costs and consequences are being evaluated and why
**Tool to Estimate Patient's Costs**	Patient interview procedures
	Methods used for valuing indirect costs

Collating, summarizing, and reporting the data

Costs reported before 2020 will be updated using USD inflation rates.[46] Costs reported in local currency will then be converted to US Dollars using exchange rates as reported in the OANDA website (OANDA, 2018). To allow comparison of results reported by different countries, costs will be presented in International Dollars ($) applying purchase power parity,[47] 2020 prices. The conversion to international dollars will apply World Bank indices.[48] Results will be presented as the average cost of health care, that is cost of illness and cost of seeking care, comparing slum dwellers vs other city residents. We will first estimate average total costs and average direct medical, non-medical and indirect costs. When available, we will also estimate disaggregated data on cost by health sector (public/private/NGO; Formal vs Informal), by wealth quintile or household assets, and by disease profile (Non-communicable diseases, communicable diseases, accidents and violence). The range of reported means across studies, unweighted average of means (with standard deviation), and the median of means (with interquartile range) will be presented for each costing category. The prevalence of CHE will be reported as range, unweighted average, and median. For studies reporting data from several countries, each country will be analysed as a separate observation. We will run a sensitivity analysis including only studies classified as good quality to evaluate uncertainties in our estimates.

We will report our review following the guideline Preferred Reporting Items for Systematic reviews and Meta-Analyses extension for Scoping Reviews (PRISMA-ScR) Checklist (Tricco *et al.*, 2018). Results will be synthesized and presented using a narrative and tables.

Ethics

Ethical approval is not required for scoping reviews.

For peer review only - http://bmjopen.bmj.com/site/about/guidelines.xhtml

Patient and public involvement

We formulated our research question based on the ARISE principle to generate evidence to promote accountability and responsiveness of slum-dwellers. Results generated by this review will potentially guide the decision-making process to improve access to health care and alleviate the economic impact of diseases on slum communities.

Our review team includes participants from all ARISE partners: Liverpool School of Tropical Medicine, University of York and University of Glasgow in UK; Brac University in Bangladesh; LVCT Health and African Population and Health Research Center in Kenya; COMAHS: University of Sierra Leone in Sierra Leone; The George Institute for Global Health and The Society for Promotion of Area Resource Centres in India. The review has a strong capacity strengthening element and all reviewers are completing the Cochrane interactive learning modules (https://training.cochrane.org/interactivelearning). The team is also meeting regularly to develop skills and ensure quality and consistency throughout the review.

Patients are not directly involved in the review process as this is based on secondary data.

Dissemination

We will disseminate the results of this review alongside the events organised by the ARISE hub, such as project meetings, seminars, webinars, and capacity building training programme. Additionally, we will produce policy briefs including key results of the review, knowledge gaps and priority actions for representatives of the slum communities, policy-makers and other stakeholders in the countries where the ARISE project is working (India, Bangladesh, Sierra Leone, Kenya).

This scoping review addresses a topic of interest to the Sustainable Development Goals (SDG), particularly SDG 3, Good Health and Wellbeing, and 10, Reduce Inequalities. Thus, our review will be disseminated at international conferences and will potentially contribute to formulating public policies to improve access to health care by slum dwellers.

The final manuscript will be submitted to an open-access international journal to reach a larger audience.

Contributions

The review team is distributed in three groups and participants were allocated to these groups according to their skills, experience in conducting systematic reviews and data analysis, and time availability. NTSF, HE, JL, EK, PPH, ZQ are part of the *core group* and are responsible for developing the main activities of the scoping review, such as screen and select the manuscripts, extract the data, and data analysis and writing the manuscript and reports. The second group comprises *new reviewers*. They will participate in the whole review process under the guidance and mentorship of the participants from the core group. AA, IM, FM, RK, SS, JS, VS, SG, and IC are part of the *new reviewer* group. The third group comprises the *advisory group* and includes senior researchers with expertise in health economics, urban health, and slums. They will guide the core team on the data analysis process, definitions of key-word, such as slums and economic impact, also acting as tie-breakers on the occasion of disagreements in the inclusion and data extraction stages of the review. BM, VR, RT, NF, WA and SM are part of this group.

This protocol was written by NTSF and HE with contributions and approval from all co-authors.

Funding sources/sponsors: this review is supported by ARISE UK Research and Innovation's Global Challenges Research Fund (ES/S00811X/1, ARISE's Metrics, Epidemiology and Economics work

For peer review only - http://bmjopen.bmj.com/site/about/guidelines.xhtml

package group/University of York). EK also acknowledges support from the Medical Research Council and Scottish Government Chief Scientist Office funding (MC_UU_12017/13, SPHSU13). The funders had or will have no role in the development of this protocol, the collection and analyses, or interpretation of results, or in the writing or publication of the review's results.

Competing interests: None declared.

Acknowledgment: We thank Melissa Harden, the information specialist form the Centre for Reviews and Dissemination at the University of York, for developing the search strategy.

Data availability: No data have been generated as this is a study protocol.

Figure legend:

Figure 1. Inclusion and exclusion pathway of the scoping review.

For peer review only

For peer review only - http://bmjopen.bmj.com/site/about/guidelines.xhtml

References

1. United Nations. World Urbanization Prospects. The 2018 revision. New York; 2019.

2. United Nations. The Millennium Development Goals Report [Internet]. United Nations. 2015. Available from: https://visit.un.org/millenniumgoals/2008highlevel/pdf/MDG_Report_2008_Addendum.pdf

3. The World Bank. World Bank Country and Lending Groups [Internet]. 2020 [cited 2020 Sep 22]. Available from: https://datahelpdesk.worldbank.org/knowledgebase/articles/906519

4. Chen M, Zhang H, Liu W, Zhang W. The Global Pattern of Urbanization and Economic Growth : Evidence from the Last Three Decades. Plos O. 2014;9(8).

5. Ezeh A, Oyebode O, Satterthwaite D, Chen YF, Ndugwa R, Sartori J, et al. The history, geography, and sociology of slums and the health problems of people who live in slums. Lancet [Internet]. 2017;389(10068):547–58. Available from: http://dx.doi.org/10.1016/S0140-6736(16)31650-6

6. Shawar YR, Crane LG. Generating global political priority for urban health : the role of the urban health epistemic community. 2017;(June):1161–73.

7. Mberu BU, Haregu TN, Kyobutungi C, Ezeh AC. Health and health-related indicators in slum , rural , and urban communities : a comparative analysis. 2016;1:1–13.

8. Fink G, Günther I, Hill K. Slum Residence and Child Health in Developing Countries. Demography. 2014;51:1175–1197.

9. National Institute of Population Research and Training. Bangladesh Urban Health Survey 2013. Final report. 2015.

10. Elsey H, Thomson DR, Lin RY, Maharjan U, Agarwal S, Newell J. Addressing Inequities in Urban Health : Do Decision-Makers Have the Data They Need ? Report from the Urban Health Data Special Session at International Conference on Urban Health Dhaka 2015. J Urban Heal [Internet]. 2016;93(3):526–37. Available from: http://dx.doi.org/10.1007/s11524-016-0046-9

11. Lucci P, Bhatkal T. Monitoring progress on urban poverty Are current data and indicators fit for. 2014;(October).

12. Patil RR. Social Work in Public Health Urbanization as a Determinant of Health : A Socioepidemiological Perspective Urbanization as a Determinant of Health : A Socioepidemiological Perspective. 2014;1918.

13. Kimani-Murage EW, Fotso JC, Egondi T, Abuya B, Elungata P, Ziraba AK, et al. Trends in childhood mortality in Kenya: The urban advantage has seemingly been wiped out. Heal Place [Internet]. 2014;29:95–103. Available from: http://dx.doi.org/10.1016/j.healthplace.2014.06.003

14. Fotso JC, Madise N, Baschieri A, Cleland J, Zulu E, Kavao Mutua M, et al. Child growth in urban deprived settings: Does household poverty status matter? At which stage of child development? Heal Place [Internet]. 2012;18(2):375–84. Available from: http://dx.doi.org/10.1016/j.healthplace.2011.12.003

15. Ogunsina MA, Anumah FO. Prevalence and correlates of hypertension and diabetes mellitus in an urban community in North-Western Nigeria. 2018;8688:1–7.

16. Mberu B, Wamukoya M, Oti S, Kyobutungi C. Trends in Causes of Adult Deaths among the Urban Poor : Evidence from Nairobi Urban Health and Demographic Trends in Causes of Adult

For peer review only - http://bmjopen.bmj.com/site/about/guidelines.xhtml

Deaths among the Urban Poor : Evidence from Nairobi Urban Health and Demographic Surveillance System , 2003 – 2012. J Urban Heal Urban Heal. 2015;(March):2003–12.

17. Govindaraj R, Raju D, Secci F, Chowdhury S, Frere J-J. Health and nutrition in Bangladesh: Social Determinants and Governance. World Bank Group; 2018.

18. Peters R, Amugsi DA, Mberu B, Ensor T, Hill AJ, Newell JN, et al. Nutrition transition , overweight and obesity among rural-to-urban migrant women in Kenya Nutrition transition , overweight and obesity among rural-to-urban migrant women in Kenya. 2019;(June).

19. Ofori-asenso R, Agyeman AA, Laar A, Boateng D. Overweight and obesity epidemic in Ghana — a systematic review and meta-analysis. BMC Public Health [Internet]. 2016; Available from: http://dx.doi.org/10.1186/s12889-016-3901-4

20. Williams CT, Grier SA, Marks AS. " Coming to Town " : The Impact of Urbanicity , Cigarette Advertising , and Network Norms on the Smoking Attitudes of Black Women in Cape Town , South Africa. 2008;85(4):472–85.

21. Rawal LB, Biswas T, Khandker NN, Saha SR, Chowdhury MMB, Khan ANS, et al. Non-communicable disease (NCD) risk factors and diabetes among adults living in slum areas of Dhaka, Bangladesh. PLoS One. 2017;12(10):1–15.

22. Gitatui M, Kimani S, Muniu S, Okube O, Kimani S. Determinants of harmful use of alcohol among urban slum dwelling adults in Kenya. 2019;19(4):2906–25.

23. Adhikari TB, Rijal A, Kallestrup P, Neupane D. Alcohol consumption pattern in western Nepal : findings from the COBIN baseline survey. 2019;(September).

24. Fisher M. A theory of public wellbeing. BMC Public Health. 2019;19(1):1–12.

25. Albis MLF, Bhadra SK, Chin B. Impact evaluation of contracting primary health care services in urban Bangladesh. BMC Health Serv Res [Internet]. 2019;854. Available from: http://dx.doi.org/10.1186/s12913-019-4406-5

26. Angeles G, Ahsan KZ. Reducing Inequity in Urban Health : Have the Intra-urban Differentials in Reproductive Health Service Utilization and Child Nutritional Outcome Narrowed in Bangladesh ? Reducing Inequity in Urban Health : Have the Intra-urban Differentials in Reproductive. 2018;(September).

27. Van Belle S, Affun-Adegbulu C, Soors W, Srinivas PN, Hegel G, Van Damme W, et al. COVID-19 and informal settlements: An urgent call to rethink urban governance. Int J Equity Health. 2020;19(1):1–2.

28. Bartlett JD, Vivrette R. Ways to Promote Children ' s Resilience to the COVID-19 Pandemic. 2020.

29. Mirzoev T, Poudel AN, Gissing S, Thuy T, Doan D, Ferdous T, et al. Is evidence-informed urban health planning a myth or reality ? Lessons from a qualitative assessment in three Asian cities. 2019;(October):773–83.

30. Otieno PO, Wambiya EOA, Mohamed SM, Mutua MK, Kibe PM, Mwangi B, et al. Access to primary healthcare services and associated factors in urban slums in. 2020;1–9.

31. Adams AM, Islam R, Yusuf SS, Panasci A, Crowell N. Healthcare seeking for chronic illness among adult slum dwellers in Bangladesh : A descriptive cross-sectional study in two urban settings. PLoS One [Internet]. 2020;1–18. Available from: http://dx.doi.org/10.1371/journal.pone.0233635

For peer review only - http://bmjopen.bmj.com/site/about/guidelines.xhtml

32. Buigut S, Ettarh R, Amendah DD. Catastrophic health expenditure and its determinants in Kenya slum communities. Int J Equity Health [Internet]. 2015;14(1):1–12. Available from: ???

33. Biswas S. India coronavirus: “More than half of Mumbai slum-dwellers had Covid-19” [Internet]. BBC News. 2020 [cited 2020 Sep 29]. Available from: https://www.bbc.co.uk/news/world-asia-india-53576653

34. The Guardian. Half of Mumbai’s slum residents have had coronavirus – study [Internet]. 2020 [cited 2020 Sep 29]. Available from: https://www.theguardian.com/world/2020/jul/29/half-of-mumbais-slum-residents-have-had-coronavirus-study

35. ARISE. ARISE, Accountability in Urban Health [Internet]. 2019 [cited 2020 Jun 6]. Available from: http://www.ariseconsortium.org/

36. Arksey H, O’Malley L. Scoping studies: Towards a methodological framework. Int J Soc Res Methodol Theory Pract. 2005;8(1):19–32.

37. Levac D, Colquhoun H, O’Brien KK. Scoping studies: Advancing the methodology. Implement Sci. 2010;5(1):1–9.

38. The Joanna Briggs Institute. The Systematic Review of Economic Evaluation Evidence [Internet]. The Joanna Briggs Institute; 2014. p. 1–40. Available from: www.joannabriggs.org

39. UN-Habitat. Slums : Some Definitions Slums : Neither Bricks nor Mortar , Non-Durable Housing [Internet]. United Nations; 2007. p. 3–4. Available from: https://mirror.unhabitat.org/documents/media_centre/sowcr2006/SOWCR 5.pdf

40. Xu K, Evans DB, Kawabata K, Zeramdini R, Klavus J, Murray CJL. Household catastrophic health expenditure : a multicountry analysis. Lancet. 2003;362:111–7.

41. Kirch W, editor. Direct Costs. In: Encyclopedia of Public Health [Internet]. Dordrecht: Springer Netherlands; 2008. p. 267. Available from: https://doi.org/10.1007/978-1-4020-5614-7_799

42. Tricco AC, Lillie E, Zarin W, O’Brien KK, Colquhoun H, Levac D, et al. PRISMA extension for scoping reviews (PRISMA-ScR): Checklist and explanation. Ann Intern Med. 2018;169(7):467–73.

43. Ouzzani M, Hammady H, Fedorowicz Z, Elmagarmid A. Rayyan-a web and mobile app for systematic reviews. Syst Rev. 2016;5(1):1–10.

44. Husereau D, Drummond M, Petrou S, Carswell C, Moher D, Greenberg D. Consolidated Health Economic Evaluation Reporting Standards (CHEERS)--Explanation and elaboration: A report of the ISPOR Health Economic Evaluations Publication Guidelines Task Force. Value Heal. 2013;16:231–50.

45. World Health Organization. The tool to estimate patients’ costs. 2008.

46. International Monetary Fund. International Monetary Fund [Internet]. 2020. Available from: https://www.imf.org/en/Data

47. Kirch W, editor. Purchasing Power Parity. In: Encyclopedia of Public Health [Internet]. Dordrecht: Springer Netherlands; 2008. p. 1220. Available from: https://doi.org/10.1007/978-1-4020-5614-7_2880

48. The World Bank. World Development Indicators: Exchange rates and prices [Internet]. 2017 [cited 2016 Nov 1]. Available from: http://wdi.worldbank.org/table/4.16

For peer review only

For peer review only - http://bmjopen.bmj.com/site/about/guidelines.xhtml

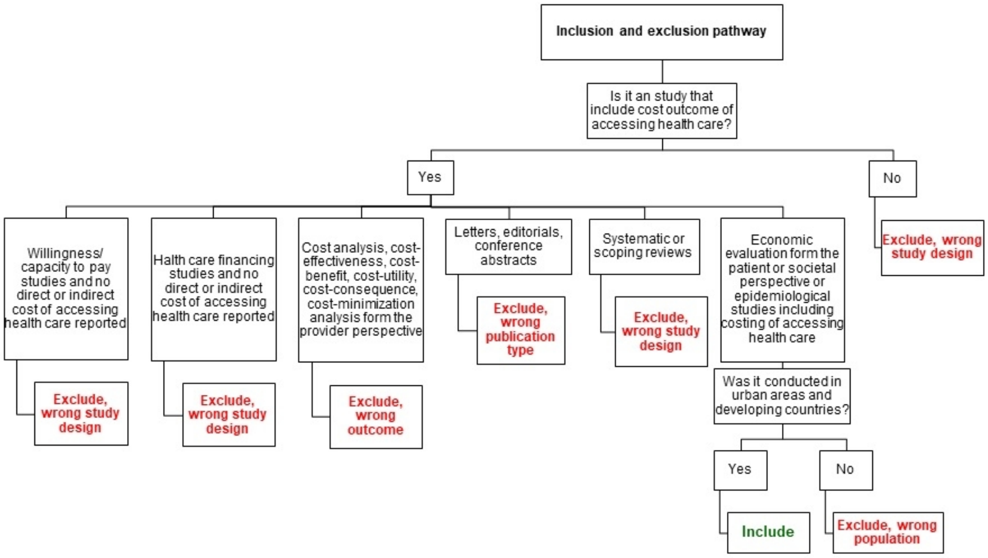

Figure 1. Inclusion and exclusion pathway of the scoping review.

235x145mm (96 x 96 DPI)

Supplementary material

The economics of health-care for slum-dwellers

Literature search

The aim of the search strategy was to identify studies examining the costs of healthcare for slum dwellers or city residents in low- and middle-income countries (LMIC). A search strategy was developed by an information specialist using Ovid MEDLINE. Search terms were gathered together for the following four key concepts: slum dwellers, urban areas, healthcare costs and LMIC. Both textword and subject heading searches for each concept were included in the strategy. Retrieval was limited to publications within the last 10 years. No langauage restrictions were applied. The final search strategy for MEDLINE was agreed by the review team and then translated as appropriate for the other databases and resources.

Databases containing literature from the fields of health, economics and social science were searched on 22nd June 2020: MEDLINE (Ovid), Embase (Ovid), EconLit (Ovid), Science Citation Index (Web of Science), Social Science Citation Index (Web of Science) and Global Index Medicus. In addition, the following resources containing grey literature such as theses and dissertations, working papers and reports were searched: Proquest Dissertations and Theses (A&I), Econpapers, OpenGrey, the World Bank website and the Organisation for Economic Co-operation and Development (OECD) website.

EndNote was used for reference management and duplicate removal. The search strategies for all databases and resources can be found in the appendix.

For peer review only

Appendix

The search strategies for MEDLINE and EMBASE incorporated a low- and middle-income countries (LMIC) search filter developed by the Effective Practice and Organisation of Care (EPOC) Cochrane group.[1] Where possible, this filter was translated for use in the other databases searched.

MEDLINE ALL
via Ovid http://ovidsp.ovid.com/
1946 to June 19, 2020
Search date: 22nd June 2020
Records retrieved: 2368
POPULATION
1 Urban Population/ (58856)
2 Cities/ (19180)
3 Urban Health/ (17793)
4 Urban Health Services/ (3613)
5 Hospitals, Urban/ (7341)
6 urban$.ti,ab. (156001)
7 (city or cities or megacity or megacities or metropolitan).ti,ab. (169349)
8 (metropolis or megalopolis or municipal$ or conurbation$ or suburb$ or town or towns or township$ or borough$ or barrio or barrios).ti,ab. (81323)
9 ((dense$ or density or high$ or large$ or heavy or heavily) adj3 (populat$ or populous) adj3 (area$ or settlement$ or district$ or neighbourhood$ or communit$)).ti,ab. (3451)
10 or/1-9 (390327)
11 (afghanistan or albania or algeria or american samoa or angola or "antigua and barbuda" or antigua or barbuda or argentina or armenia or armenian or aruba or azerbaijan or bahrain or bangladesh or barbados or republic of belarus or belarus or byelarus or belorussia or byelorussian or belize or british honduras or benin or dahomey or bhutan or bolivia or "bosnia and herzegovina" or bosnia or herzegovina or botswana or bechuanaland or brazil or brasil or bulgaria or burkina faso or burkina fasso or upper volta or burundi or urundi or cabo verde or cape verde or cambodia or kampuchea or khmer republic or cameroon or cameron or cameroun or central african republic or ubangi shari or chad or chile or china or colombia or comoros or comoro islands or iles comores or mayotte or democratic republic of the congo or democratic republic congo or congo or zaire or costa rica or "cote d'ivoire" or "cote d' ivoire" or cote divoire or cote d ivoire or ivory coast or croatia or cuba or cyprus or czech republic or czechoslovakia or djibouti or french somaliland or dominica or dominican republic or ecuador or egypt or united arab republic or el salvador or equatorial guinea or spanish guinea or eritrea or estonia or eswatini or swaziland or ethiopia or fiji or gabon or gabonese republic or gambia or "georgia (republic)" or georgian or ghana or gold coast or gibraltar or greece or grenada or guam or guatemala or guinea or guinea bissau or guyana or british guiana or haiti or hispaniola or honduras or hungary or india or indonesia or timor or iran or iraq or isle of man or jamaica or jordan or kazakhstan or kazakh or kenya or "democratic people's republic of korea" or republic of korea or north korea or south korea or korea or kosovo or kyrgyzstan or kirghizia or kirgizstan or kyrgyz republic or kirghiz or laos or lao pdr or "lao people's democratic republic" or latvia or lebanon or lebanese republic or lesotho or basutoland or liberia or libya or libyan arab jamahiriya or lithuania or macau or macao or "macedonia (republic)" or macedonia or madagascar or malagasy republic or malawi or nyasaland or malaysia or malay federation or malaya federation or maldives or indian ocean islands or indian ocean or mali or malta or micronesia or federated states of micronesia or kiribati or marshall islands or nauru or northern mariana islands or palau or tuvalu or mauritania or mauritius or mexico or moldova or moldovian or mongolia or montenegro or morocco or ifni or mozambique or portuguese east africa or myanmar or burma or namibia or nepal or netherlands antilles or nicaragua or niger or nigeria or oman or muscat or pakistan or panama or

For peer review only

papua new guinea or new guinea or paraguay or peru or philippines or philipines or phillipines or phillippines or poland or "polish people's republic" or portugal or portuguese republic or puerto rico or romania or russia or russian federation or ussr or soviet union or union of soviet socialist republics or rwanda or ruanda or samoa or pacific islands or polynesia or samoan islands or navigator island or navigator islands or "sao tome and principe" or saudi arabia or senegal or serbia or seychelles or sierra leone or slovakia or slovak republic or slovenia or melanesia or solomon island or solomon islands or norfolk island or norfolk islands or somalia or south africa or south sudan or sri lanka or ceylon or "saint kitts and nevis" or "st. kitts and nevis" or saint lucia or "st. lucia" or "saint vincent and the grenadines" or saint vincent or "st. vincent" or grenadines or sudan or suriname or surinam or dutch guiana or netherlands guiana or syria or syrian arab republic or tajikistan or tadjikistan or tadzhikistan or tadzhik or tanzania or tanganyika or thailand or siam or timor leste or east timor or togo or togolese republic or tonga or "trinidad and tobago" or trinidad or tobago or tunisia or turkey or "turkey (republic)" or turkmenistan or turkmen or uganda or ukraine or uruguay or uzbekistan or uzbek or vanuatu or new hebrides or venezuela or vietnam or viet nam or middle east or west bank or gaza or palestine or yemen or yugoslavia or zambia or zimbabwe or northern rhodesia or global south or africa south of the sahara or sub-saharan africa or subsaharan africa or africa, central or central africa or africa, northern or north africa or northern africa or magreb or maghrib or sahara or africa, southern or southern africa or africa, eastern or east africa or eastern africa or africa, western or west africa or western africa or west indies or indian ocean islands or caribbean or central america or latin america or "south and central america" or south america or asia, central or central asia or asia, northern or north asia or northern asia or asia, southeastern or southeastern asia or south eastern asia or southeast asia or south east asia or asia, western or western asia or europe, eastern or east europe or eastern europe or developing country or developing countries or developing nation? or developing population? or developing world or less developed countr* or less developed nation? or less developed population? or less developed world or lesser developed countr* or lesser developed nation? or lesser developed population? or lesser developed world or under developed countr* or under developed nation? or under developed population? or under developed world or underdeveloped countr* or underdeveloped nation? or underdeveloped population? or underdeveloped world or middle income countr* or middle income nation? or middle income population? or low income countr* or low income nation? or low income population? or lower income countr* or lower income nation? or lower income population? or underserved countr* or underserved nation? or underserved population? or underserved world or under served countr* or under served nation? or under served population? or under served world or deprived countr* or deprived nation? or deprived population? or deprived world or poor countr* or poor nation? or poor population? or poor world or poorer countr* or poorer nation? or poorer population? or poorer world or developing economy* or developing economies or less developed econom* or lesser developed econom* or under developed econom* or underdeveloped econom* or middle income econom* or low income econom* or lower income econom* or low gdp or low gnp or low gross domestic or low gross national or lower gdp or lower gnp or lower gross domestic or lower gross national or lmic or lmics or third world or lami countr* or transitional countr* or emerging economy or emerging economies or emerging nation?).ti,ab,sh,kf. (1907989)

12 (afghan or afghans or afghani or albanian? algerian? or american samoan? or angolan? or antiguan? or barbudan? or argentine? or argentinian? or argentinean? or armenian? or aruban? or azerbaijani? or bahraini? or bangladeshi? or bangalees or bajan? or belarusian? or byelorussian? or belizean? or beninese? or bhutanese or bolivian? or bosnian? or botswana or batswana or brazilian? or brasilian? or bulgarian? or burkinabe or burkinese or burundian? or cape verdean? or cabo verdean? or cambodian? or khmer or cameroonian? or central african? or chadian? or chilean? or chinese or colombian? or comorian? or congolese or costa rican? or ivorian? or croatian? or cuban? or cypriot? or czech? or djiboutian? or dominican? or ecuadorian? or egyptian? or salvadoran? or equatorial guinean? or equatoguinean? or eritrean? or estonian? or swazi? or swati? or ethiopian? or fijian or gabonese or gabonaise or gambian? or georgian? or ghanaian? or gibraltarian? or greek?

For peer review only

or grenadian? or guamanian? or guatemalan? or guinean? or bissau guinean? or guyanese or haitian? or honduran? or hungarian? or indian? or indonesian? or iranian? or iraqian? or iraqi? or manx or jamaican? or jordanian? or kazakhstani? or kenyan? or kirabati or kirabatian? or north korean? or korean? or kosovar? or kosovan? or kyrgyz* or lao or laotian? or latvian? or lebanese or lesothan? or lesothonian? or mosotho or basotho or liberian? or libyan? or lithuanian? or macanese or macedonian? or malagasy or madagascan? or malawian? or malaysian? or maldivian? or malian? or maltese or marshallese? or mauritanian? or mauritian? or mexican? or micronesian? or moldovan? or mongolian? or mongol or montenegrin? or moroccan? or mozambican? or burmese or myanma or namibian? or nauruan? or nepali or nepalese or netherlands antillean? or nicaraguan? or nigerien? or nigerian? or northern mariana islander? or mariana? or omani? or pakistani? or palauan? or panamanian? or papua new guinean? or paraguayan? or peruvian? or philippine? or philipine? or phillipine? or phillippine? or filipino? or filipina? or polish or pole or poles or portuguese or puerto rican? or romanian? or russian? or soviet people or soviet population or rwandan? or rwandese or ruandan? or ruandese or samoan? or sao tomean? or santomean? or saudi arabian? or saudi? or senegalese or serbian? or montenegrin? or seychellois or seychelloise? or sierra leonean? or slovak? or slovene? or solomon islander? or somali? or south african? or south sudanese or sri lankan? or ceylonese or kittitian? or nevisian? or saint lucian? or vincentian? or sudanese or surinamese? or syrian? or tajik? or tajikistani? or tanzanian? or tanganyikan? or thai or timorese? or togolese or tongan? or trinidadian? or tobagonian? or tunisian? or turk? or turkish or turkmen? or tuvaluan? or ugandan? or ukrainian? or uruguayan? or uzbek? or vanuatu* or venezuelan? or vietnamese or yemeni? or yemenite? or yemenese or yugoslav? or yugoslavian? or zambian? or zimbabwean?).ti,ab,sh,kf. (834927)
13 11 or 12 (2269396)
14 10 and 13 (169934)

COSTS
15 poverty areas/ (6047)
16 (slum or slums or ghetto or ghettos or shanty$ or shanties or shack$ or favela$).ti,ab. (5200)
17 (informal$ adj3 settlement$).ti,ab. (558)
18 ((poverty or impoverish$) adj3 (area$ or settlement$)).ti,ab. (881)
19 ((precarious$ or irregular$) adj3 settlement$).ti,ab. (8)
20 (squatter$ adj3 (area$ or settlement$)).ti,ab. (227)
21 or/15-20 (11156)
22 "cost of illness"/ (27054)
23 exp Health Care Costs/ (64997)
24 Health Expenditures/ (20173)
25 exp Financing, Personal/ (6053)
26 Catastrophic Illness/ec [Economics] (550)
27 (cost$ adj2 (illness$ or sickness$ or disease$)).ti,ab. (5084)
28 (cost$ adj3 (care or health or healthcare or medical$)).ti,ab. (76646)
29 (expenditure$ adj3 (care or health or healthcare or medical$)).ti,ab. (13957)
30 ((direct or indirect) adj2 (cost$ or expenditure$)).ti,ab. (15577)
31 out of pocket.ti,ab. (5698)
32 (OOP adj3 (cost$ or expense$ or expenditure$ or financ$ or pay$ or paid or spend$)).ti,ab. (505)
33 OOPE.ti,ab. (74)
34 ((personal or individual$ or patient$ or family$ or families or household$) adj3 (budget$ or cost$ or expense$ or expenditure$ or financ$ or pay$ or paid or spend$) adj3 (care or health or healthcare or medical$)).ti,ab. (9512)
35 (catastroph$ adj10 (cost$ or expense$ or expenditure$ or financ$ or pay$ or paid or spend$)).ti,ab. (1323)

For peer review only

36 ((medical$ or health) adj3 impoverish$).ti,ab. (232)
37 (burden$ adj3 (cost$ or expenditure$ or economic$ or financ$) adj3 (care or health or healthcare or medical$)).ti,ab. (4485)
38 ((willing$ or unwilling$) adj3 pay$ adj3 (care or health or healthcare or medical$)).ti,ab. (323)
39 ((afford$ or unafford$) adj5 (cost$ or pay$)).ti,ab. (2273)
40 ((able or ability or capacit$ or unable or inability) adj2 pay$).ti,ab. (1460)
41 or/22-40 (185296)
42 14 and 41 (3361)
43 21 and 41 (267)
44 42 or 43 (3541)

TYPE OF STUDY
45 letter.pt. (1084673)
46 editorial.pt. (532516)
47 historical article.pt. (358723)
48 or/45-47 (1956361)
49 44 not 48 (3506)
50 exp animals/ not humans/ (4709143)
51 49 not 50 (3497)
52 limit 51 to yr="2010 -Current" (2368)

Key:
/ = subject heading (MeSH heading)
exp = exploded subject heading (MeSH heading)
/ec = economics subheading
$ = truncation
* = truncation
" " = exact phrase search
? = stands for zero or one characters within a word or at the end of a word
ti,ab = terms in either title or abstract fields
kf = terms in author keywords field
sh = terms in the subject heading field
pt = terms in publication type field
adj3 = terms within three words of each other (any order)
adj = terms next to each other in the same order

Embase
via Ovid http://ovidsp.ovid.com/
1974 to 2020 June 19
Search date: 22nd June 2020
Records retrieved: 2958

1 urban area/ (60321)
2 urban population/ (44806)
3 urban health/ (1037)
4 urban hospital/ (303)
5 city/ (38692)
6 suburban area/ (2548)
7 suburban population/ (670)
8 (city or cities or megacity or megacities or metropolitan).ti,ab. (219736)

For peer review only

9 (metropolis or megalopolis or municipal$ or conurbation$ or suburb$ or town or towns or township$ or borough$ or barrio or barrios).ti,ab. (100040)
10 ((dense$ or density or high$ or large$ or heavy or heavily) adj3 (populat$ or populous) adj3 (area$ or settlement$ or district$ or neighbourhood$ or communit$)).ti,ab. (4243)
11 urban$.ti,ab. (200356)
12 or/1-11 (486662)
13 (afghanistan or albania or algeria or american samoa or angola or "antigua and barbuda" or antigua or barbuda or argentina or armenia or armenian or aruba or azerbaijan or bahrain or bangladesh or barbados or republic of belarus or belarus or byelarus or belorussia or byelorussian or belize or british honduras or benin or dahomey or bhutan or bolivia or "bosnia and herzegovina" or bosnia or herzegovina or botswana or bechuanaland or brazil or brasil or bulgaria or burkina faso or burkina fasso or upper volta or burundi or urundi or cabo verde or cape verde or cambodia or kampuchea or khmer republic or cameroon or cameron or cameroun or central african republic or ubangi shari or chad or chile or china or colombia or comoros or comoro islands or iles comores or mayotte or democratic republic of the congo or democratic republic congo or congo or zaire or costa rica or "cote d'ivoire" or "cote d' ivoire" or cote divoire or cote d ivoire or ivory coast or croatia or cuba or cyprus or czech republic or czechoslovakia or djibouti or french somaliland or dominica or dominican republic or ecuador or egypt or united arab republic or el salvador or equatorial guinea or spanish guinea or eritrea or estonia or eswatini or swaziland or ethiopia or fiji or gabon or gabonese republic or gambia or "georgia (republic)" or georgian or ghana or gold coast or gibraltar or greece or grenada or guam or guatemala or guinea or guinea bissau or guyana or british guiana or haiti or hispaniola or honduras or hungary or india or indonesia or timor or iran or iraq or isle of man or jamaica or jordan or kazakhstan or kazakh or kenya or "democratic people's republic of korea" or republic of korea or north korea or south korea or korea or kosovo or kyrgyzstan or kirghizia or kirgizstan or kyrgyz republic or kirghiz or laos or lao pdr or "lao people's democratic republic" or latvia or lebanon or lebanese republic or lesotho or basutoland or liberia or libya or libyan arab jamahiriya or lithuania or macau or macao or "macedonia (republic)" or macedonia or madagascar or malagasy republic or malawi or nyasaland or malaysia or malay federation or malaya federation or maldives or indian ocean islands or indian ocean or mali or malta or micronesia or federated states of micronesia or kiribati or marshall islands or nauru or northern mariana islands or palau or tuvalu or mauritania or mauritius or mexico or moldova or moldovian or mongolia or montenegro or "montenegro (republic)" or morocco or ifni or mozambique or portuguese east africa or myanmar or burma or namibia or nepal or netherlands antilles or nicaragua or niger or nigeria or oman or muscat or pakistan or panama or papua new guinea or new guinea or paraguay or peru or philippines or philipines or phillipines or phillippines or poland or "polish people's republic" or portugal or portuguese republic or puerto rico or romania or russia or russian federation or ussr or soviet union or union of soviet socialist republics or rwanda or ruanda or samoa or pacific islands or polynesia or samoan islands or navigator island or navigator islands or "sao tome and principe" or saudi arabia or senegal or serbia or seychelles or sierra leone or slovakia or slovak republic or slovenia or melanesia or solomon island or solomon islands or norfolk island or norfolk islands or somalia or south africa or south sudan or sri lanka or ceylon or "saint kitts and nevis" or "st. kitts and nevis" or saint lucia or "st. lucia" or "saint vincent and the grenadines" or saint vincent or "st. vincent" or grenadines or sudan or suriname or surinam or dutch guiana or netherlands guiana or syria or syrian arab republic or tajikistan or tadjikistan or tadzhikistan or tadzhik or tanzania or tanganyika or thailand or siam or timor leste or east timor or togo or togolese republic or tonga or "trinidad and tobago" or trinidad or tobago or tunisia or turkey or "turkey (republic)" or turkmenistan or turkmen or uganda or ukraine or uruguay or uzbekistan or uzbek or vanuatu or new hebrides or venezuela or vietnam or viet nam or middle east or west bank or gaza or palestine or yemen or yugoslavia or zambia or zimbabwe or northern rhodesia or global south or africa south of the sahara or "sub saharan africa" or subsaharan africa or africa, central or central africa or africa, northern or north africa or northern africa or magreb or maghrib or sahara or africa, southern or southern africa or africa, eastern or east africa or

For peer review only

eastern africa or africa, western or west africa or western africa or west indies or indian ocean islands or caribbean region or caribbean islands or caribbean or central america or latin america or "south and central america" or south america or asia, central or central asia or asia, northern or north asia or northern asia or asia, southeastern or southeastern asia or south eastern asia or southeast asia or south east asia or asia, western or western asia or europe, eastern or east europe or eastern europe or developing country or developing countries or developing nation? or developing population? or developing world or less developed countr* or less developed nation? or less developed population? or less developed world or lesser developed countr* or lesser developed nation? or lesser developed population? or lesser developed world or under developed countr* or under developed nation? or under developed population? or under developed world or underdeveloped countr* or underdeveloped nation? or underdeveloped population? or underdeveloped world or middle income countr* or middle income nation? or middle income population? or low income countr* or low income nation? or low income population? or lower income countr* or lower income nation? or lower income population? or underserved countr* or underserved nation? or underserved population? or underserved world or under served countr* or under served nation? or under served population? or under served world or deprived countr* or deprived nation? or deprived population? or deprived world or poor countr* or poor nation? or poor population? or poor world or poorer countr* or poorer nation? or poorer population? or poorer world or developing econom* or less developed econom* or lesser developed econom* or under developed econom* or underdeveloped econom* or middle income econom* or low income econom* or lower income econom* or low gdp or low gnp or low gross domestic or low gross national or lower gdp or lower gnp or lower gross domestic or lower gross national or lmic or lmics or third world or lami countr* or transitional countr* or emerging economies or emerging economy or emerging nation?).ti,ab,sh,kw. (2145013)

14 (afghan or afghans or afghani or albanian? algerian? or american samoan? or angolan? or antiguan? or barbudan? or argentine? or argentinian? or argentinean? or armenian? or aruban? or azerbaijani? or bahraini? or bangladeshi? or bangalees or bajan? or belarusian? or byelorussian? or belizean? or beninese? or bhutanese or bolivian? or bosnian? or botswana or batswana or brazilian? or brasilian? or bulgarian? or burkinabe or burkinese or burundian? or cape verdean? or cabo verdean? or cambodian? or khmer or cameroonian? or central african? or chadian? or chilean? or chinese or colombian? or comorian? or congolese or costa rican? or ivorian? or croatian? or cuban? or cypriot? or czech? or djiboutian? or dominican? or ecuadorian? or egyptian? or salvadoran? or equatorial guinean? or equatoguinean? or eritrean? or estonian? or swazi? or swati? or ethiopian? or fijian or gabonese or gabonaise or gambian? or georgian? or ghanaian? or gibraltarian? or greek? or grenadian? or guamanian? or guatemalan? or guinean? or bissau guinean? or guyanese or haitian? or honduran? or hungarian? or indian? or indonesian? or iranian? or iraqian? or iraqi? or manx or jamaican? or jordanian? or kazakhstani? or kenyan? or kirabati or kirabatian? or north korean? or korean? or kosovar? or kosovan? or kyrgyz* or lao or laotian? or latvian? or lebanese or lesothan? or lesothonian? or mosotho or basotho or liberian? or libyan? or lithuanian? or macanese or macedonian? or malagasy or madagascan? or malawian? or malaysian? or maldivian? or malian? or maltese or marshallese? or mauritanian? or mauritian? or mexican? or micronesian? or moldovan? or mongolian? or mongol or montenegrin? or moroccan? or mozambican? or burmese or myanma or namibian? or nauruan? or nepali or nepalese or netherlands antillean? or nicaraguan? or nigerien? or nigerian? or northern mariana islander? or mariana? or omani? or pakistani? or palauan? or panamanian? or papua new guinean? or paraguayan? or peruvian? or philippine? or philipine? or phillipine? or phillippine? or filipino? or filipina? or polish or pole or poles or portuguese or puerto rican? or romanian? or russian? or soviet people or soviet population or rwandan? or rwandese or ruandan? or ruandese or samoan? or sao tomean? or santomean? or saudi arabian? or saudi? or senegalese or serbian? or montenegrin? or seychellois or seychelloise? or sierra leonean? or slovak? or slovene? or solomon islander? or somali? or south african? or south sudanese or sri lankan? or ceylonese or kittitian? or nevisian? or saint lucian? or vincentian? or

For peer review only

sudanese or surinamese? or syrian? or tajik? or tajikistani? or tanzanian? or tanganyikan? or thai or timorese? or togolese or tongan? or trinidadian? or tobagonian? or tunisian? or turk? or turkish or turkmen? or tuvaluan? or ugandan? or ukrainian? or uruguayan? or uzbek? or vanuatu* or venezuelan? or vietnamese or yemeni? or yemenite? or yemenese or yugoslav? or yugoslavian? or zambian? or zimbabwean?).ti,ab,sh,kw. (1119374)
15 13 or 14 (2730608)
16 12 and 15 (203906)
17 (slum or slums or ghetto or ghettos or shanty$ or shanties or shack$ or favela$).mp. (5658)
18 (informal$ adj3 settlement$).mp. (628)
19 ((poverty or impoverish$) adj3 (area$ or settlement$)).mp. (1075)
20 ((precarious$ or irregular$) adj3 settlement$).mp. (12)
21 (squatter$ adj3 (area$ or settlement$)).mp. (207)
22 or/17-21 (7364)
23 "cost of illness"/ (19139)
24 exp "health care cost"/ (289949)
25 health economics/ (32736)
26 willingness to pay/ (695)
27 catastrophic health expenditure/ (32)
28 out of pocket/ (3)
29 (cost$ adj2 (illness$ or sickness$ or disease$)).ti,ab. (7680)
30 (cost$ adj3 (care or health or healthcare or medical$)).ti,ab. (114334)
31 (expenditure$ adj3 (care or health or healthcare or medical$)).ti,ab. (18625)
32 ((direct or indirect) adj2 (cost$ or expenditure$)).ti,ab. (26279)
33 out of pocket.ti,ab. (8054)
34 (OOP adj3 (cost$ or expense$ or expenditure$ or financ$ or pay$ or paid or spend$)).ti,ab. (797)
35 OOPE.ti,ab. (95)
36 ((personal or individual$ or patient$ or family$ or families or household$) adj3 (budget$ or cost$ or expense$ or expenditure$ or financ$ or pay$ or paid or spend$) adj3 (care or health or healthcare or medical$)).ti,ab. (14828)
37 (catastroph$ adj10 (cost$ or expense$ or expenditure$ or financ$ or pay$ or paid or spend$)).ti,ab. (1521)
38 ((medical$ or health) adj3 impoverish$).ti,ab. (241)
39 (burden$ adj3 (cost$ or expenditure$ or economic$ or financ$) adj3 (care or health or healthcare or medical$)).ti,ab. (6941)
40 ((willing$ or unwilling$) adj3 pay$ adj3 (care or health or healthcare or medical$)).ti,ab. (458)
41 ((afford$ or unafford$) adj5 (cost$ or pay$)).ti,ab. (3175)
42 ((able or ability or capacit$ or unable or inability) adj2 pay$).ti,ab. (1801)
43 or/23-42 (414755)
44 16 and 43 (5138)
45 22 and 43 (202)
46 44 or 45 (5237)
47 letter.pt. (1118273)
48 editorial.pt. (655452)
49 47 or 48 (1773725)
50 46 not 49 (5179)
51 limit 50 to conference abstracts (970)
52 50 not 51 (4209)
53 limit 52 to yr="2010 -Current" (2958)

Key:

/ = subject heading (EMTREE term)
exp = exploded subject heading (EMTREE term)
$ = truncation
* = truncation
" " = exact phrase search
? = stands for zero or one characters within a word or at the end of a word
ti,ab = terms in either title or abstract fields
mp = multi-purpose fields search – includes title, abstract, author keywords, or subject headings field
kw = terms in author keywords field
sh = terms in the subject heading field
pt = terms in publication type field
adj3 = terms within three words of each other (any order)
adj = terms next to each other in the same order

EconLit
via Ovid http://ovidsp.ovid.com/
1886 to June 11 2020
Search date: 22nd June 2020
Records retrieved: 197

1 (city or cities or megacity or megacities or metropolitan).ti,ab. (35238)
2 (metropolis or megalopolis or municipal$ or conurbation$ or suburb$ or town or towns or township$ or borough$ or barrio or barrios).ti,ab. (15322)
3 ((dense$ or density or high$ or large$ or heavy or heavily) adj3 (populat$ or populous) adj3 (area$ or settlement$ or district$ or neighbourhood$ or communit$)).ti,ab. (356)
4 urban$.ti,ab. (36202)
5 or/1-4 (69848)
6 (afghanistan or albania or algeria or american samoa or angola or "antigua and barbuda" or antigua or barbuda or argentina or armenia or armenian or aruba or azerbaijan or bahrain or bangladesh or barbados or republic of belarus or belarus or byelarus or belorussia or byelorussian or belize or british honduras or benin or dahomey or bhutan or bolivia or "bosnia and herzegovina" or bosnia or herzegovina or botswana or bechuanaland or brazil or brasil or bulgaria or burkina faso or burkina fasso or upper volta or burundi or urundi or cabo verde or cape verde or cambodia or kampuchea or khmer republic or cameroon or cameron or cameroun or central african republic or ubangi shari or chad or chile or china or colombia or comoros or comoro islands or iles comores or mayotte or democratic republic of the congo or democratic republic congo or congo or zaire or costa rica or "cote d'ivoire" or "cote d' ivoire" or cote divoire or cote d ivoire or ivory coast or croatia or cuba or cyprus or czech republic or czechoslovakia or djibouti or french somaliland or dominica or dominican republic or ecuador or egypt or united arab republic or el salvador or equatorial guinea or spanish guinea or eritrea or estonia or eswatini or swaziland or ethiopia or fiji or gabon or gabonese republic or gambia or "georgia (republic)" or georgian or ghana or gold coast or gibraltar or greece or grenada or guam or guatemala or guinea or guinea bissau or guyana or british guiana or haiti or hispaniola or honduras or hungary or india or indonesia or timor or iran or iraq or isle of man or jamaica or jordan or kazakhstan or kazakh or kenya or "democratic people's republic of korea" or republic of korea or north korea or south korea or korea or kosovo or kyrgyzstan or kirghizia or kirgizstan or kyrgyz republic or kirghiz or laos or lao pdr or "lao people's democratic republic" or latvia or lebanon or lebanese republic or lesotho or basutoland or liberia or libya or libyan arab jamahiriya or lithuania or macau or macao or "macedonia (republic)" or macedonia or madagascar or malagasy republic or malawi or nyasaland or malaysia or malay federation or malaya federation or maldives or indian ocean islands or indian ocean or mali or malta or micronesia or federated

For peer review only

states of micronesia or kiribati or marshall islands or nauru or northern mariana islands or palau or tuvalu or mauritania or mauritius or mexico or moldova or moldovian or mongolia or montenegro or morocco or ifni or mozambique or portuguese east africa or myanmar or burma or namibia or nepal or netherlands antilles or nicaragua or niger or nigeria or oman or muscat or pakistan or panama or papua new guinea or new guinea or paraguay or peru or philippines or philipines or phillipines or phillippines or poland or "polish people's republic" or portugal or portuguese republic or puerto rico or romania or russia or russian federation or ussr or soviet union or union of soviet socialist republics or rwanda or ruanda or samoa or pacific islands or polynesia or samoan islands or navigator island or navigator islands or "sao tome and principe" or saudi arabia or senegal or serbia or seychelles or sierra leone or slovakia or slovak republic or slovenia or melanesia or solomon island or solomon islands or norfolk island or norfolk islands or somalia or south africa or south sudan or sri lanka or ceylon or "saint kitts and nevis" or "st. kitts and nevis" or saint lucia or "st. lucia" or "saint vincent and the grenadines" or saint vincent or "st. vincent" or grenadines or sudan or suriname or surinam or dutch guiana or netherlands guiana or syria or syrian arab republic or tajikistan or tadjikistan or tadzhikistan or tadzhik or tanzania or tanganyika or thailand or siam or timor leste or east timor or togo or togolese republic or tonga or "trinidad and tobago" or trinidad or tobago or tunisia or turkey or "turkey (republic)" or turkmenistan or turkmen or uganda or ukraine or uruguay or uzbekistan or uzbek or vanuatu or new hebrides or venezuela or vietnam or viet nam or middle east or west bank or gaza or palestine or yemen or yugoslavia or zambia or zimbabwe or northern rhodesia or global south or africa south of the sahara or sub-saharan africa or subsaharan africa or africa, central or central africa or africa, northern or north africa or northern africa or magreb or maghrib or sahara or africa, southern or southern africa or africa, eastern or east africa or eastern africa or africa, western or west africa or western africa or west indies or indian ocean islands or caribbean or central america or latin america or "south and central america" or south america or asia, central or central asia or asia, northern or north asia or northern asia or asia, southeastern or southeastern asia or south eastern asia or southeast asia or south east asia or asia, western or western asia or europe, eastern or east europe or eastern europe or developing country or developing countries or developing nation? or developing population? or developing world or less developed countr* or less developed nation? or less developed population? or less developed world or lesser developed countr* or lesser developed nation? or lesser developed population? or lesser developed world or under developed countr* or under developed nation? or under developed population? or under developed world or underdeveloped countr* or underdeveloped nation? or underdeveloped population? or underdeveloped world or middle income countr* or middle income nation? or middle income population? or low income countr* or low income nation? or low income population? or lower income countr* or lower income nation? or lower income population? or underserved countr* or underserved nation? or underserved population? or underserved world or under served countr* or under served nation? or under served population? or under served world or deprived countr* or deprived nation? or deprived population? or deprived world or poor countr* or poor nation? or poor population? or poor world or poorer countr* or poorer nation? or poorer population? or poorer world or developing econom* or less developed econom* or lesser developed econom* or under developed econom* or underdeveloped econom* or middle income econom* or low income econom* or lower income econom* or low gdp or low gnp or low gross domestic or low gross national or lower gdp or lower gnp or lower gross domestic or lower gross national or lmic or lmics or third world or lami countr* or transitional countr* or emerging economies or emerging economy or emerging nation?).ti,ab,sh,kw. (270638)

7 (afghan or afghans or afghani or albanian? algerian? or american samoan? or angolan? or antiguan? or barbudan? or argentine? or argentinian? or argentinean? or armenian? or aruban? or azerbaijani? or bahraini? or bangladeshi? or bangalees or bajan? or belarusian? or byelorussian? or belizean? or beninese? or bhutanese or bolivian? or bosnian? or botswana or batswana or brazilian? or brasilian? or bulgarian? or burkinabe or burkinese or burundian? or cape verdean? or cabo verdean? or cambodian? or khmer or cameroonian? or central african? or chadian? or chilean? or

chinese or colombian? or comorian? or congolese or costa rican? or ivorian? or croatian? or cuban? or cypriot? or czech? or djiboutian? or dominican? or ecuadorian? or egyptian? or salvadoran? or equatorial guinean? or equatoguinean? or eritrean? or estonian? or swazi? or swati? or ethiopian? or fijian or gabonese or gabonaise or gambian? or georgian? or ghanaian? or gibraltarian? or greek? or grenadian? or guamanian? or guatemalan? or guinean? or bissau guinean? or guyanese or haitian? or honduran? or hungarian? or indian? or indonesian? or iranian? or iraqian? or iraqi? or manx or jamaican? or jordanian? or kazakhstani? or kenyan? or kirabati or kirabatian? or north korean? or korean? or kosovar? or kosovan? or kyrgyz* or lao or laotian? or latvian? or lebanese or lesothan? or lesothonian? or mosotho or basotho or liberian? or libyan? or lithuanian? or macanese or macedonian? or malagasy or madagascan? or malawian? or malaysian? or maldivian? or malian? or maltese or marshallese? or mauritanian? or mauritian? or mexican? or micronesian? or moldovan? or mongolian? or mongol or montenegrin? or moroccan? or mozambican? or burmese or myanma or namibian? or nauruan? or nepali or nepalese or netherlands antillean? or nicaraguan? or nigerien? or nigerian? or northern mariana islander? or mariana? or omani? or pakistani? or palauan? or panamanian? or papua new guinean? or paraguayan? or peruvian? or philippine? or philipine? or phillipine? or phillippine? or filipino? or filipina? or polish or pole or poles or portuguese or puerto rican? or romanian? or russian? or soviet people or soviet population or rwandan? or rwandese or ruandan? or ruandese or samoan? or sao tomean? or santomean? or saudi arabian? or saudi? or senegalese or serbian? or montenegrin? or seychellois or seychelloise? or sierra leonean? or slovak? or slovene? or solomon islander? or somali? or south african? or south sudanese or sri lankan? or ceylonese or kittitian? or nevisian? or saint lucian? or vincentian? or sudanese or surinamese? or syrian? or tajik? or tajikistani? or tanzanian? or tanganyikan? or thai or timorese? or togolese or tongan? or trinidadian? or tobagonian? or tunisian? or turk? or turkish or turkmen? or tuvaluan? or ugandan? or ukrainian? or uruguayan? or uzbek? or vanuatu* or venezuelan? or vietnamese or yemeni? or yemenite? or yemenese or yugoslav? or yugoslavian? or zambian? or zimbabwean?).ti,ab,sh,kw. (120512)
8 6 or 7 (320416)
9 5 and 8 (23130)
10 (slum or slums or ghetto or ghettos or shanty$ or shanties or shack$ or favela$).ti,ab. (1156)
11 (informal$ adj3 settlement$).ti,ab. (206)
12 ((poverty or impoverish$) adj3 (area$ or settlement$)).ti,ab. (379)
13 ((precarious$ or irregular$) adj3 settlement$).ti,ab. (11)
14 (squatter$ adj3 (area$ or settlement$)).ti,ab. (70)
15 or/10-14 (1743)
16 (cost$ adj2 (illness$ or sickness$ or disease$)).ti,ab. (415)
17 (cost$ adj3 (care or health or healthcare or medical$)).ti,ab. (3305)
18 (expenditure$ adj3 (care or health or healthcare or medical$)).ti,ab. (2943)
19 ((direct or indirect) adj2 (cost$ or expenditure$)).ti,ab. (1415)
20 out of pocket.ti,ab. (868)
21 (OOP adj3 (cost$ or expense$ or expenditure$ or financ$ or pay$ or paid or spend$)).ti,ab. (71)
22 OOPE.ti,ab. (4)
23 ((personal or individual$ or patient$ or family$ or families or household$) adj3 (budget$ or cost$ or expense$ or expenditure$ or financ$ or pay$ or paid or spend$) adj3 (care or health or healthcare or medical$)).ti,ab. (599)
24 (catastroph$ adj10 (cost$ or expense$ or expenditure$ or financ$ or pay$ or paid or spend$)).ti,ab. (449)
25 ((medical$ or health) adj3 impoverish$).ti,ab. (23)
26 (burden$ adj3 (cost$ or expenditure$ or economic$ or financ$) adj3 (care or health or healthcare or medical$)).ti,ab. (106)
27 ((willing$ or unwilling$) adj3 pay$ adj3 (care or health or healthcare or medical$)).ti,ab. (127)
28 ((afford$ or unafford$) adj5 (cost$ or pay$)).ti,ab. (315)

29 ((able or ability or capacit$ or unable or inability) adj2 pay$).ti,ab. (717)
30 or/16-29 (9428)
31 9 and 30 (292)
32 15 and 30 (18)
33 31 or 32 (302)
34 limit 33 to yr="2010 -Current" (197)

Key:
$ = truncation
* = truncation
" " = exact phrase search
? = stands for zero or one characters within a word or at the end of a word
ti,ab = terms in either title or abstract fields
kw = terms in keywords field
sh = terms in the subject heading field
adj3 = terms within three words of each other (any order)
adj = terms next to each other in the same order

Science Citation Index
via Web of Science, Clarivate Analytics https://clarivate.com/
1900 – 19th June 2020
Searched on: 22nd June 2020
Records retrieved: 1538

# 35 1,538 #32 not #33
Indexes=SCI-EXPANDED Timespan=2010-2020
# 34 1,922 #32 not #33
# 33 1,028,173 CF=conference
# 32 1,929 #31 OR #30
# 31 99 #29 AND #14
# 30 1,888 #29 AND #8
# 29 102,229 #28 OR #27 OR #26 OR #25 OR #24 OR #23 OR #22 OR #21 OR #20 OR #19 OR #18 OR #17 OR #16 OR #15
# 28 1,622 TS=((able or ability or capacit* or unable or inability) NEAR/2 pay*)
# 27 3,128 TS=((afford* or unafford*) NEAR/5 (cost* or pay*))
# 26 379 TS=((willing* or unwilling*) NEAR/3 pay* NEAR/3 (care or health or healthcare or medical*))
# 25 4,603 TS=(burden* NEAR/3 (cost* or expenditure* or economic* or financ*) NEAR/3 (care or health or healthcare or medical*))
# 24 207 TS=((medical* or health) NEAR/3 impoverish*)
# 23 1,220 TS=(catastroph* NEAR/10 (cost* or expense* or expenditure* or financ* or pay* or paid or spend*))
# 22 11,648 TS=((personal or individual* or patient* or family* or families or household*) NEAR/3 (budget* or cost* or expense* or expenditure* or financ* or pay* or paid or spend*) NEAR/3 (care or health or healthcare or medical*))
# 21 46 TS=OOPE
# 20 368 TS=(OOP NEAR/3 (cost* or expense* or expenditure* or financ* or pay* or paid or spend*))
# 19 4,311 TS="out of pocket"
# 18 16,894 TS=((direct or indirect) NEAR/2 (cost* or expenditure*))
# 17 11,186 TS=(expenditure* NEAR/3 (care or health or healthcare or medical*))

For peer review only

16 68,145 TS=(cost* NEAR/3 (care or health or healthcare or medical*))
15 8,010 TS=(cost* NEAR/2 (illness* or sickness* or disease*))
14 7,749 #13 OR #12 OR #11 OR #10 OR #9
13 99 TS=(squatter* NEAR/3 (area* or settlement*))
12 37 TS=((precarious* or irregular*) NEAR/3 settlement*)
11 1,094 TS=((poverty or impoverish*) NEAR/3 (area* or settlement*))
10 791 TS=(informal NEAR/3 settlement*)
9 6,013 TS=(slum or slums or ghetto or ghettos or shanty* or shanties or shack* or favela*)
8 186,228 #7 AND #4
7 2,792,724 #6 OR #5
6 589,412 TS=(afghan or afghans or afghani or albanian? algerian? or american samoan? or angolan? or antiguan? or barbudan? or argentine? or argentinian? or argentinean? or armenian? or aruban? or azerbaijani? or bahraini? or bangladeshi? or bangalees or bajan? or belarusian? or byelorussian? or belizean? or beninese? or bhutanese or bolivian? or bosnian? or botswana or batswana or brazilian? or brasilian? or bulgarian? or burkinabe or burkinese or burundian? or cape verdean? or cabo verdean? or cambodian? or khmer or cameroonian? or central african? or chadian? or chilean? or chinese or colombian? or comorian? or congolese or costa rican? or ivorian? or croatian? or cuban? or cypriot? or czech? or djiboutian? or dominican? or ecuadorian? or egyptian? or salvadoran? or equatorial guinean? or equatoguinean? or eritrean? or estonian? or swazi? or swati? or ethiopian? or fijian or gabonese or gabonaise or gambian? or georgian? or ghanaian? or gibraltarian? or greek? or grenadian? or guamanian? or guatemalan? or guinean? or bissau guinean? or guyanese or haitian? or honduran? or hungarian? or indian? or indonesian? or iranian? or iraqian? or iraqi? or manx or jamaican? or jordanian? or kazakhstani? or kenyan? or kirabati or kirabatian? or north korean? or korean? or kosovar? or kosovan? or kyrgyz* or lao or laotian? or latvian? or lebanese or lesothan? or lesothonian? or mosotho or basotho or liberian? or libyan? or lithuanian? or macanese or macedonian? or malagasy or madagascan? or malawian? or malaysian? or maldivian? or malian? or maltese or marshallese? or mauritanian? or mauritian? or mexican? or micronesian? or moldovan? or mongolian? or mongol or montenegrin? or moroccan? or mozambican? or burmese or myanma or namibian? or nauruan? or nepali or nepalese or netherlands antillean? or nicaraguan? or nigerien? or nigerian? or northern mariana islander? or mariana? or omani? or pakistani? or palauan? or panamanian? or papua new guinean? or paraguayan? or peruvian? or philippine? or philipine? or phillipine? or phillippine? or filipino? or filipina? or polish or pole or poles or portuguese or puerto rican? or romanian? or russian? or soviet people or soviet population or rwandan? or rwandese or ruandan? or ruandese or samoan? or sao tomean? or santomean? or saudi arabian? or saudi? or senegalese or serbian? or montenegrin? or seychellois or seychelloise? or sierra leonean? or slovak? or slovene? or solomon islander? or somali? or south african? or south sudanese or sri lankan? or ceylonese or kittitian? or nevisian? or saint lucian? or vincentian? or sudanese or surinamese? or syrian? or tajik? or tajikistani? or tanzanian? or tanganyikan? or thai or timorese? or togolese or tongan? or trinidadian? or tobagonian? or tunisian? or turk? or turkish or turkmen? or tuvaluan? or ugandan? or ukrainian? or uruguayan? or uzbek? or vanuatu* or venezuelan? or vietnamese or yemeni? or yemenite? or yemenese or yugoslav? or yugoslavian? or zambian? or zimbabwean?)
5 2,363,830 TS=(afghanistan or albania or algeria or "american samoa" or angola or "antigua and barbuda" or antigua or barbuda or argentina or armenia or armenian or aruba or azerbaijan or bahrain or bangladesh or barbados or "republic of belarus" or belarus or byelarus or belorussia or byelorussian or belize or "british honduras" or benin or dahomey or bhutan or bolivia or "bosnia and herzegovina" or bosnia or herzegovina or botswana or

bechuanaland or brazil or brasil or bulgaria or "burkina faso" or "burkina fasso" or "upper volta" or burundi or urundi or "cabo verde" or "cape verde" or cambodia or kampuchea or "khmer republic" or cameroon or cameron or cameroun or "central african republic" or "ubangi shari" or chad or chile or china or colombia or comoros or "comoro islands" or "iles comores" or mayotte or "democratic republic of the congo" or "democratic republic congo" or congo or zaire or "costa rica" or "cote d’ivoire" or "cote d’ ivoire" or "cote divoire" or "cote d ivoire" or "ivory coast" or croatia or cuba or cyprus or "czech republic" or czechoslovakia or djibouti or "french somaliland" or dominica or "dominican republic" or ecuador or egypt or "united arab republic" or "el salvador" or "equatorial guinea" or "spanish guinea" or eritrea or estonia or eswatini or swaziland or ethiopia or fiji or gabon or "gabonese republic" or gambia or "georgia (republic) " or georgian or ghana or "gold coast" or gibraltar or greece or grenada or guam or guatemala or guinea or "guinea bissau" or guyana or "british guiana" or haiti or hispaniola or honduras or hungary or india or indonesia or timor or iran or iraq or "isle of man" or jamaica or jordan or kazakhstan or kazakh or kenya or "democratic people’s republic of korea" or "republic of korea" or "north korea" or "south korea" or korea or kosovo or kyrgyzstan or kirghizia or kirgizstan or "kyrgyz republic" or kirghiz or laos or "lao pdr" or "lao people's democratic republic" or latvia or lebanon or "lebanese republic" or lesotho or basutoland or liberia or libya or "libyan arab jamahiriya" or lithuania or macau or macao or "macedonia (republic) " or macedonia or madagascar or "malagasy republic" or malawi or nyasaland or malaysia or "malay federation" or "malaya federation" or maldives or "indian ocean islands" or "indian ocean" or mali or malta or micronesia or "federated states of micronesia" or kiribati or "marshall islands" or nauru or "northern mariana islands" or palau or tuvalu or mauritania or mauritius or mexico or moldova or moldovian or mongolia or montenegro or morocco or ifni or mozambique or "portuguese east africa" or myanmar or burma or namibia or nepal or "netherlands antilles" or nicaragua or niger or nigeria or oman or muscat or pakistan or panama or "papua new guinea" or "new guinea" or paraguay or peru or philippines or philipines or phillipines or phillippines or poland or "polish people's republic" or portugal or "portuguese republic" or "puerto rico" or romania or russia or "russian federation" or ussr or "soviet union" or "union of soviet socialist republics" or rwanda or ruanda or samoa or "pacific islands" or polynesia or "samoan islands" or "navigator island" or "navigator islands" or "sao tome and principe" or "saudi arabia" or senegal or serbia or seychelles or "sierra leon"e or slovakia or "slovak republic" or slovenia or melanesia or "solomon island" or "solomon islands" or "norfolk island" or "norfolk islands" or somalia or "south africa" or "south sudan" or "sri lanka" or ceylon or "saint kitts and nevis" or "st. kitts and nevis" or "saint lucia" or "st. lucia" or "saint vincent and the grenadines" or "saint vincent" or "st. vincent" or grenadines or sudan or suriname or surinam or "dutch guiana" or "netherlands guiana" or syria or "syrian arab republic" or tajikistan or tadjikistan or tadzhikistan or tadzhik or tanzania or tanganyika or thailand or siam or "timor leste" or "east timor" or togo or "togolese republic" or tonga or "trinidad and tobago" or trinidad or tobago or tunisia or turkey or "turkey (republic) " or turkmenistan or turkmen or uganda or ukraine or uruguay or uzbekistan or uzbek or vanuatu or "new hebrides" or venezuela or vietnam or "viet nam" or "middle east" or "west bank" or gaza or palestine or yemen or yugoslavia or zambia or zimbabwe or "northern rhodesia" or "global south" or "africa south of the sahara" or "sub-saharan africa" or "subsaharan africa" or "africa, central" or "central africa" or "africa, northern" or "north africa" or "northern africa" or magreb or maghrib or sahara or "africa, southern" or "southern africa" or "africa, eastern" or "east africa" or "eastern africa" or "africa, western" or "west africa" or "western africa" or "west indies" or "indian ocean islands" or caribbean or "central america" or "latin america" or "south and central america" or "south america" or "asia, central" or "central asia" or "asia, northern" or "north asia" or "northern asia" or "asia, southeastern" or "southeastern asia" or "south eastern asia" or "southeast asia" or "south east asia" or "asia,

For peer review only

western" or "western asia" or "europe, eastern" or "east europe" or "eastern europe" or "developing country" or "developing countries" or "developing nation" or "developing nations" or "developing population" or "developing populations" or "developing world" or "less developed country" or "less developed countries" or "less developed nation" or "less developed nations" or "less developed population" or "less developed populations" or "less developed world" or "lesser developed country" or "lesser developed countries" or "lesser developed nation" or "lesser developed nations" or "lesser developed population" or "lesser developed populations" or "lesser developed world" or "under developed country" or "under developed countries" or "under developed nation" or "under developed nations" or "under developed population" or "under developed populations" or "under developed world" or "underdeveloped country" or "underdeveloped countries" or "underdeveloped nation" or "underdeveloped nations" or "underdeveloped population" or "underdeveloped populations" or "underdeveloped world" or "middle income country" or "middle income countries" or "middle income nation" or "middle income nations" or "middle income population" or "middle income populations" or "low income country" or "low income countries" or "low income nation" or "low income nations" or "low income population" or "low income populations" or "lower income country" or "lower income countries" or "lower income nation" or "lower income nations" or "lower income population" or "lower income populations" or "underserved country" or "underserved countries" or "underserved nation" or "underserved nations" or "underserved population" or "underserved populations" or "underserved world" or "under served country" or "under served countries" or "under served nation" or "under served nations" or "under served population" or "under served populations" or "under served world" or "deprived country" or "deprived countries" or "deprived nation" or "deprived nations" or "deprived population" or "deprived populations" or "deprived world" or "poor country" or "poor countries" or "poor nation" or "poor nations" or "poor population" or "poor populations" or "poor world" or "poorer country" or "poorer countries" or "poorer nation" or "poorer nations" or "poorer population" or "poorer populations" or "poorer world" or "developing economy" or "developing economies" or "less developed economy" or "less developed economies" or "lesser developed economy" or "lesser developed economies" or "under developed economy" or "under developed economies" or "underdeveloped economy" or "underdeveloped economies" or "middle income economy" or "middle income economies" or "low income economy" or "low income economies" or "lower income economy" or "lower income economies" or "low gdp" or "low gnp" or "low gross domestic" or "low gross national" or "lower gdp" or "lower gnp" or "lower gross domestic" or "lower gross national" or lmic or lmics or "third world" or "lami country" or "lami countries" or "transitional country" or "transitional countries" or "emerging economies" or "emerging economy" or "emerging nation" or "emerging nations")

4 517,582 #3 OR #2 OR #1

3 7,570 TS=((dense* or density or high* or large* or heavy or heavily) NEAR/3 (populat* or populous) NEAR/3 (area* or settlement* or district* or neighbourhood* or communit*))

2 121,532 TS=(metropolis or megalopolis or municipal* or conurbation* or suburb* or town or towns or township* or borough* or barrio or barrios)

1 422,185 TS=(urban* or city or cities or megacity or megacities or metropolitan)

Key:

* = truncation

" " = exact phrase search

? = stands for zero or one character

TS = topic search - terms in title, abstract, keyword fields

NEAR/3 = terms within three words of each other (any order)

For peer review only

Social Science Citation Index
via Web of Science, Clarivate Analytics https://clarivate.com/
1956 – 19th June 2020
Searched on: 22nd June 2020
Records retrieved: 1226

# 35 1,226 #32 not #33
Indexes=SSCI Timespan=2010-2020
# 34 1,545 #32 not #33
# 33 58,510 CF=conference
# 32 1,557 #31 OR #30
# 31 87 #29 AND #14
# 30 1,527 #29 AND #8
# 29 50,953 #28 OR #27 OR #26 OR #25 OR #24 OR #23 OR #22 OR #21 OR #20 OR #19 OR #18 OR #17 OR #16 OR #15
# 28 1,469 TS=((able or ability or capacit* or unable or inability) NEAR/2 pay*)
# 27 1,102 TS=((afford* or unafford*) NEAR/5 (cost* or pay*))
# 26 364 TS=((willing* or unwilling*) NEAR/3 pay* NEAR/3 (care or health or healthcare or medical*))
# 25 1,709 TS=(burden* NEAR/3 (cost* or expenditure* or economic* or financ*) NEAR/3 (care or health or healthcare or medical*))
# 24 235 TS=((medical* or health) NEAR/3 impoverish*)
# 23 1,100 TS=(catastroph* NEAR/10 (cost* or expense* or expenditure* or financ* or pay* or paid or spend*))
# 22 6,230 TS=((personal or individual* or patient* or family* or families or household*) NEAR/3 (budget* or cost* or expense* or expenditure* or financ* or pay* or paid or spend*) NEAR/3 (care or health or healthcare or medical*))
# 21 41 TS=OOPE
# 20 358 TS=(OOP NEAR/3 (cost* or expense* or expenditure* or financ* or pay* or paid or spend*))
# 19 3,675 TS="out of pocket"
# 18 6,153 TS=((direct or indirect) NEAR/2 (cost* or expenditure*))
# 17 9,050 TS=(expenditure* NEAR/3 (care or health or healthcare or medical*))
# 16 32,024 TS=(cost* NEAR/3 (care or health or healthcare or medical*))
# 15 3,308 TS=(cost* NEAR/2 (illness* or sickness* or disease*))
# 14 8,916 #13 OR #12 OR #11 OR #10 OR #9
# 13 357 TS=(squatter* NEAR/3 (area* or settlement*))
# 12 44 TS=((precarious* or irregular*) NEAR/3 settlement*)
# 11 1,297 TS=((poverty or impoverish*) NEAR/3 (area* or settlement*))
# 10 1,304 TS=(informal NEAR/3 settlement*)
# 9 6,468 TS=(slum or slums or ghetto or ghettos or shanty* or shanties or shack* or favela*)
# 8 103,299 #7 AND #4
# 7 984,954 #6 OR #5
# 6 197,784 TS=(afghan or afghans or afghani or albanian? algerian? or american samoan? or angolan? or antiguan? or barbudan? or argentine? or argentinian? or argentinean? or armenian? or aruban? or azerbaijani? or bahraini? or bangladeshi? or bangalees or bajan? or belarusian? or byelorussian? or belizean? or beninese? or bhutanese or bolivian? or bosnian? or botswana or batswana or brazilian? or brasilian? or bulgarian? or burkinabe or burkinese or burundian? or cape verdean? or cabo verdean? or cambodian? or

For peer review only

khmer or cameroonian? or central african? or chadian? or chilean? or chinese or colombian? or comorian? or congolese or costa rican? or ivorian? or croatian? or cuban? or cypriot? or czech? or djiboutian? or dominican? or ecuadorian? or egyptian? or salvadoran? or equatorial guinean? or equatoguinean? or eritrean? or estonian? or swazi? or swati? or ethiopian? or fijian or gabonese or gabonaise or gambian? or georgian? or ghanaian? or gibraltarian? or greek? or grenadian? or guamanian? or guatemalan? or guinean? or bissau guinean? or guyanese or haitian? or honduran? or hungarian? or indian? or indonesian? or iranian? or iraqian? or iraqi? or manx or jamaican? or jordanian? or kazakhstani? or kenyan? or kirabati or kirabatian? or north korean? or korean? or kosovar? or kosovan? or kyrgyz* or lao or laotian? or latvian? or lebanese or lesothan? or lesothonian? or mosotho or basotho or liberian? or libyan? or lithuanian? or macanese or macedonian? or malagasy or madagascan? or malawian? or malaysian? or maldivian? or malian? or maltese or marshallese? or mauritanian? or mauritian? or mexican? or micronesian? or moldovan? or mongolian? or mongol or montenegrin? or moroccan? or mozambican? or burmese or myanma or namibian? or nauruan? or nepali or nepalese or netherlands antillean? or nicaraguan? or nigerien? or nigerian? or northern mariana islander? or mariana? or omani? or pakistani? or palauan? or panamanian? or papua new guinean? or paraguayan? or peruvian? or philippine? or philipine? or phillipine? or phillippine? or filipino? or filipina? or polish or pole or poles or portuguese or puerto rican? or romanian? or russian? or soviet people or soviet population or rwandan? or rwandese or ruandan? or ruandese or samoan? or sao tomean? or santomean? or saudi arabian? or saudi? or senegalese or serbian? or montenegrin? or seychellois or seychelloise? or sierra leonean? or slovak? or slovene? or solomon islander? or somali? or south african? or south sudanese or sri lankan? or ceylonese or kittitian? or nevisian? or saint lucian? or vincentian? or sudanese or surinamese? or syrian? or tajik? or tajikistani? or tanzanian? or tanganyikan? or thai or timorese? or togolese or tongan? or trinidadian? or tobagonian? or tunisian? or turk? or turkish or turkmen? or tuvaluan? or ugandan? or ukrainian? or uruguayan? or uzbek? or vanuatu* or venezuelan? or vietnamese or yemeni? or yemenite? or yemenese or yugoslav? or yugoslavian? or zambian? or zimbabwean?)

5 882,717 TS=(afghanistan or albania or algeria or "american samoa" or angola or "antigua and barbuda" or antigua or barbuda or argentina or armenia or armenian or aruba or azerbaijan or bahrain or bangladesh or barbados or "republic of belarus" or belarus or byelarus or belorussia or byelorussian or belize or "british honduras" or benin or dahomey or bhutan or bolivia or "bosnia and herzegovina" or bosnia or herzegovina or botswana or bechuanaland or brazil or brasil or bulgaria or "burkina faso" or "burkina fasso" or "upper volta" or burundi or urundi or "cabo verde" or "cape verde" or cambodia or kampuchea or "khmer republic" or cameroon or cameron or cameroun or "central african republic" or "ubangi shari" or chad or chile or china or colombia or comoros or "comoro islands" or "iles comores" or mayotte or "democratic republic of the congo" or "democratic republic congo" or congo or zaire or "costa rica" or "cote d’ivoire" or "cote d’ ivoire" or "cote divoire" or "cote d ivoire" or "ivory coast" or croatia or cuba or cyprus or "czech republic" or czechoslovakia or djibouti or "french somaliland" or dominica or "dominican republic" or ecuador or egypt or "united arab republic" or "el salvador" or "equatorial guinea" or "spanish guinea" or eritrea or estonia or eswatini or swaziland or ethiopia or fiji or gabon or "gabonese republic" or gambia or "georgia (republic) " or georgian or ghana or "gold coast" or gibraltar or greece or grenada or guam or guatemala or guinea or "guinea bissau" or guyana or "british guiana" or haiti or hispaniola or honduras or hungary or india or indonesia or timor or iran or iraq or "isle of man" or jamaica or jordan or kazakhstan or kazakh or kenya or "democratic people’s republic of korea" or "republic of korea" or "north korea" or "south korea" or korea or kosovo or kyrgyzstan or kirghizia or kirgizstan or "kyrgyz republic" or kirghiz or laos or "lao pdr" or "lao people's democratic republic" or latvia or lebanon or

For peer review only

"lebanese republic" or lesotho or basutoland or liberia or libya or "libyan arab jamahiriya" or lithuania or macau or macao or "macedonia (republic) " or macedonia or madagascar or "malagasy republic" or malawi or nyasaland or malaysia or "malay federation" or "malaya federation" or maldives or "indian ocean islands" or "indian ocean" or mali or malta or micronesia or "federated states of micronesia" or kiribati or "marshall islands" or nauru or "northern mariana islands" or palau or tuvalu or mauritania or mauritius or mexico or moldova or moldovian or mongolia or montenegro or morocco or ifni or mozambique or "portuguese east africa" or myanmar or burma or namibia or nepal or "netherlands antilles" or nicaragua or niger or nigeria or oman or muscat or pakistan or panama or "papua new guinea" or "new guinea" or paraguay or peru or philippines or philipines or phillipines or phillippines or poland or "polish people's republic" or portugal or "portuguese republic" or "puerto rico" or romania or russia or "russian federation" or ussr or "soviet union" or "union of soviet socialist republics" or rwanda or ruanda or samoa or "pacific islands" or polynesia or "samoan islands" or "navigator island" or "navigator islands" or "sao tome and principe" or "saudi arabia" or senegal or serbia or seychelles or "sierra leon"e or slovakia or "slovak republic" or slovenia or melanesia or "solomon island" or "solomon islands" or "norfolk island" or "norfolk islands" or somalia or "south africa" or "south sudan" or "sri lanka" or ceylon or "saint kitts and nevis" or "st. kitts and nevis" or "saint lucia" or "st. lucia" or "saint vincent and the grenadines" or "saint vincent" or "st. vincent" or grenadines or sudan or suriname or surinam or "dutch guiana" or "netherlands guiana" or syria or "syrian arab republic" or tajikistan or tadjikistan or tadzhikistan or tadzhik or tanzania or tanganyika or thailand or siam or "timor leste" or "east timor" or togo or "togolese republic" or tonga or "trinidad and tobago" or trinidad or tobago or tunisia or turkey or "turkey (republic) " or turkmenistan or turkmen or uganda or ukraine or uruguay or uzbekistan or uzbek or vanuatu or "new hebrides" or venezuela or vietnam or "viet nam" or "middle east" or "west bank" or gaza or palestine or yemen or yugoslavia or zambia or zimbabwe or "northern rhodesia" or "global south" or "africa south of the sahara" or "sub-saharan africa" or "subsaharan africa" or "africa, central" or "central africa" or "africa, northern" or "north africa" or "northern africa" or magreb or maghrib or sahara or "africa, southern" or "southern africa" or "africa, eastern" or "east africa" or "eastern africa" or "africa, western" or "west africa" or "western africa" or "west indies" or "indian ocean islands" or caribbean or "central america" or "latin america" or "south and central america" or "south america" or "asia, central" or "central asia" or "asia, northern" or "north asia" or "northern asia" or "asia, southeastern" or "southeastern asia" or "south eastern asia" or "southeast asia" or "south east asia" or "asia, western" or "western asia" or "europe, eastern" or "east europe" or "eastern europe" or "developing country" or "developing countries" or "developing nation" or "developing nations" or "developing population" or "developing populations" or "developing world" or "less developed country" or "less developed countries" or "less developed nation" or "less developed nations" or "less developed population" or "less developed populations" or "less developed world" or "lesser developed country" or "lesser developed countries" or "lesser developed nation" or "lesser developed nations" or "lesser developed population" or "lesser developed populations" or "lesser developed world" or "under developed country" or "under developed countries" or "under developed nation" or "under developed nations" or "under developed population" or "under developed populations" or "under developed world" or "underdeveloped country" or "underdeveloped countries" or "underdeveloped nation" or "underdeveloped nations" or "underdeveloped population" or "underdeveloped populations" or "underdeveloped world" or "middle income country" or "middle income countries" or "middle income nation" or "middle income nations" or "middle income population" or "middle income populations" or "low income country" or "low income countries" or "low income nation" or "low income nations" or "low income population" or "low income populations" or "lower income country" or "lower income countries" or "lower income nation" or "lower income

For peer review only

nations" or "lower income population" or "lower income populations" or "underserved country" or "underserved countries" or "underserved nation" or "underserved nations" or "underserved population" or "underserved populations" or "underserved world" or "under served country" or "under served countries" or "under served nation" or "under served nations" or "under served population" or " under served populations" or "under served world" or "deprived country" or "deprived countries" or "deprived nation" or "deprived nations" or "deprived population" or "deprived populations" or "deprived world" or "poor country" or "poor countries" or "poor nation" or "poor nations" or "poor population" or "poor populations" or "poor world" or "poorer country" or "poorer countries" or "poorer nation" or "poorer nations" or "poorer population" or "poorer populations" or "poorer world" or "developing economy" or "developing economies" or "less developed economy" or "less developed economies" or "lesser developed economy" or "lesser developed economies" or "under developed economy" or "under developed economies" or "underdeveloped economy" or "underdeveloped economies" or "middle income economy" or "middle income economies" or "low income economy" or "low income economies" or "lower income economy" or "lower income economies" or "low gdp" or "low gnp" or "low gross domestic" or "low gross national" or "lower gdp" or "lower gnp" or "lower gross domestic" or "lower gross national" or lmic or lmics or "third world" or "lami country" or "lami countries" or "transitional country" or "transitional countries" or "emerging economies" or "emerging economy" or "emerging nation" or "emerging nations")

4 354,340 #3 OR #2 OR #1

3 2,339 TS=((dense* or density or high* or large* or heavy or heavily) NEAR/3 (populat* or populous) NEAR/3 (area* or settlement* or district* or neighbourhood* or communit*))

2 70,206 TS=(metropolis or megalopolis or municipal* or conurbation* or suburb* or town or towns or township* or borough* or barrio or barrios)

1 309,493 TS=(urban* or city or cities or megacity or megacities or metropolitan)

Key:
* = truncation
" " = exact phrase search
? = stands for zero or one character
TS = topic search - terms in title, abstract, keyword fields
NEAR/3 = terms within three words of each other (any order)

Global Index Medicus
https://www.globalindexmedicus.net/
Searched on: 23rd June 2020
Records retrieved: 1274

6 separate searches were carried out and results deduplicated in EndNote.

1. (tw:(urban* OR city OR cities OR megacity OR megacities OR metropolitan OR metropolis OR megalopolis OR municipal* OR conurbation* OR suburb* OR town OR towns OR township* OR borough* OR barrio OR barrios OR "Urban Population" OR "Urban Health Services" OR "Hospitals, Municiple" OR "Urban Health" OR "Hospitals, Urban")) AND (ti:(cost or costs or expenditure* or expense* or spend* or pay* or paid or budget* or financ* or economic*)) AND (tw:(care OR healthcare OR health OR medical*))
2010-2020
607 hits

For peer review only

2. (tw:(urban* OR city OR cities OR megacity OR megacities OR metropolitan OR metropolis OR megalopolis OR municipal* OR conurbation* OR suburb* OR town OR towns OR township* OR borough* OR barrio OR barrios OR "Urban Population" OR "Urban Health Services" OR "Hospitals, Municiple" OR "Urban Health" OR "Hospitals, Urban")) AND (tw:("Health Care Costs" OR "Health Expenditures" OR "Cost of Illness" OR "Financing Personal" OR "Catastrophic Expenditure" OR "Catastrophic Illness/EC" OR catastroph* OR "out of pocket" OR oop OR oope OR "willingness to pay"))
2010-2020
530 hits

3. (tw:(slum OR slums OR ghetto OR ghettos OR shanty* OR shanties OR shack* OR favela* OR squat* OR "informal settlement" OR "informal settlements" OR "Poverty Areas")) AND (tw:(cost or costs or expenditure* or expense* or spend* or pay* or paid or budget* or financ* or economic*)) AND (tw:(care OR health OR healthcare OR medical*))
2010-2020
189 hits

4. tw:(slum OR slums OR ghetto OR ghettos OR shanty* OR shanties OR shack* OR favela* OR squat* OR "informal settlement" OR "informal settlements" OR "Poverty Areas")) AND (tw:("Health Care Costs" OR "Health Expenditures" OR "Cost of Illness" OR "Financing Personal" OR "Catastrophic Expenditure" OR "Catastrophic Illness/EC" OR catastroph* OR "out of pocket" OR oop OR oope OR "willingness to pay"))
2010-2020
10 hits

5. (tw:(urban* OR city OR cities OR megacity OR megacities OR metropolitan OR metropolis OR megalopolis OR municipal* OR conurbation* OR suburb* OR town OR towns OR township* OR borough* OR barrio OR barrios OR "Urban Population" OR "Urban Health Services" OR "Hospitals, Municiple" OR "Urban Health" OR "Hospitals, Urban")) AND (tw:(impoverish*)) AND (tw:(health OR medical*))
2010-2020
188 hits

6. (tw:(slum OR slums OR ghetto OR ghettos OR shanty* OR shanties OR shack* OR favela* OR squat* OR "informal settlement" OR "informal settlements" OR "Poverty Areas")) AND (tw:(impoverish*)) AND (tw:(health OR medical*))
2010-2020
24 hits

Key:
tw = terms in the title, abstract or subject heading fields
* = truncation
" "   = exact phrase

ProQuest Dissertations & Theses A&I
via ProQuest https://www.proquest.com/
Searched on: 23rd June 2020
Records retrieved: 283

10 separate searches were carried out and results deduplicated in EndNote. The following search line was added to each search to limit retrieval to low- middle-income countries:

For peer review only

(TI,AB,SU(afghanistan OR albania OR algeria OR "american samoa" OR angola OR "antigua and barbuda" OR antigua OR barbuda OR argentina OR armenia OR armenian OR aruba OR azerbaijan OR bahrain OR bangladesh OR barbados OR "republic of belarus" OR belarus OR byelarus OR belorussia OR byelorussian OR belize OR "british honduras" OR benin OR dahomey OR bhutan OR bolivia OR "bosnia and herzegovina" OR bosnia OR herzegovina OR botswana OR bechuanaland OR brazil OR brasil OR bulgaria OR "burkina faso" OR "burkina fasso" OR "upper volta" OR burundi OR urundi OR "cabo verde" OR "cape verde" OR cambodia OR kampuchea OR "khmer republic" OR cameroon OR cameron OR cameroun OR "central african republic" OR "ubangi shari" OR chad OR chile OR china OR colombia OR comoros OR "comoro islands" OR "iles comores" OR mayotte OR "democratic republic of the congo" OR "democratic republic congo" OR congo OR zaire OR "costa rica" OR "cote d’ivoire" OR "cote d’ ivoire" OR "cote divoire" OR "cote d ivoire" OR "ivory coast" OR croatia OR cuba OR cyprus OR "czech republic" OR czechoslovakia OR djibouti OR "french somaliland" OR dominica OR "dominican republic" OR ecuador OR egypt OR "united arab republic" OR "el salvador" OR "equatorial guinea" OR "spanish guinea" OR eritrea OR estonia OR eswatini OR swaziland OR ethiopia OR fiji OR gabon OR "gabonese republic" OR gambia OR "georgia (republic) " OR georgian OR ghana OR "gold coast" OR gibraltar OR greece OR grenada OR guam OR guatemala OR guinea OR "guinea bissau" OR guyana OR "british guiana" OR haiti OR hispaniola OR honduras OR hungary OR india OR indonesia OR timor OR iran OR iraq OR "isle of man" OR jamaica OR jordan OR kazakhstan OR kazakh OR kenya OR "democratic people’s republic of korea" OR "republic of korea" OR "north korea" OR "south korea" OR korea OR kosovo OR kyrgyzstan OR kirghizia OR kirgizstan OR "kyrgyz republic" OR kirghiz OR laos OR "lao pdr" OR "lao people's democratic republic" OR latvia OR lebanon OR "lebanese republic" OR lesotho OR basutoland OR liberia OR libya OR "libyan arab jamahiriya" OR lithuania OR macau OR macao OR "macedonia (republic) " OR macedonia OR madagascar OR "malagasy republic" OR malawi OR nyasaland OR malaysia OR "malay federation" OR "malaya federation" OR maldives OR "indian ocean islands" OR "indian ocean" OR mali OR malta OR micronesia OR "federated states of micronesia" OR kiribati OR "marshall islands" OR nauru OR "northern mariana islands" OR palau OR tuvalu OR mauritania OR mauritius OR mexico OR moldova OR moldovian OR mongolia OR montenegro OR morocco OR ifni OR mozambique OR "portuguese east africa" OR myanmar OR burma OR namibia OR nepal OR "netherlands antilles" OR nicaragua OR niger OR nigeria OR oman OR muscat OR pakistan OR panama OR "papua new guinea" OR "new guinea" OR paraguay OR peru OR philippines OR philipines OR phillipines OR phillippines OR poland OR "polish people's republic" OR portugal OR "portuguese republic" OR "puerto rico" OR romania OR russia OR "russian federation" OR ussr OR "soviet union" OR "union of soviet socialist republics" OR rwanda OR ruanda OR samoa OR "pacific islands" OR polynesia OR "samoan islands" OR "navigator island" OR "navigator islands" OR "sao tome and principe" OR "saudi arabia" OR senegal OR serbia OR seychelles OR "sierra leon" e OR slovakia OR "slovak republic" OR slovenia OR melanesia OR "solomon island" OR "solomon islands" OR "norfolk island" OR "norfolk islands" OR somalia OR "south africa" OR "south sudan" OR "sri lanka" OR ceylon OR "saint kitts and nevis" OR "st. kitts and nevis" OR "saint lucia" OR "st. lucia" OR "saint vincent and the grenadines" OR "saint vincent" OR "st. vincent" OR grenadines OR sudan OR suriname OR surinam OR "dutch guiana" OR "netherlands guiana" OR syria OR "syrian arab republic" OR tajikistan OR tadjikistan OR tadzhikistan OR tadzhik OR tanzania OR tanganyika OR thailand OR siam OR "timor leste" OR "east timor" OR togo OR "togolese republic" OR tonga OR "trinidad and tobago" OR trinidad OR tobago OR tunisia OR turkey OR "turkey (republic) " OR turkmenistan OR turkmen OR uganda OR ukraine OR uruguay OR uzbekistan OR uzbek OR vanuatu OR "new hebrides" OR venezuela OR vietnam OR "viet nam" OR "middle east" OR "west bank" OR gaza OR palestine OR yemen OR yugoslavia OR zambia OR zimbabwe OR "northern rhodesia" OR "global south" OR "africa south of the sahara" OR "sub-saharan africa" OR "subsaharan africa" OR "africa, central" OR "central africa" OR "africa, northern" OR "north africa" OR "northern africa" OR magreb OR maghrib OR sahara OR "africa, southern" OR "southern africa" OR "africa, eastern" OR "east africa" OR "eastern africa" OR "africa, western" OR "west africa" OR "western

africa" OR "west indies" OR "indian ocean islands" OR caribbean OR "central america" OR "latin america" OR "south and central america" OR "south america" OR "asia, central" OR "central asia" OR "asia, northern" OR "north asia" OR "northern asia" OR "asia, southeastern" OR "southeastern asia" OR "south eastern asia" OR "southeast asia" OR "south east asia" OR "asia, western" OR "western asia" OR "europe, eastern" OR "east europe" OR "eastern europe" OR "developing country" OR "developing countries" OR "developing nation" OR "developing nations" OR "developing population" OR "developing populations" OR "developing world" OR "less developed country" OR "less developed countries" OR "less developed nation" OR "less developed nations" OR "less developed population" OR "less developed populations" OR "less developed world" OR "lesser developed country" OR "lesser developed countries" OR "lesser developed nation" OR "lesser developed nations" OR "lesser developed population" OR "lesser developed populations" OR "lesser developed world" OR "under developed country" OR "under developed countries" OR "under developed nation" OR "under developed nations" OR "under developed population" OR "under developed populations" OR "under developed world" OR "underdeveloped country" OR "underdeveloped countries" OR "underdeveloped nation" OR "underdeveloped nations" OR "underdeveloped population" OR "underdeveloped populations" OR "underdeveloped world" OR "middle income country" OR "middle income countries" OR "middle income nation" OR "middle income nations" OR "middle income population" OR "middle income populations" OR "low income country" OR "low income countries" OR "low income nation" OR "low income nations" OR "low income population" OR "low income populations" OR "lower income country" OR "lower income countries" OR "lower income nation" OR "lower income nations" OR "lower income population" OR "lower income populations" OR "underserved country" OR "underserved countries" OR "underserved nation" OR "underserved nations" OR "underserved population" OR "underserved populations" OR "underserved world" OR "under served country" OR "under served countries" OR "under served nation" OR "under served nations" OR "under served population" OR " under served populations" OR "under served world" OR "deprived country" OR "deprived countries" OR "deprived nation" OR "deprived nations" OR "deprived population" OR "deprived populations" OR "deprived world" OR "poor country" OR "poor countries" OR "poor nation" OR "poor nations" OR "poor population" OR "poor populations" OR "poor world" OR "poorer country" OR "poorer countries" OR "poorer nation" OR "poorer nations" OR "poorer population" OR "poorer populations" OR "poorer world" OR "developing economy" OR "developing economies" OR "less developed economy" OR "less developed economies" OR "lesser developed economy" OR "lesser developed economies" OR "under developed economy" OR "under developed economies" OR "underdeveloped economy" OR "underdeveloped economies" OR "middle income economy" OR "middle income economies" OR "low income economy" OR "low income economies" OR "lower income economy" OR "lower income economies" OR "low gdp" OR "low gnp" OR "low gross domestic" OR "low gross national" OR "lower gdp" OR "lower gnp" OR "lower gross domestic" OR "lower gross national" OR lmic OR lmics OR "third world" OR "lami country" OR "lami countries" OR "transitional country" OR "transitional countries" OR "emerging economies" OR "emerging economy" OR "emerging nation" OR "emerging nations")

1. LMIC strategy as above

AND (TI,AB,SU(urban* OR city OR cities OR megacity OR megacities OR metropolitan OR metropolis OR megalopolis OR municipal* OR conurbation* OR suburb* OR town OR towns OR township* OR borough* OR barrio OR barrios) OR TI,AB,SU((dense* OR density OR high* OR large* OR heavy OR heavily) NEAR/3 (populat OR populous) NEAR/3 (area* OR settlement* OR district* OR neighbourhood* OR communit*)))) AND (TI,AB,SU(cost* NEAR/2 (illness* OR sickness* OR disease*)) OR TI,AB,SU(cost* NEAR/3 (care OR health OR healthcare OR medical*)) OR TI,AB,SU(expenditure* NEAR/3 (care OR health OR healthcare OR medical*)))
Date limit: 2010-01-01 - 2020-06-23
164 hits

2. LMIC strategy as above
AND (TI,AB,SU(urban* OR city OR cities OR megacity OR megacities OR metropolitan OR metropolis OR megalopolis OR municipal* OR conurbation* OR suburb* OR town OR towns OR township* OR borough* OR barrio OR barrios) OR TI,AB,SU((dense* OR density OR high* OR large* OR heavy OR heavily) NEAR/3 (populat OR populous) NEAR/3 (area* OR settlement* OR district* OR neighbourhood* OR communit*))) AND (TI,AB,SU((direct OR indirect) NEAR/2 (cost* OR expenditure*)) OR TI,AB,SU("out of pocket") OR TI,AB,SU(OOP NEAR/3 (cost* OR expense* OR expenditure* OR financ* OR pay* OR paid OR spend*)) OR TI,AB,SU(OOPE))
Date limit: 2010-01-01 - 2020-06-23
52 hits

3. LMIC strategy as above
AND (TI,AB,SU(urban* OR city OR cities OR megacity OR megacities OR metropolitan OR metropolis OR megalopolis OR municipal* OR conurbation* OR suburb* OR town OR towns OR township* OR borough* OR barrio OR barrios) OR TI,AB,SU((dense* OR density OR high* OR large* OR heavy OR heavily) NEAR/3 (populat OR populous) NEAR/3 (area* OR settlement* OR district* OR neighbourhood* OR communit*))) AND TI,AB,SU((personal OR individual* OR patient* OR family* OR families OR household*) NEAR/3 (budget* OR cost* OR expense* OR expenditure* OR financ* OR pay* OR paid OR spend*) NEAR/3 (care OR health OR healthcare OR medical*))
Date limit: 2010-01-01 - 2020-06-23
27 hits

4. LMIC strategy as above
AND (TI,AB,SU(urban* OR city OR cities OR megacity OR megacities OR metropolitan OR metropolis OR megalopolis OR municipal* OR conurbation* OR suburb* OR town OR towns OR township* OR borough* OR barrio OR barrios) OR TI,AB,SU((dense* OR density OR high* OR large* OR heavy OR heavily) NEAR/3 (populat OR populous) NEAR/3 (area* OR settlement* OR district* OR neighbourhood* OR communit*))) AND TI,AB,SU(catastroph* NEAR/10 (cost* OR expense* OR expenditure* OR financ* OR pay* OR paid OR spend*))
Date limit: 2010-01-01 - 2020-06-23
8 hits

5. LMIC strategy as above
AND (TI,AB,SU(urban* OR city OR cities OR megacity OR megacities OR metropolitan OR metropolis OR megalopolis OR municipal* OR conurbation* OR suburb* OR town OR towns OR township* OR borough* OR barrio OR barrios) OR TI,AB,SU((dense* OR density OR high* OR large* OR heavy OR heavily) NEAR/3 (populat OR populous) NEAR/3 (area* OR settlement* OR district* OR neighbourhood* OR communit*))) AND TI,AB,SU((willing* OR unwilling*) NEAR/3 (pay*) NEAR/3 (care OR health OR healthcare OR medical*))
Date limit: 2010-01-01 - 2020-06-23
1 hit

6. LMIC strategy as above
AND (TI,AB,SU(urban* OR city OR cities OR megacity OR megacities OR metropolitan OR metropolis OR megalopolis OR municipal* OR conurbation* OR suburb* OR town OR towns OR township* OR borough* OR barrio OR barrios) OR TI,AB,SU((dense* OR density OR high* OR large* OR heavy OR heavily) NEAR/3 (populat OR populous) NEAR/3 (area* OR settlement* OR district* OR neighbourhood* OR communit*))) AND (TI,AB,SU((medical* OR health) NEAR/3 impoverish*) OR TI,AB,SU(burden* NEAR/3 (cost* OR expenditure* OR economic* OR financ*) NEAR/3 (care OR

For peer review only

health OR healthcare OR medical*)) OR TI,AB,SU((afford* OR unafford*) NEAR/5 (cost* OR pay*)) OR TI,AB,SU((able OR ability OR capacit* OR unable OR inability) NEAR/2 pay*))
Date limit: 2010-01-01 - 2020-06-23
81 hits

7. LMIC strategy as above
AND (TI,AB,SU(slum OR slums OR ghetto OR ghettos OR shanty* OR shanties OR shack* OR favela*) OR TI,AB,SU((informal* OR precarious* OR irregular*) NEAR/3 settlement*) OR TI,AB,SU((poverty OR impoverish* OR squatter*) NEAR/3 (area* OR settlement*))) AND ((TI,AB,SU(cost* NEAR/2 (illness* OR sickness* OR disease*)) OR TI,AB,SU(cost* NEAR/3 (care OR health OR healthcare OR medical*)) OR TI,AB,SU(expenditure* NEAR/3 (care OR health OR healthcare OR medical*))) OR (TI,AB,SU((direct OR indirect) NEAR/2 (cost* OR expenditure*)) OR TI,AB,SU("out of pocket") OR TI,AB,SU(OOP NEAR/3 (cost* OR expense* OR expenditure* OR financ* OR pay* OR paid OR spend*)) OR TI,AB,SU(OOPE)))
Date limit: 2010-01-01 - 2020-06-23
15 hits

8. LMIC strategy as above
AND (TI,AB,SU(slum OR slums OR ghetto OR ghettos OR shanty* OR shanties OR shack* OR favela*) OR TI,AB,SU((informal* OR precarious* OR irregular*) NEAR/3 settlement*) OR TI,AB,SU((poverty OR impoverish* OR squatter*) NEAR/3 (area* OR settlement*))) AND TI,AB,SU((personal OR individual* OR patient* OR family* OR families OR household*) NEAR/3 (budget* OR cost* OR expense* OR expenditure* OR financ* OR pay* OR paid OR spend*) NEAR/3 (care OR health OR healthcare OR medical*))
Date limit: 2010-01-01 - 2020-06-23
3 hits

9. LMIC strategy as above
AND TI,AB,SU(slum OR slums OR ghetto OR ghettos OR shanty* OR shanties OR shack* OR favela*) OR TI,AB,SU((informal* OR precarious* OR irregular*) NEAR/3 settlement*) OR TI,AB,SU((poverty OR impoverish* OR squatter*) NEAR/3 (area* OR settlement*))) AND TI,AB,SU(catastroph* NEAR/10 (cost* OR expense* OR expenditure* OR financ* OR pay* OR paid OR spend*))
Date limit: 2010-01-01 - 2020-06-23
2 hits

10. LMIC strategy as above
AND (TI,AB,SU(slum OR slums OR ghetto OR ghettos OR shanty* OR shanties OR shack* OR favela*) OR TI,AB,SU((informal* OR precarious* OR irregular*) NEAR/3 settlement*) OR TI,AB,SU((poverty OR impoverish* OR squatter*) NEAR/3 (area* OR settlement*))) AND (TI,AB,SU((willing* OR unwilling*) NEAR/3 (pay*) NEAR/3 (care OR health OR healthcare OR medical*)) OR (TI,AB,SU((medical* OR health) NEAR/3 impoverish*) OR TI,AB,SU(burden* NEAR/3 (cost* OR expenditure* OR economic* OR financ*) NEAR/3 (care OR health OR healthcare OR medical*)) OR TI,AB,SU((afford* OR unafford*) NEAR/5 (cost* OR pay*)) OR TI,AB,SU((able OR ability OR capacit* OR unable OR inability) NEAR/2 pay*)))
Date limit: 2010-01-01 - 2020-06-23
9 hits

Key:
TI,AB,SU = terms in title, abstract, or subject heading fields
* = truncation
" " = exact phrase search

For peer review only

NEAR/3 = terms within three words of each other (any order)

Econpapers
https://econpapers.repec.org/

OpenGrey
http://www.opengrey.eu/

World Bank

OECD

References

1. Effective Practice and Organisation of Care (EPOC) Cochrane group. LMIC filters. The Cochrane Collaboration. 2020 [accessed 14th May 2020]. Available from: https://epoc.cochrane.org/lmic-filters

For peer review only

Reporting checklist for protocol of a systematic review.

Based on the PRISMA-P guidelines.

Instructions to authors

Complete this checklist by entering the page numbers from your manuscript where readers will find each of the items listed below.

Your article may not currently address all the items on the checklist. Please modify your text to include the missing information. If you are certain that an item does not apply, please write "n/a" and provide a short explanation.

Upload your completed checklist as an extra file when you submit to a journal.

In your methods section, say that you used the PRISMA-Preporting guidelines, and cite them as:

Moher D, Shamseer L, Clarke M, Ghersi D, Liberati A, Petticrew M, Shekelle P, Stewart LA. Preferred Reporting Items for Systematic Review and Meta-Analysis Protocols (PRISMA-P) 2015 statement. Syst Rev. 2015;4(1):1.

		Reporting Item	Page Number
**Title**			
Identification	#1a	Identify the report as a protocol of a systematic review	1
Update	#1b	If the protocol is for an update of a previous systematic review, identify as such	N/A

For peer review only

Registration

	#2	If registered, provide the name of the registry (such as PROSPERO) and registration number	5

Authors

Contact	#3a	Provide name, institutional affiliation, e-mail address of all protocol authors; provide physical mailing address of corresponding author	1
Contribution	#3b	Describe contributions of protocol authors and identify the guarantor of the review	9

Amendments

	#4	If the protocol represents an amendment of a previously completed or published protocol, identify as such and list changes; otherwise, state plan for documenting important protocol amendments	N/A

Support

Sources	#5a	Indicate sources of financial or other support for the review	9
Sponsor	#5b	Provide name for the review funder and / or sponsor	9
Role of sponsor or funder	#5c	Describe roles of funder(s), sponsor(s), and / or institution(s), if any, in developing the protocol	9

Introduction

For peer review only

Rationale	#6	Describe the rationale for the review in the context of what is already known	4
Objectives	#7	Provide an explicit statement of the question(s) the review will address with reference to participants, interventions, comparators, and outcomes (PICO)	5
**Methods**			
Eligibility criteria	#8	Specify the study characteristics (such as PICO, study design, setting, time frame) and report characteristics (such as years considered, language, publication status) to be used as criteria for eligibility for the review	7
Information sources	#9	Describe all intended information sources (such as electronic databases, contact with study authors, trial registers or other grey literature sources) with planned dates of coverage	6
Search strategy	#10	Present draft of search strategy to be used for at least one electronic database, including planned limits, such that it could be repeated	6
Study records - data management	#11a	Describe the mechanism(s) that will be used to manage records and data throughout the review	6
Study records - selection process	#11b	State the process that will be used for selecting studies (such as two independent reviewers) through each phase of the review (that is, screening, eligibility and inclusion in meta-analysis)	7

For peer review only

Study records - data collection process	#11c	Describe planned method of extracting data from reports (such as piloting forms, done independently, in duplicate), any processes for obtaining and confirming data from investigators	7
Data items	#12	List and define all variables for which data will be sought (such as PICO items, funding sources), any pre-planned data assumptions and simplifications	6, 8
Outcomes and prioritization	#13	List and define all outcomes for which data will be sought, including prioritization of main and additional outcomes, with rationale	8
Risk of bias in individual studies	#14	Describe anticipated methods for assessing risk of bias of individual studies, including whether this will be done at the outcome or study level, or both; state how this information will be used in data synthesis	7
Data synthesis	#15a	Describe criteria under which study data will be quantitatively synthesised	8
Data synthesis	#15b	If data are appropriate for quantitative synthesis, describe planned summary measures, methods of handling data and methods of combining data from studies, including any planned exploration of consistency (such as I2, Kendall's τ)	8
Data synthesis	#15c	Describe any proposed additional analyses (such as sensitivity or subgroup analyses, meta-regression)	8

For peer review only

Data synthesis	#15d	If quantitative synthesis is not appropriate, describe the type of summary planned	N/A
Meta-bias(es)	#16	Specify any planned assessment of meta-bias(es) (such as publication bias across studies, selective reporting within studies)	N/A
Confidence in cumulative evidence	#17	Describe how the strength of the body of evidence will be assessed (such as GRADE)	N/A

The PRISMA-P checklist is distributed under the terms of the Creative Commons Attribution License CC-BY 4.0. This checklist was completed on 30. September 2020 using https://www.goodreports.org/, a tool made by the EQUATOR Network in collaboration with Penelope.ai

For peer review only
